# Synthesis of tetrafluorinated piperidines from nitrones via a visible-light-promoted annelation reaction

**DOI:** 10.3762/bjoc.16.260

**Published:** 2020-12-29

**Authors:** Vyacheslav I Supranovich, Igor A Dmitriev, Alexander D Dilman

**Affiliations:** 1 N. D. Zelinsky Institute of Organic Chemistry, 119991 Moscow, Leninsky prosp. 47, Russian Federation,; 2 Moscow State University, Department of Chemistry, 119991, Moscow, Leninskie Gory 1–3, Russian Federation

**Keywords:** difluoroalkylation, nitrones, organofluorine compounds, photocatalysis, radical addition

## Abstract

A method for the one-step construction of 3,3,4,4-tetrafluorinated piperidines from nitrones and readily accessible tetrafluorinated iodobromobutane is described. The reaction requires an excess amount of ascorbic acid as the terminal reductant and is performed in the presence of an iridium photocatalyst activated by blue light. The annelation is a result of a radical addition at the nitrone, intramolecular nucleophilic substitution, and reduction of the N–O bond.

## Introduction

Nitrogen-containing heterocyclic compounds play an important role in pharmaceutical industry and related areas [1–2]. Among the variety of aromatic and saturated structures, the piperidine ring has a special role, as it is the most widely occurring form of nitrogen in FDA-approved drugs [3]. The ability of fluorine atoms to modify basicity, lipophilicity, as well as the hydrogen-bonding properties of amines [4–5] makes fluorinated piperidines [6–7] attractive targets in medicinal chemistry [8–9]. Previous efforts were mainly focused on the synthesis of mono- and difluorinated compounds. A single fluorine atom is typically introduced into a saturated cycle by nucleophilic fluorination reactions [10–17]. For compounds bearing *gem*-difluorinated fragments, an alternative pathway based on the construction of the cycle from building blocks may be considered [18]. However, the latter frequently implies multistep protocols, since two distinct bond-forming reactions are necessary for the cycle formation. While mono- and difluorinated piperidines are well known, tetrafluorinated piperidines are rare [19]. At the same time, the chemistry of compounds containing the tetrafluoroethylene fragment (CF_2_CF_2_) has advanced considerably over the last decade [20], and the use of preformed tetrafluorinated building blocks [21] provides the most efficient way of making these molecules.

**Scheme 1:**
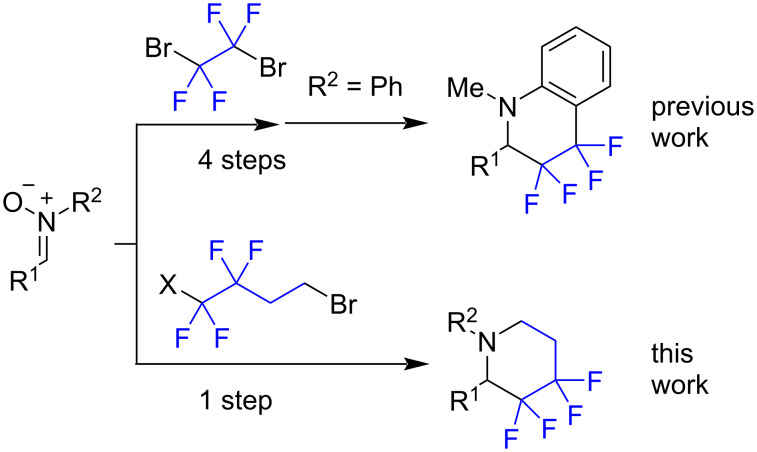
The construction of tetrafluorinated piperidines from nitrones.

Recently, we disclosed a photoredox method for the reductive radical fluoroalkylation of nitrones [22–24]. We have also evaluated the construction of fluorinated tetrahydroisoquinoline structures starting from nitrones, but this required four consecutive reactions with different conditions and chromatographic separation at each step [25] (Scheme 1). Herein we report a single-step protocol for the construction of fluorinated piperidines based on an accidentally discovered annelation reaction proceeding under reductive conditions.

## Results and Discussion

Nitrone **1a** was selected as a model substrate and it was combined with commercially available dibromide **2a** under blue light irradiation in the presence of an iridium photocatalyst and stoichiometric quantities (1.2 equiv) of a reducing system (ascorbic acid/collidine). In this reaction no products were formed with reactant **2a** remaining unconsumed (Table 1). To obtain a more reactive fluorinated halide, the bromine atom residing at the fluorinated moiety was exchanged for iodine by treatment with zinc, followed by the reaction with iodine monochloride. The iodide **2b** reacted quite rapidly with the nitrone but the expected fluoroalkylation product was not observed. Instead, the tetrafluorinated piperidine **3a** was obtained in a moderate yield (Table 1, entry 2). Apparently, after the fluoroalkylation event, the reduction of the N–O bond had occurred. The corresponding addition of an additional amount of the reductant and performing the reaction in DMF led to product **3a** in 84% isolated yield (Table 1, entry 4).

**Table 1:** Optimization studies for the synthesis of **3a**.

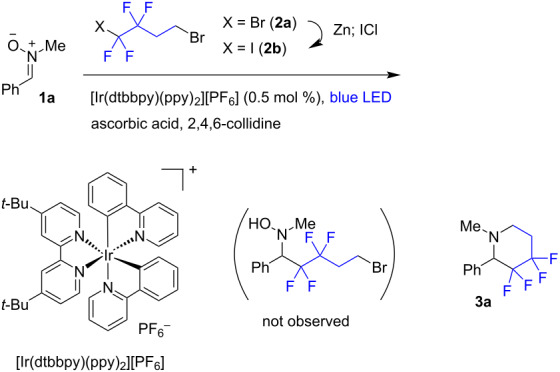					

entry	**2**^a^	ascorbic acid^a^	collidine^a^	solvent	yield, %

1	**2a**, 1.2	1.2	1.2	DMSO	–
2	**2b**, 1.2	1.2	1.2	DMSO	56^b^
3	**2b**, 1.5	2.5	3.5	DMSO	70^b^
4	**2b**, 1.5	2.5	3.5	DMF	84^c^

^a^Equivalents are shown; ^b^determined by ^19^F NMR; ^c^isolated yield.

**Scheme 2:**
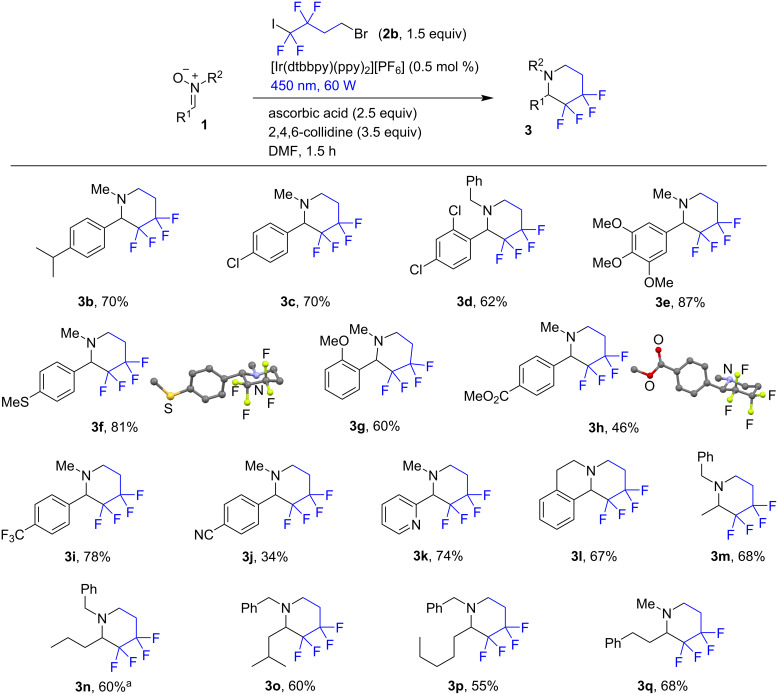
The scope of the annelation reaction for the synthesis of piperidines. Isolated yields are shown. ^a^1.0 equiv of **2b** was used.

Under the optimized conditions, a series of nitrones were involved in the reductive annelation reaction (Scheme 2). The process worked with nitrones containing alkyl, halogen, and electron-donating groups in the aromatic ring. With ester and cyano groups, the piperidines **3h** and **3j** were obtained in decreased yields, which may be tentatively attributed to a greater propensity of the corresponding nitrones towards nucleophilic addition of ascorbate. The nitrones derived from α-unbranched aliphatic aldehydes also provided the expected piperidines (products **3m–q**) in reasonable yields. In case of **3n** there was some unidentified side product formed under the standard conditions. In the previous research on the radical addition to nitrones we sometimes encountered a fluoroalkylation of the aromatic rings, if an excess of the fluorinated alkyl iodide was used [22–24]. This prompted us to attempt the reaction with an equimolar amount of the iodide **2b**, which resulted in a cleaner process. However, the reaction of a nitrone obtained from cyclohexyl carboxaldehyde gave a complex mixture containing unidentified products. The structures of compounds **3f** and **3h** were established by X-ray analysis (CCDC, **3f**: 2032382; **3h**: 2032383).

**Scheme 3:**
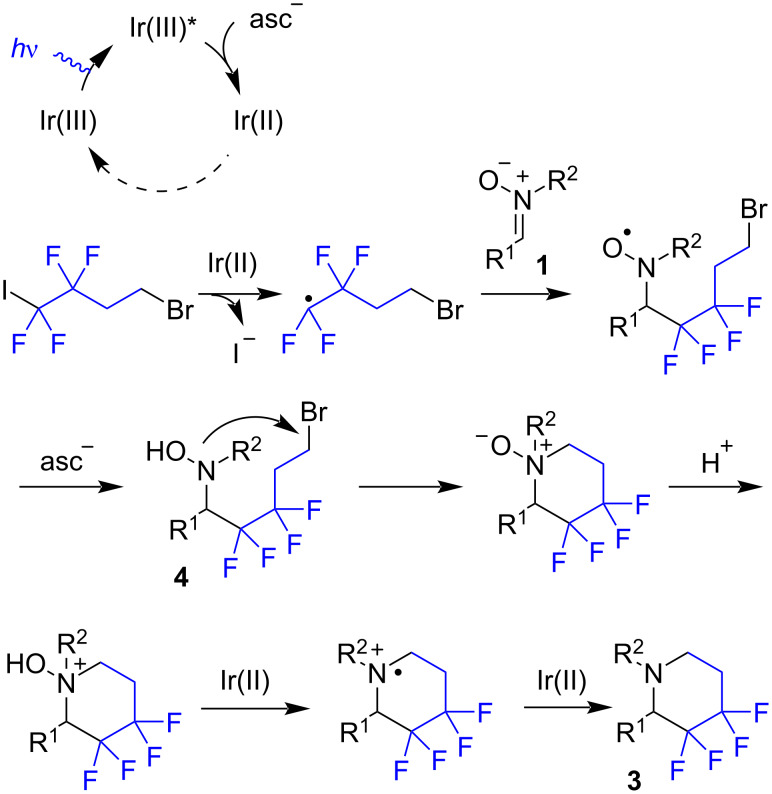
The proposed mechanism of the photoredox annelation reaction (asc = ascorbic acid).

The proposed annelation mechanism is shown in Scheme 3. The iridium(III) photocatalyst under the action of light and ascorbic acid generates the iridium(II) species. The latter serves as a key reducing agent, and importantly, its formation is maintained throughout the process while an excess amount of ascorbate is present. The annelation likely starts from the addition of the fluorinated radical to the C=N bond followed by a conversion of the nitroxyl radical via hydrogen atom transfer [26–27] providing hydroxylamine **4**. At the next stage, the intramolecular N-alkylation occurs leading to an *N*-oxide. This step of nucleophilic substitution could be catalyzed by iodide anions accumulating in the reaction mixture. Finally, the deoxygenation of the *N*-oxide fragment may proceed via consecutive protonation and electron-transfer steps [28].

## Conclusion

In summary, a one-step method for the synthesis of tetrafluorinated piperidines starting from nitrones and a fluorinated building block is described. The annelation is based on a sequence of visible-light-promoted redox processes and a substitution reaction, and involves the cleavage of the N–O bond.

## Supporting Information

File 1 Full experimental details, compound characterization, X-ray data, and copies of NMR spectra.

File 2 Crystallographic information file for compound **3f**.

File 3 Crystallographic information file for compound **3h**.
