# Supplementary material for: Synthesis of tetrafluorinated piperidines from nitrones via a visible-light-promoted annelation reaction
Source: Beilstein J Org Chem. 2020 Dec 29;16:3104–8. doi: 10.3762/bjoc.16.260 (PMC7783028; doi:10.3762/bjoc.16.260)

BEILSTEIN JOURNAL OF ORGANIC CHEMISTRY

Supporting Information

for

Synthesis of tetrafluorinated piperidines from nitrones via a visible-light-promoted annelation reaction

Vyacheslav I. Supranovich, Igor A. Dmitriev and Alexander D. Dilman

Beilstein J. Org. Chem. **2020,** *16,* 3104–3108. doi:10.3762/bjoc.16.260

Full experimental details, compound characterization, X-ray data, and copies of NMR spectra

License and Terms: This is a supporting information file under the terms of the Creative Commons Attribution License (https://creativecommons.org/licenses/by/4.0). Please note that the reuse, redistribution and reproduction in particular requires that the author(s) and source are credited and that individual graphics may be subject to special legal provisions.
The license is subject to the *Beilstein Journal of Organic Chemistry* terms and conditions: (https://www.beilstein-journals.org/bjoc/terms)

Table of contents

	Page
General methods	S2
Procedures, compound characterization data	S2–S12
X-ray data	S13
References	S14
NMR spectra	S15–S72

General methods. DMF was distilled from $MgSO_4$. Acetonitrile was distilled from P_2O_5 and stored over 3 Å molecular sieves. Column chromatography was carried out employing silica gel (230−400 mesh). Precoated silica gel plates F-254 were used for thin-layer analytical chromatography and visualizing with UV and/or acidic aq. $KMnO_4$ solution. NMR spectra were recorded on a Bruker Avance II 300 spectrometer. High resolution mass spectra (HRMS) were measured using electrospray ionization (ESI) and a time-of-flight (TOF) mass analyzer. The measurements were done in the positive ion mode (interface capillary voltage −4500 V) or in the negative ion mode (3200 V); mass range from *m*/*z* 50 to *m*/*z* 3000. Melting points were measured with a Stuart SMP30.

Reagents. Nitrones were prepared according to literature procedures: *N*-(benzylidene)methylamine *N*-oxide (**1a**),[1] *N*-(4-isopropylbenzylidene)methanamine *N*-oxide (**1b**),[2] *N*-(4-chlorobenzylidene)methanamine *N*-oxide (**1c**),[3] *N*-(2,4-dichlorobenzylidene)-1-benzylamine *N*-oxide (**1d**),[2] *N*-(3,4,5-trimethoxybenzylidene)-methanamine *N*-oxide (**1e**),[4] *N*-(2-methoxybenzylidene)methanamine *N*-oxide (**1g**),[5] *N*-[4-(methoxy-carbonyl)benzylidene]methanamine *N*-oxide (**1h**),[6] *N*-(4-(trifluoromethyl)benzylidene)methanamine *N*-oxide (**1i**),[7] *N*-(4-cyanobenzylidene)methanamine *N*-oxide (**1j**),[8] *N*-(2-pyridylene)-methylamine *N*-oxide (**1k**),[5] 3,4-dihydroisoquinoline *N*-oxide (**1l**),[9] *N*-ethylidenebenzylamine *N*-oxide (**1m**),[10] *N*-butylidenebenzylamine *N*-oxide (**1n**),[11] *N*-(3-methylbutylidene)benzylamine *N*-oxide (**1o**),[11] *N*-(3-phenylpropylidene)methanamine *N*-oxide (**1q**).[12]

N-[4-(Methylthio)benzylidene]methylamine *N*-oxide (1f).[13]

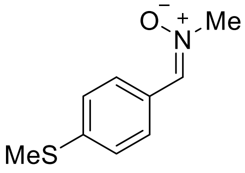

1f

4-(Methylthio)benzaldehyde (304 mg, 2.0 mmol), *N*-methyl hydroxylamine hydrochloride (252 mg, 3.0 mmol, 1.5 equiv), and $NaHCO_3$ (252 mg, 3.0 mmol, 1.5 equiv) were stirred in dichloromethane (5 mL) at room temperature overnight. The mixture was poured into water (30 mL), the organic phase was separated, and the aqueous layer was additionally extracted with dichloromethane (2 × 5 mL). The combined organic phases were dried with $MgSO_4$, filtered, and evaporated, and the residue was washed on a filter with hot hexanes affording the product as colorless crystals (295 mg, 82%). Mp 106–107 °C.

^{1}H NMR (300 MHz, $CDCl_3$) δ: 8.10 (d, *J* = 8.6 Hz, 2H), 7.27 (s, 1H), 7.20 (d, *J* = 8.6 Hz, 2H), 3.80 (s, 3H), 2.45 (s, 3H).

^{13}C{^{1}H} NMR (75 MHz, $CDCl_3$) δ: 141.8, 134.7, 128.6, 127.0, 125.3, 54.1, 14.9.

N-Hexylidenebenzylamine *N*-oxide (1p).[14]

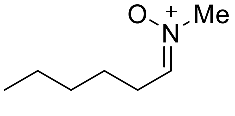

1p

Hexanal (180 mg, 1.8 mmol) was added to a stirred mixture of *N*-benzylhydroxylamine hydrochloride (240 mg, 1.5 mmol) and $NaHCO_3$ (252 mg, 3.0 mmol) in dichloromethane (5 mL), and the mixture was stirred for 24 hours at room temperature. The mixture was poured into water (50 mL), the organic phase was separated,

and the aqueous layer was additionally extracted with dichloromethane (3 × 5 mL). The combined organic phases were dried with $MgSO_4$ and evaporated. The crude product was recrystallized from hexanes to afford 200 mg of colorless crystals (65 %). Mp 84–85 °C.

^{1}H NMR (300 MHz, $CDCl_3$) δ: 7.39–7.29 (m, 5H), 6.62 (t, *J* = 5.9 Hz, 1H), 4.81 (s, 2H), 2.42 (td, *J* = 7.4, 5.9 Hz, 2H), 1.48–1.38 (m, 2H), 1.30–1.20 (m, 4H), 0.83 (t, *J* = 6.9 Hz, 3H).

^{13}C{^{1}H} NMR (75 MHz, $CDCl_3$) δ: 139.4, 133.0, 129.0, 128.7, 128.6, 68.9, 31.5, 26.5, 25.0, 22.1, 13.8.

4-Bromo-1,1,2,2-tetrafluoro-1-iodobutane (**2b**).

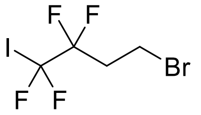

2b

A Schlenk tube containing zinc dust (2.60 g, 40.0 mmol) and a magnetic stirrer was dried under vacuum with a heat gun and allowed to cool under argon atmosphere. Acetonitrile (5 mL) and two drops of chlorotrimethylsilane were added and the tube was heated in a 60 °C bath for 15 minutes while stirring. The heating bath was removed, and the tube was cooled in a water/ice bath. Then, 1,4-dibromo-1,1,2,2-tetrafluorobutane (5.86 g, 20.3 mmol) in acetonitrile (17 mL) was added in small portions in about 45 minutes. The bath was allowed to warm to room temperature and the reaction mixture was stirred overnight (around 16 hours). The mixture was filtered from excess of zinc through a cotton plug, and the resulting clear solution was handled open to air atmosphere without special precautions. The mixture was cooled with an ice/water bath. A solution of iodine monochloride (6.48 g, 40 mmol) in dichloromethane (10 mL) was added dropwise with a syringe in about 10 min, and the cooling bath was removed. After stirring for 24 hours at room temperature, the mixture was poured into a separatory funnel containing water (300 mL). Sodium thiosulfate hydrate (7.5 g) was added in portions and the mixture was shaken until complete discoloration occurred. The mixture was extracted with pentane (3 × 20 mL), the combined organic phases were dried over $MgSO_4$, and carefully evaporated at atmospheric pressure (40 °C water bath). The residue was distilled (bp 63–64 °C/20 Torr) to yield a slightly pink-colored liquid (4.61 g, 68%).

^{1}H NMR (300 MHz, $CDCl_3$) δ: 3.53 (t, *J* = 7.9 Hz, 2H), 2.71 (tt, *J* = 16.5, 8.1 Hz, 2H).

^{13}C {^{1}H} NMR (75 MHz, $CDCl_3$) δ: 116.3 (tt, *J* = 254.7, 31.2 Hz), 96.9 (tt, *J* = 317.5, 42.6 Hz), 33.5 (t, *J* = 22.6 Hz), 20.9 (tt, *J* = 4.7, 1.2 Hz).

^{19}F NMR (282 MHz, $CDCl_3$) δ: -61.1 (t, *J* = 4.9 Hz, 2F), -108.5 (tt, *J* = 16.8, 4.9 Hz, 2F).

Calcd for $C_4H_4BrF_4I$ (334.88): C, 14.35; H, 1.20. Found: C, 14.51; H, 1.28.

Reaction of nitrones with 2b (general procedure). Nitrone **1** (0.50 mmol), ascorbic acid (220 mg, 1.25 mmol, 2.5 equiv), [Ir(ppy)$_2$(dtbbpy)][PF$_6$] (2.3 mg, 0.0025 mmol, 0.5 mol %) were placed in a tube (Duran, cat. # 261351258, Roth cat. no PY94.1, outside diameter 13 mm). The tube was evacuated and filled with argon and DMF (2.0 mL) was added. The tube was briefly evacuated and refilled with argon. Then, iodide **2b** (for **3a–m**,**o–q**, 251 mg, 0.75 mmol, 1.5 equiv; for **3n**, 168 mg, 0.5 mmol, 1.0 equiv) and 2,4,6-collidine (212 mg, 1.75 mmol, 3.5 equiv) were added. The tube was closed with a screw cap and irradiated for 1.5 hours by a 450 nm LED chip (Hontiey royal blue 100W, operated at 60 Watt). The distance between the LED chip and the reaction tube was 1 cm. During the reaction, the bath temperature was maintained in a range of 15–20 °C. The reaction mixture was poured into water (15 mL) and extracted with hexanes (3 × 4 mL). The combined organic phases were dried over Na_2SO_4, filtered, evaporated, and the residue was purified by column chromatography on silica gel.

3,3,4,4-Tetrafluoro-1-methyl-2-phenylpiperidine (**3a**).

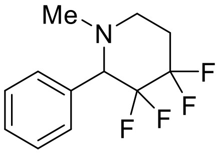

3a

Yield 104 mg (84 %). Colorless oil.

Chromatography: hexanes/EtOAc, from 5/1 to 1/1. R_f 0.18 (hexanes/EtOAc, 7/1).

^{1}H NMR (300 MHz, $CDCl_3$) δ: 7.49–7.37 (m, 5H), 3.40 (dddd, *J* = 21.7, 5.0, 3.7, 1.1 Hz, 1H), 3.13–2.99 (m, 1H), 2.62–2.18 (m, 3H), 2.10 (s, 3H).

^{13}C{^{1}H} NMR (75 MHz, $CDCl_3$) δ: 132.9, 130.3 (d, *J* = 1.7 Hz), 129.1, 128.5, 115.9 (dddd, *J* = 260.5, 244.3, 32.4, 21.4 Hz), 113.4 (dddd, *J* = 254.4, 252.3, 30.2, 20.6 Hz), 71.1 (dddd, *J* = 24.9, 18.9, 3.2, 1.3 Hz), 51.4 (d, *J* = 10.5 Hz), 43.2 (t, *J* = 1.4 Hz), 31.3 (dddd, *J* = 21.2, 19.9, 3.4, 1.4 Hz).

^{19}F NMR (282 MHz, $CDCl_3$) δ: -119.3 (dm, *J* = 248.9 Hz, 1F), -122.1 (dm, *J* = 248.9 Hz, 1F), -126.2 (ddtd, *J* = 256.5, 21.4, 16.3, 4.4 Hz, 1F), -130.1 (dddt, *J* = 256.5, 16.4, 14.6, 5.0 Hz).

HRMS (ESI): calcd for $C_{12}H_{14}F_4N$ (M+H) 248.1057, found 248.1050.

3,3,4,4-Tetrafluoro-2-(4-isopropylphenyl)-1-methylpiperidine (3b).

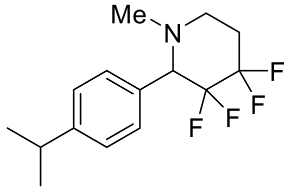

3b

Yield 102 mg (70 %). Colorless crystals. Mp 49–50 °C.

Chromatography: hexanes/EtOAc, 5/1. R_f 0.25 (hexanes/EtOAc, 5/1).

^{1}H NMR (300 MHz, $CDCl_3$) δ: 7.37 (dd, *J* = 8.3, 1.7 Hz, 2H), 7.26 (d, *J* = 8.3 Hz, 2H), 3.38 (dt, *J* = 21.9, 4.6 Hz, 1H), 3.11–3.01 (m, 1H), 2.95 (sept, *J* = 6.9 Hz, 1H), 2.60–2.18 (m, 3H), 2.11 (s, 3H), 1.30 (d, *J* = 6.9 Hz, 6H).

^{13}C{^{1}H} NMR (75 MHz, $CDCl_3$) δ: 149.7, 130.2 (d, *J* = 1.7 Hz), 130.2, 126.5, 116.0 (dddd, *J* = 260.5, 244.2, 32.4, 21.4 Hz), 113.5 (dddd, *J* = 254.5, 251.9, 29.8, 20.1 Hz), 70.8 (dddd, *J* = 25.1, 19.1, 3.2, 1.3 Hz), 51.3 (d, *J* = 10.5 Hz), 43.2 (t, *J* = 1.4 Hz), 34.0, 31.3 (tdd, *J* = 20.8, 3.1, 1.4 Hz), 24.04, 24.00.

^{19}F NMR (282 MHz, $CDCl_3$) δ: -119.2 (dm, *J* = 248.2, 1F), -122.0 (dm, *J* = 248.2 Hz, 1F), -126.2 (ddtd, *J* = 256.9, 21.3, 16.8, 15.7, 4.2 Hz, 1F), -130.1 (dddt, *J* = 257.0, 19.1, 14.7, 4.7 Hz, 1F).

HRMS (ESI): calcd for $C_{15}H_{20}F_4N$ (M+H) 290.1526, found 290.1521.

2-(4-Chlorophenyl)-3,3,4,4-tetrafluoro-1-methylpiperidine (3c).

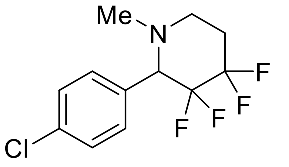

3c

Yield 98 mg (70 %). Yellowish crystals. Mp 52–53 °C.

Chromatography: hexanes/EtOAc, from 5/1 to 1/1. R_f 0.25 (hexanes/EtOAc, 4/1).

^{1}H NMR (300 MHz, $CDCl_3$) δ: 7.39 (s, 4H), 3.39 (dt, *J* = 21.3, 4.5 Hz, 1H), 3.10–3.01 (m, 1H), 2.61–2.17 (m, 3H), 2.09 (s, 3H).

^{13}C{^{1}H} NMR (75 MHz, $CDCl_3$) δ: 134.99, 131.53 (d, *J* = 1.8 Hz), 131.42, 128.67, 115.70 (dddd, *J* = 260.7, 244.2, 32.2, 21.3 Hz), 113.14 (dddd, *J* = 254.8, 252.3, 30.3, 20.7 Hz), 70.34 (dddd, *J* = 25.0, 19.1, 3.3, 1.3 Hz), 51.16 (d, *J* = 10.5 Hz), 43.08 (t, *J* = 1.3 Hz), 31.15 (dddd, *J* = 21.2, 19.9, 3.4, 1.3 Hz).

^{19}F NMR (282 MHz, $CDCl_3$) δ: -119.6 (dm, J = 249.0 Hz, 1F), -122.2 (dm, *J* = 249.0 Hz, 1F), -126.2 (dm, *J* = 257.0 Hz, 1F), -130.1 (dddt, *J* = 257.0, 17.5, 14.1, 4.9 Hz, 1F).

HRMS (ESI): calcd for $C_{12}H_{13}{}^{35}ClF_4N$ (M+H) 282.0667, found 282.0666; calcd for $C_{12}H_{13}{}^{37}ClF_4N$ (M+H) 284.0638, found 284.0641.

1-Benzyl-2-(2,4-dichlorophenyl)-3,3,4,4-tetrafluoropiperidine (3d).

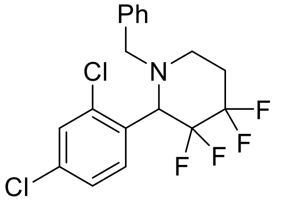

3d

Yield 121 mg (62 %). Colorless crystals. Mp 113–114°C.

Chromatography: hexanes/EtOAc, 20/1. R_f 0.30 (hexanes/EtOAc, 20/1). Product isolated by chromatography was recrystallized from hexanes.

^{1}H NMR (300 MHz, $CDCl_3$) δ: 7.79 (dd, *J* = 8.5, 3.2 Hz, 1H), 7.43 (s, 1H), 7.35–7.06 (m, 6H), 4.42 (d, *J* = 21.1 Hz, 1H), 3.61 (d, *J* = 13.6 Hz, 1H), 2.96 (d, *J* = 13.7 Hz, 2H), 2.49–2.00 (m, 3H).

^{13}C{^{1}H} NMR (75 MHz, $CDCl_3$) δ: 137.3 (d, *J* = 0.9 Hz), 136.7, 135.5, 132.6, 132.5, 129.7, 129.5 (d, *J* = 1.1 Hz), 128.7, 128.5, 127.7, 115.9 (dddd, *J* = 260.1, 244.5, 32.3, 20.9 Hz), 113.8 (dddd, *J* = 257.4, 252.8, 30.9, 21.2 Hz), 63.9 (ddd, *J* = 24.6,17.7, 2.7 Hz), 58.0 (t, *J* = 1.2 Hz), 47.4 (d, *J* = 10.4 Hz), 31.0 (td, *J* = 21.5, 0.9 Hz).

^{19}F NMR (282 MHz, $CDCl_3$) δ: -119.7 (dm, *J* = 249.1 Hz, 1F), -122.2 (dt, *J* = 249.1, 15.2 Hz, 1F), -124.9 (dq, *J* = 257.8, 17.4 Hz, 1F), -130.2 (dt, *J* = 257.8, 13.9 Hz, 1F).

HRMS (ESI): calcd for $C_{18}H_{16}{}^{35}Cl_2F_4N$ (M+H) 392.0590, found 392.0578; calcd for $C_{18}H_{16}{}^{37}Cl^{35}ClF_4N$ (M+H) 394.0562, found 394.0552.

3,3,4,4-Tetrafluoro-1-methyl-2-(3,4,5-trimethoxyphenyl)piperidine (3e).

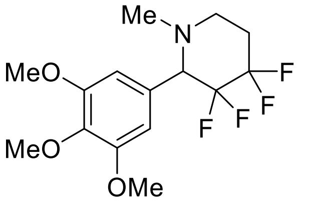

3e

Yield 147 mg (87 %). Colorless crystals. Mp 97.8–98.5°C.

Chromatography: hexanes/EtOAc, 1/1. R_f 0.25 (hexanes/EtOAc, 1/1).

^{1}H NMR (300 MHz, $CDCl_3$) δ: 6.64 (d, *J* = 1.4 Hz, 2H), 3.86 (s, 9H), 3.28 (dt, *J* = 21.5, 4.3 Hz, 1H), 3.10–2.96 (m, 1H), 2.55–2.16 (m, 3H), 2.10 (s, 3H).

^{13}C{^{1}H} NMR (75 MHz, $CDCl_3$) δ: 153.2, 138.6, 128.2, 115.8 (dddd, *J* = 260.7, 244.4, 32.4, 21.4 Hz), 113.3 (dddd, *J* = 254.5, 251.9, 29.8, 20.1 Hz), 107.3 (d, *J* = 1.9 Hz), 71.1 (dddd, *J* = 25.2, 18.9, 3.1, 1.4 Hz), 60.9, 56.2, 51.2 (d, *J* = 10.5 Hz), 43.1 (t, *J* = 1.4 Hz), 31.07 (tdd, *J* = 20.7, 3.6, 1.2 Hz).

^{19}F NMR (282 MHz, $CDCl_3$) δ: -119.3 (dm, *J* = 248.7, 1F), -122.1 (dm, *J* = 248.7 Hz, 1F), -126.1 (dtdd, *J* = 255.9, 21.4, 17.8, 14.1, 4.3 Hz, 1F), -130.3 (dddt, *J* = 255.9, 16.8, 15.0, 4.8 Hz, 1F).

HRMS (ESI): calcd for $C_{15}H_{19}F_4NO_3$ (M+H) 338.1374, found 338.1373.

3,3,4,4-Tetrafluoro-1-methyl-2-[4-(methylthio)phenyl]piperidine (3f).

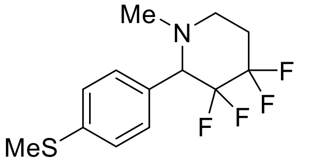

3f

Yield 120 mg (81 %). Colorless crystals. Mp 109–110 °C.

Chromatography: hexanes/EtOAc, from 4/1 to 3/1. R_f 0.34 (hexanes/EtOAc, 3/1).

^{1}H NMR (300 MHz, $CDCl_3$) δ: 7.36 (d, *J* = 8.1 Hz, 2H), 7.27 (d, *J* = 8.1 Hz, 2H), 3.36 (dt, *J* = 21.5, 4.3 Hz, 1H), 3.08–3.00 (m, 1H), 2.57–2.20 (m, 3H), 2.51 (s, 3H), 2.10 (s, 3H).

^{13}C{^{1}H} NMR (75 MHz, $CDCl_3$) δ: 139.7, 130.6 (d, *J* = 1.6 Hz), 129.5, 126.2, 115.8 (dddd, *J* = 260.7, 244.0, 32.5, 21.6 Hz), 113.4 (dddd, *J* = 254.4, 252.2, 30.4, 20.6 Hz), 70.5 (dddd, *J* = 25.0, 18.9, 3.1, 1.2 Hz), 51.2 (d, *J* = 10.5 Hz), 43.1 (t, *J* = 1.3 Hz), 31.2 (dddd, *J* = 20.9, 20.3, 3.5, 1.2 Hz), 15.5.

^{19}F NMR (282 MHz, $CDCl_3$) δ: -119.3 (dm, *J* = 249.1 Hz, 1F), -122.1 (dm, *J* = 249.1 Hz, 1F), -126.2 (dddd, *J* = 256.6, 21.6, 16.6, 3.2 Hz, 1F), -130.1 (dddt, *J* = 256.6, 15.8, 14.9, 4.6 Hz, 1F).

HRMS (ESI): calcd for $C_{13}H_{16}F_4NS$ (M+H) 294.0934, found 294.0928.

3,3,4,4-Tetrafluoro-2-(2-methoxyphenyl)-1-methylpiperidine (3g).

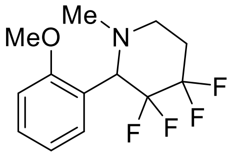

3g

Yield 83 mg (60 %). Colorless crystals. Mp 91–92 °C.

Chromatography: hexanes/EtOAc, 3/1. R_f 0.27 (hexanes/EtOAc, 3/1).

^{1}H NMR (300 MHz, $CDCl_3$) δ: 7.61 (ddd, J = 7.7, 3.1, 1.8 Hz, 1H), 7.35 (ddd, J = 8.3, 7.6, 1.8 Hz, 1H), 7.04 (ddd, J = 7.7, 7.6, 1.1 Hz, 1H), 6.96 (dd, J = 8.3, 1.1 Hz, 1H), 4.22 (d, J = 22.9 Hz, 1H), 3.85 (s, 3H), 3.11–3.01 (m, 1H), 2.68–2.20 (m, 3H), 2.09 (s, 3H).

^{13}C{^{1}H} NMR (75 MHz, $CDCl_3$) δ: 158.7, 130.9 (d, J = 4.2 Hz), 129.7, 121.2, 120.8, 116.0 (dddd, J = 260.3, 244.6, 32.9, 21.4 Hz), 114.0 (dddd, J = 255.8, 251.7, 29.8, 20.1 Hz), 111.2, 61.2 (ddd, J = 25.6, 19.0, 3.4 Hz), 55.9, 51.6 (d, J = 10.5 Hz), 42.8 (t, J = 1.4 Hz), 31.5 (tdd, J = 21.2, 20.2, 3.3, 1.2 Hz).

^{19}F NMR (282 MHz, $CDCl_3$) δ: -119.1 (dm, J = 247.4 Hz, 1F), -122.3 (dm, J = 247.4 Hz, 1F), -125.9 (dm, J = 256.0 Hz), -131.6 (dddd, J = 256.0, 16.7, 13.5, 7.3 Hz).

HRMS (ESI): calcd for $C_{13}H_{16}F_4NO$ (M+H) 278.1163, found 278.1161.

Methyl 4-(3,3,4,4-tetrafluoro-1-methylpiperidin-2-yl)benzoate (3h).

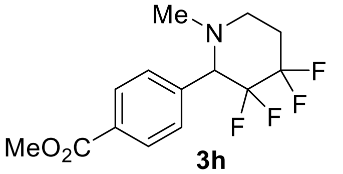

3h

Yield 70 mg (46 %). Colorless crystals. Mp 101–102 °C.

Chromatography: hexanes/EtOAc, 2/1. R_f 0.26 (hexanes/EtOAc, 2/1).

^{1}H NMR (300 MHz, $CDCl_3$) δ: 8.07 (d, J = 8.2 Hz, 2H), 7.53 (dd, J = 8.2, 1.6 Hz, 2H), 3.93 (s, 3H), 3.46 (dt, J = 21.2, 4.5 Hz, 1H), 3.11–2.99 (m, 1H), 2.60–2.18 (m, 3H), 2.08 (s, 3H).

^{13}C{^{1}H} NMR (75 MHz, $CDCl_3$) δ: 166.76, 137.90, 130.89, 130.31 (d, J = 1.7 Hz), 129.57, 115.63 (dddd, J = 260.6, 244.3, 32.2, 21.3 Hz), 113.11 (dddd, J = 255.2, 252.8, 30.5, 20.8 Hz), 70.70 (dddd, J = 25.0, 19.0, 3.3, 1.3 Hz), 52.20, 51.16 (d, J = 10.5 Hz), 43.10 (t, J = 1.4 Hz), 31.11 (dddd, J = 21.2, 20.0, 3.3, 1.2 Hz).

^{19}F NMR (282 MHz, $CDCl_3$) δ: -119.4 (dm, J = 249.6 Hz, 1F), -122.4 (dm, J = 249.6 Hz, 1F), -125.9 (dm, J = 257.5 Hz, 1F), -130.04 (dddt, J = 257.5, 18.0, 14.4, 4.9 Hz).

HRMS (ESI): calcd for $C_{14}H_{16}F_4NO_2$ (M+H) 306.1112, found 306.1120.

3,3,4,4-Tetrafluoro-1-methyl-2-[4-(trifluoromethyl)phenyl]piperidine (3i).

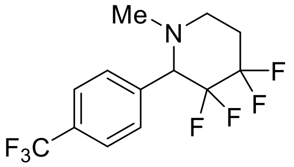

3i

Yield 124 mg (78 %). Colorless crystals. Mp 69–70 °C.

Chromatography: hexanes/EtOAc, 4/1. R_f 0.25 (hexanes/EtOAc, 4/1).

^{1}H NMR (300 MHz, $CDCl_3$) δ: 7.68 (d, *J* = 8.2 Hz, 2H), 7.59 (d, *J* = 8.2 Hz, 2H), 3.49 (dt, *J* = 21.0, 4.4 Hz, 1H), 3.08 (d, *J* = 11.1 Hz, 1H), 2.68–2.21 (m, 3H), 2.11 (s, 3H).

^{13}C{^{1}H} NMR (75 MHz, $CDCl_3$) δ: 137.1, 131.4 (q, *J* = 32.4 Hz), 130.8 (d, *J* = 1.7 Hz), 125.4 (q, *J* = 3.8 Hz), 122.2 (q, *J* = 271.8 Hz), 115.7 (dddd, *J* = 260.8, 244.2, 32.2, 21.3 Hz), 113.2 (dddd, *J* = 255.0, 253.0, 30.5, 20.8 Hz), 70.7 (dddd, *J* = 24.9, 19.1, 3.4, 1.3 Hz), 51.2 (d, *J* = 10.4 Hz), 43.2 (t, *J* = 1.4 Hz), 31.2 (dddd, *J* = 21.1, 20.2, 3.3, 1.1 Hz).

^{19}F NMR (282 MHz, $CDCl_3$) δ: -62.5, -119.5 (dm, *J* = 250.4 Hz, 1F), -122.42 (dm, *J* = 250.4 Hz, 1F), -125.90 (ddtd, *J* = 257.7, 20.9, 16.6, 3.9 Hz, 1F), -130.02 (dddt, *J* = 257.7, 17.7, 13.3, 4.7 Hz, 1F).

HRMS (ESI): calcd for $C_{13}H_{13}F_7N$ (M+H) 316.0931, found 316.0923.

4-(3,3,4,4-Tetrafluoro-1-methylpiperidin-2-yl)benzonitrile (3j).

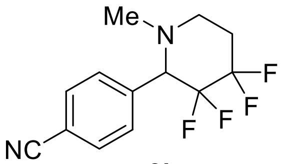

3j

Yield 46 mg (34 %). Colorless crystals. Mp 102–104 °C. Chromatography: hexanes/EtOAc, 2/1. R_f 0.28 (hexanes/EtOAc, 2/1).

^{1}H NMR (300 MHz, $CDCl_3$) δ: 7.68 (d, *J* = 8.4 Hz, 2H), 7.56 (d, *J* = 8.4 Hz, 2H), 3.46 (dt, *J* = 20.8, 4.4 Hz, 1H), 3.09–3.00 (m, 1H), 2.60–2.18 (m, 3H), 2.06 (s, 3H).

^{13}C{^{1}H} NMR (75 MHz, $CDCl_3$) δ: 138.3, 132.2, 131.1 (d, *J* = 1.7 Hz), 118.5, 113.1, 115.5 (dddd, *J* = 260.8, 243.7, 32.1, 21.2 Hz), 113.1 (dddd, *J* = 255.9, 253.4, 30.7, 20.8 Hz), 70.6 (dddd, *J* = 24.9, 19.0, 3.4, 1.3 Hz), 51.1 (d, *J* = 10.5 Hz), 43.2 (t, *J* = 1.3 Hz), 31.1 (dddd, *J* = 21.1, 20.1, 3.3, 1.3 Hz).

^{19}F NMR (282 MHz, $CDCl_3$) δ: -119.5 (dm, *J* = 249.9 Hz, 1F), -122.5 (dm, *J* = 249.9 Hz, 1F), -125.7 (ddd, *J* = 257.1, 21.5,16.2, 4.6 Hz, 1F), -129.9 (ddddd, *J* = 257.8, 17.0, 13.9, 5.8, 4.4 Hz, 1F).

HRMS (ESI): calcd for $C_{13}H_{13}F_4N_2$ (M+H) 273.1009, found 273.1008.

2-(3,3,4,4-Tetrafluoro-1-methylpiperidin-2-yl)pyridine (3k).

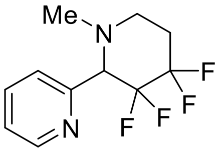

3k

Yield 92 mg (74 %). Colorless oil.

Chromatography: hexanes/EtOAc, from 1/1 to 1/2. R_f 0.22 (hexanes/EtOAc, 1/1).

^{1}H NMR (300 MHz, $CDCl_3$) δ: 8.62 (ddd, *J* = 4.9, 1.8, 1.0 Hz, 1H), 7.72 (ddd, *J* = 7.8, 6.8, 1.8 Hz, 1H), 7.52 (ddd, *J* = 7.8, 1.2 , 1.0 Hz, 1H), 7.28 (ddd, *J* = 6.8, 4.9, 1.2 Hz, 1H), 3.68 (dt, *J* = 21.8, 4.7 Hz, 1H), 3.07–2.99 (m, 1H), 2.61–2.16 (m, 3H), 2.08 (s, 3H).

^{13}C{^{1}H} NMR (75 MHz, $CDCl_3$) δ: 153.3, 149.4, 136.5, 125.0 (d, *J* = 3.2 Hz), 123.8, 115.5 (dddd, *J* = 260.2, 244.1, 31.9, 21.0 Hz), 113.5 (dddd, *J* = 256.8, 252.9, 30.7, 20.7 Hz), 72.4 (dddd, *J* = 24.2, 18.1, 2.7, 1.0 Hz), 51.2 (d, *J* = 10.5 Hz), 43.0 (t, *J* = 1.3 Hz), 31.2 (tdd, *J* = 21.0, 3.3, 1.0 Hz).

^{19}F NMR (282 MHz, $CDCl_3$) δ: -119.5 (dm, *J* = 248.7 Hz, 1F), -122.9 (dm, *J* = 248.7 Hz, 1F), -125.7 (dm, *J* = 256.6 Hz, 1F), -131.0 (dm, *J* = 256.6 Hz, 1F).

HRMS (ESI): calcd for $C_{11}H_{13}F_4N_2$ (M+H) 249.1009, found 249.1010.

1,1,2,2-Tetrafluoro-2,3,4,6,7,11b-hexahydro-1*H*-pyrido[2,1-*a*]isoquinoline (3l).

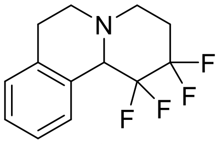

3l

Yield 87 mg (67 %). Colorless oil.

Chromatography: hexanes/EtOAc, 3/1. R_f 0.26 (hexanes/EtOAc, 3/1).

^{1}H NMR (300 MHz, $CDCl_3$) δ: 7.55 (d, *J* = 7.0 Hz, 1H), 7.33–7.17 (m, 3H), 4.08 (d, *J* =23.7 Hz, 1H), 3.26–2.96 (m, 4H), 2.91–2.70 (m, 2H), 2.58–2.17 (m, 2H).

^{13}C{^{1}H} NMR (75 MHz, $CDCl_3$) δ: 136.2, 129.3, 128.6, 128.1 (d, *J* = 11.0 Hz), 127.8, 126.0 (d, *J* = 1.5 Hz), 116.4 (dddd, *J* = 261.3, 244.5, 31.4, 21.8 Hz), 115.2 (dddd, *J* = 262.2, 257.0, 29.9, 20.7 Hz), 62.0 (dddd, *J* = 20.9, 19.6, 2.5, 1.2 Hz), 50.1 (d, *J* = 9.8 Hz), 48.4 (d, *J* = 1.9 Hz), 31.0 (ddd, *J* = 21.8, 19.0, 2.6, 1.3 Hz), 29.8.

^{19}F NMR (282 MHz, $CDCl_3$) δ: -116.6 (ddqd, *J* = 251.6, 34.9, 14.2, 4.1 Hz, 1F), -119.6 (dt, *J* = 251.6, 13.6 Hz, 1F), -124.0 (ddt, *J* =255.3, 23.8, 15.0 Hz, 1F), -128.4 (d, *J* =255.3 Hz, 1F).

HRMS (ESI): calcd for $C_{13}H_{14}F_4N$ (M+H) 260.1057, found 260.1064.

1-Benzyl-3,3,4,4-tetrafluoro-2-methylpiperidine (3m).

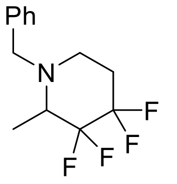

3m

Yield 90 mg (68 %). Colorless oil.

Chromatography: hexanes/EtOAc, 20/1. R_f 0.30 (hexanes/EtOAc, 20/1).

^{1}H NMR (300 MHz, $CDCl_3$) δ: 7.44–7.30 (m, 5H), 4.00 (d, *J* = 13.6 Hz, 1H), 3.44 (d, *J* = 13.6 Hz, 1H), 3.13 –2.97 (m, 1H), 2.93–2.84 (m, 1H), 2.51–2.40 (m, 1H), 2.24–2.08 (m, 2H), 1.39 (d, *J*= 6.6 Hz, 3H).

^{13}C{^{1}H} NMR (75 MHz, $CDCl_3$) δ: 138.4, 128.7, 128.6, 127.5, 116.2 (tt, *J* = 252.4, 26.7 Hz), 115.0 (tt, *J* = 254.6, 25.4 Hz), 58.8 (t, *J*= 22.7 Hz), 56.0, 45.5 (t, *J* = 5.2 Hz), 31.2 (t, *J* = 20.6 Hz), 9.8.

^{19}F NMR (282 MHz, $CDCl_3$) δ: -118.9 (d, *J* = 255.1 Hz, 1F), -120.0 (d, *J* = 255.1 Hz, 1F), -128.1 (br s, 1F), -131.1 (dd, *J* = 252.8, 14.0 Hz, 1F).

HRMS (ESI): calcd for $C_{13}H_{16}F_4N$ (M+H) 262.1213, found 262.1213.

1-Benzyl-3,3,4,4-tetrafluoro-2-propylpiperidine (3n).

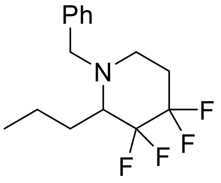

3n

Yield 87 mg (60 %). Colorless oil.

Chromatography: hexanes/EtOAc, 40/1. R_f 0.26 (hexanes/EtOAc, 40/1).

^{1}H NMR (300 MHz, $CDCl_3$) δ: 7.41–7.28 (m, 5H), 3.86 (d, *J* = 14.0 Hz, 1H), 3.78 (d, *J* = 14.0 Hz, 1H), 3.20–3.06 (m, 1H), 2.97 (dt, *J* = 14.1, 5.1 Hz, 1H), 2.69 (ddd, *J* = 13.1, 8.5, 4.2 Hz, 1H), 2.29–2.10 (m, 2H), 1.83–1.41 (m, 4H), 1.00 (t, *J* = 7.2 Hz, 3H).

^{13}C{^{1}H} NMR (75 MHz, $CDCl_3$) δ: 139.1 (t, *J* = 1.2 Hz), 128.6, 128.5, 127.4, 116.5 (tt, *J* = 252.1, 26.9 Hz), 116.0 (tt, *J* = 258.9, 25.4 Hz), 62.6 (t, *J* = 21.2 Hz), 53.4, 43.6 (t, *J* = 4.3 Hz), 29.0 (t, *J* = 20.4 Hz), 26.4 (ddd, *J* = 3.8, 2.5, 1.4 Hz), 19.9, 14.1.

^{19}F NMR (282 MHz, $CDCl_3$) δ: -117.1 (s, 2F), -123.4 (br s, 1F), -127.1 (d, *J* = 253.4 Hz, 1F).

HRMS (ESI): calcd for $C_{15}H_{20}F_4N$ (M+H) 290.1526, found 290.1529.

1-Benzyl-3,3,4,4-tetrafluoro-2-isobutylpiperidine (3o).

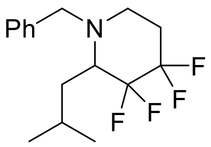

3o

Yield 92 mg (60 %). Colorless oil.

Chromatography: hexanes/EtOAc, 40/1. R_f 0.32 (hexanes/EtOAc, 40/1).

^{1}H NMR (300 MHz, $CDCl_3$) δ: 7.30–7.17 (m, 5H), 3.73 (s, 2H), 3.12 (q, *J* = 11.3 Hz, 1H), 2.87 (dt, *J* = 14.3, 5.3 Hz, 1H), 2.62 (ddd, *J* = 14.4, 8.6, 4.2 Hz, 1H), 2.19–2.01 (m, 2H), 1.81-1.60 (m, 2H), 1.42 (ddd, *J* = 14.8, 9.5, 3.8 Hz, 1H), 0.89 (d, *J* =6.6 Hz, 3H), 0.84 (d, *J* = 6.5 Hz, 3H).

^{13}C{^{1}H} NMR (75 MHz, $CDCl_3$) δ: 139.1, 128.6, 128.5, 127.5, 116.5 (tt, *J* = 252.2, 27.1 Hz), 116.1 (tt, *J* = 259.7, 25.4 Hz), 60.9 (t, *J* = 21.1 Hz), 53.5, 43.4 (t, *J* = 4.0 Hz), 33.1 (ddd, *J* = 3.6, 2.2, 1.5 Hz), 28.8 (t, *J* = 20.4 Hz), 25.0, 23.7, 21.4.

^{19}F NMR (282 MHz, $CDCl_3$) δ: -117.6 (s, 2F), -124.0 (br s, 1F), -128.0 (d, *J* = 254.5 Hz, 1F).

HRMS (ESI): calcd for $C_{16}H_{22}F_4N$ (M+H) 304.1683, found 304.1676.

3,3,4,4-Tetrafluoro-1-methyl-2-pentylpiperidine (3p).

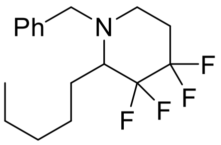

3p

Yield 87 mg (55 %). Colorless oil.

Chromatography: hexanes/EtOAc, 40/1. R_f 0.35 (hexanes/EtOAc, 40/1).

^{1}H NMR (300 MHz, $CDCl_3$) δ: 7.41-7.27 (m, 5H), 3.81 (q, *J* = 11.5 Hz, 2H), 3.17-3.02 (m, 1H), 3.01-2.92 (m, 1H), 2.73-2.62 (m, 1H), 2.29-2.10 (m, 2H), 1.79 (q, *J* = 7.4 Hz, 2H), 1.67-1.29 (m, 6H), 0.95 (t, *J* = 6.8 Hz, 3H).

^{13}C {^{1}H} NMR (75 MHz, $CDCl_3$) δ: 139.0, 128.62, 128.56, 127.5, 116.5 (tt, *J* = 252.4, 26.8 Hz), 116.1 (tt, *J* = 259.1, 25.5 Hz), 62.9 (t, *J* = 21.2 Hz), 53.9, 43.7 (t, *J* = 4.5 Hz), 31.8, 29.1 (t, *J* = 20.5 Hz), 26.3, 24.2 (ddd, *J* = 3.9, 2.6, 1.4 Hz), 22.6, 14.1.

^{19}F NMR (282 MHz, $CDCl_3$) δ: -117.9 (s, 2F), -112.4 (br. s, 1F), -127.9 (d, *J* = 250.5 Hz, 1F).

HRMS (ESI): calcd for $C_{17}H_{24}F_4N$ (M+H) 318.1839, found 318.1842.

3,3,4,4-Tetrafluoro-1-methyl-2-(2-phenylethyl)piperidine (3q).

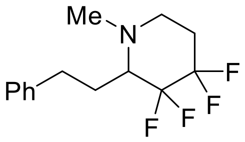

3q

Yield 94 mg (68 %). Colorless oil.

Chromatography: hexanes/EtOAc, 5/1. R_f 0.30 (hexanes/EtOAc, 5/1).

^{1}H NMR (300 MHz, $CDCl_3$) δ: 7.40-7.23 (m, 5H), 3.07-2.68 (m, 5H), 2.48 (s, 3H), 2.41-1.98 (m, 4H).

^{13}C{^{1}H} NMR (75 MHz, $CDCl_3$) δ: 141.6, 128.6, 128.5, 126.2, 116.0 (tt, *J* = 252.1, 26.7 Hz), 115.8 (tdd, *J* =257.6, 28.4, 22.8 Hz), 62.8 (t, *J* = 21.3 Hz), 49.6 (d, *J* = 9.0 Hz), 39.3, 32.4 (t, *J* = 1.2 Hz), 29.8 (t, *J* = 20.5 Hz), 26.6 (q, *J* = 1.8 Hz).

^{19}F NMR (282 MHz, $CDCl_3$) δ: -118.4 (dd, *J* = 250.7, 23.5 Hz, 1F), 120.4 (d, *J* = 250.7 Hz), -126.2 (d, *J* = 254.5 Hz), -128.3 (br s).

HRMS (ESI): calcd for $C_{14}H_{18}F_4N$ (M+H) 276.1370, found 276.1374.

X-ray data

X-ray diffraction data were collected at 100 K on a Bruker Quest D8 diffractometer equipped with a Photon-III area-detector (graphite monochromator, shutterless φ- and ω-scan technique), using MoKα irradiation. The intensity data were integrated by the SAINT program[15] and corrected for absorption and decay using SADABS.[16] The structure was solved by direct methods using SHELXT[17] and refined on F^2 using SHELXL-2018.[18] All non-hydrogen atoms were refined with anisotropic displacement parameters. Hydrogen atoms were placed in ideal calculated positions and refined as riding atoms with relative isotropic displacement parameters. The tetrafluoropiperidinyl fragment is disordered over two positions with the site occupancy ratio of 0.9674(7):0.0326(7). The SHELXTL program suite[1] was used for molecular graphics. The CIF files were deposited with the Cambridge Crystallographic Data Centre (**3f**: 2032382; **3h**: 2032383).

Table S1: Crystal data and structure refinement for **3f,h**.

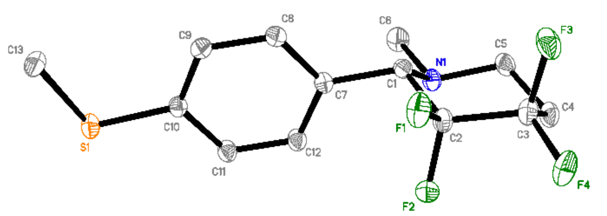

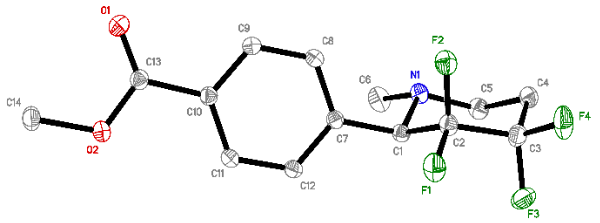

Compound	**3f**		**3h**	
Empirical formula	C13 H15 F4 N S		C14 H15 F4 N O2	
Formula weight	293.32		305.27	
Temperature	100(2) K		100(2) K	
Wavelength	0.71073 Å		0.71073 Å	
Crystal system	Monoclinic		Monoclinic	
Space group	$P2_1/c$		$P2_1/c$	
Unit cell dimensions	a = 13.2686(3) Å	α= 90°.	a = 13.4130(3) Å	α= 90°.
	b = 7.4126(2) Å	β= 93.2710(10)°.	b = 7.3746(2) Å	β= 90.2570(10)°.
	c = 13.3406(3) Å	γ = 90°.	c = 13.8840(3) Å	γ = 90°.
Volume	1309.97(5) $Å^3$		1373.33(6) $Å^3$	
Z	4		4	
Density (calculated)	1.487 g/cm^3		1.476 g/cm^3	
Absorption coefficient	0.280 mm^{-1}		0.134 mm^{-1}	
F(000)	608		632	
Crystal size	0.58 x 0.24 x 0.14 mm^3		0.59 x 0.56 x 0.15 mm^3	
Theta range for data collection	3.059 to 36.325°.		2.934 to 37.060°.	
Index ranges	-22<=h<=21, -12<=k<=12, -22<=l<=22		-22<=h<=22, -12<=k<=12, -23<=l<=23	
Reflections collected	59051		52704	
Independent reflections	6357 [R(int) = 0.0386]		7002 [R(int) = 0.0350]	
Observed reflections	5116		5318	
Completeness to theta = 25.242°	99.80%		99.80%	
Absorption correction	Semi-empirical from equivalents		Semi-empirical from equivalents	
Max. and min. transmission	0.6967 and 0.6461		0.7473 and 0.6996	
Refinement method	Full-matrix least-squares on F^2		Full-matrix least-squares on F^2	
Data / restraints / parameters	6357 / 12 / 205		7002 / 0 / 192	
Goodness-of-fit on F^2	1.023		1.046	
Final R indices [I>2sigma(I)]	R1 = 0.0364, wR2 = 0.0896		R1 = 0.0406, wR2 = 0.1097	
R indices (all data)	R1 = 0.0509, wR2 = 0.0984		R1 = 0.0606, wR2 = 0.1205	
Largest diff. peak and hole	0.778 and -0.392 e.$Å^{-3}$		0.530 and -0.285 e.$Å^{-3}$	

References

1. Tyrrell, E.; Allen, J.; Jones, K.; Beauchet, R. *Synthesis* **2005**, 2393–2399.
2. Dmitriev, I. A.; Supranovich, V. I.; Levin, V. V.; Struchkova, M. I.; Dilman, A. D. *Adv. Synth. Cat.* **2018**, *360*, 3788–3792.
3. Chen, R.; Sun, S.; Wang, G.; Guo, H. A *Tetrahedron Lett.* **2018**, *59*, 1916–1920.
4. Canterbury, D. P.; Herrick, I. R.; Um, J.; Houk, K. N.; Frontier, A. J. *Tetrahedron* **2009**, 65, 3165–3179.
5. Shimizu, T.; Ishizaki, M.; Nitada, N. *Chem. Pharm. Bull.* **2002**, *50*, 908–921.
6. Yoshimura, F.; Abe, T.; Tanino, K. *Synlett* **2014**, *25*, 1863–1868.
7. Honda, K.; Mikami, K. *Chem. Asian J.* **2018**, *13*, 2838–2841.
8. Chan, K. S.; Yeung, M. L.; Chan, W.; Wang, R.-J.; Mak, T. C. W. *J. Org. Chem.* **1995**, *60*, 1741–1747.
9. Murahashi, S.; Mitsui, H.; Shiota, T.; Tsuda, T.; Watanabe, S. *J. Org. Chem.* **1990**, *55*, 1736–1744.
10. Yakura, T.; Nakazawa, M.; Takino, T.; Ikeda, M. *Chem. Pharm. Bull.* **1992**, *40*, 2014–2018.
11. Dondoni, A.; Franco, S.; Junquera, F.; Merchán, F. L.; Merino, P.; Tejero, T. *Synth. Commun.* **1994**, *24*, 2537–2550.
12. Zheng, H.; McDonald, R.; Hall, D. G. *Chem. Eur. J.* **2010**, *16*, 5454–5460.
13. (a) Emmons, W. D. *J. Am. Chem. Soc.* **1957**, *79*, 5739–5754. (b) Yijima, C.; Tsujimoto, T.; Suda, K.; Yamauchi, M. *Bull. Chem. Soc. Jpn.* **1986**, *59*, 2165–2170. (c) Colonna, S.; Pironti, V.; Carrea, G.; Pasta, P.; Zambianchi, F. *Tetrahedron* **2004**, *60*, 569–575.
14. (a) Bartoli, G.; Marcantoni, E.; Petrini, M. *J. Org. Chem.* **1992**, *57*, 5834–5840.
15. Bruker. APEX-III. *Bruker AXS Inc.*, Madison, Wisconsin, USA, **2019**.
16. Krause, L.; Herbst-Irmer, R.; Sheldrick, G. M.; Stalke, D. *J. Appl. Cryst.* **2015**, 48, 3–10.
17. Sheldrick, G. M. *Acta Cryst.* **2015**, A71, 3–8.
18. Sheldrick, G. M. *Acta Cryst.* **2015**, C71, 3–8.

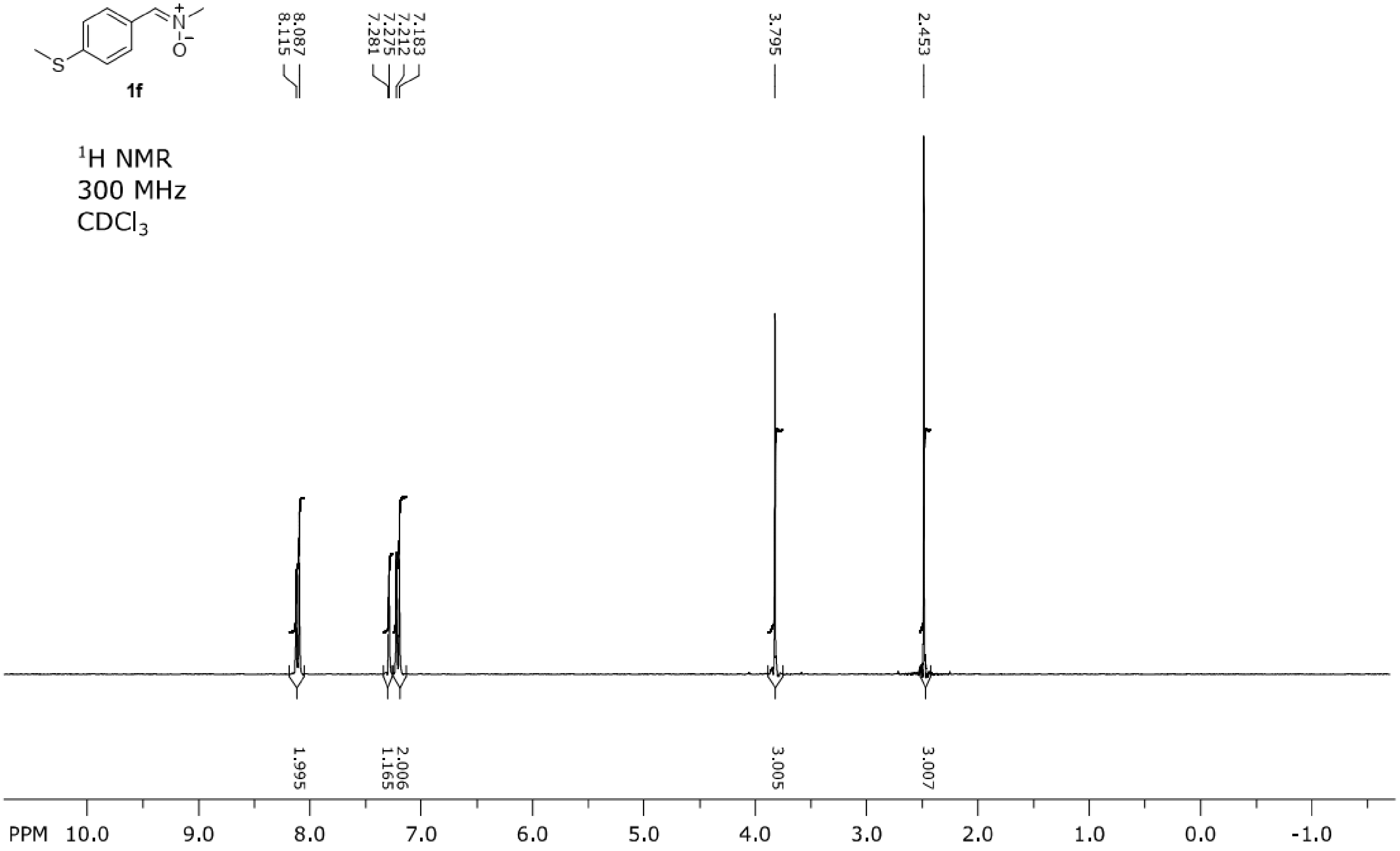
1f
1H NMR
300 MHz
CDCl3
8.087
8.115
7.183
7.212
7.275
7.281
3.795
2.453
1.995
2.006
1.165
3.005
3.007
PPM 10.0 9.0 8.0 7.0 6.0 5.0 4.0 3.0 2.0 1.0 0.0 -1.0

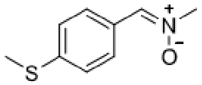

1f

$^{13}C\{^{1}H\}$ NMR
75 MHz
$CDCl_3$

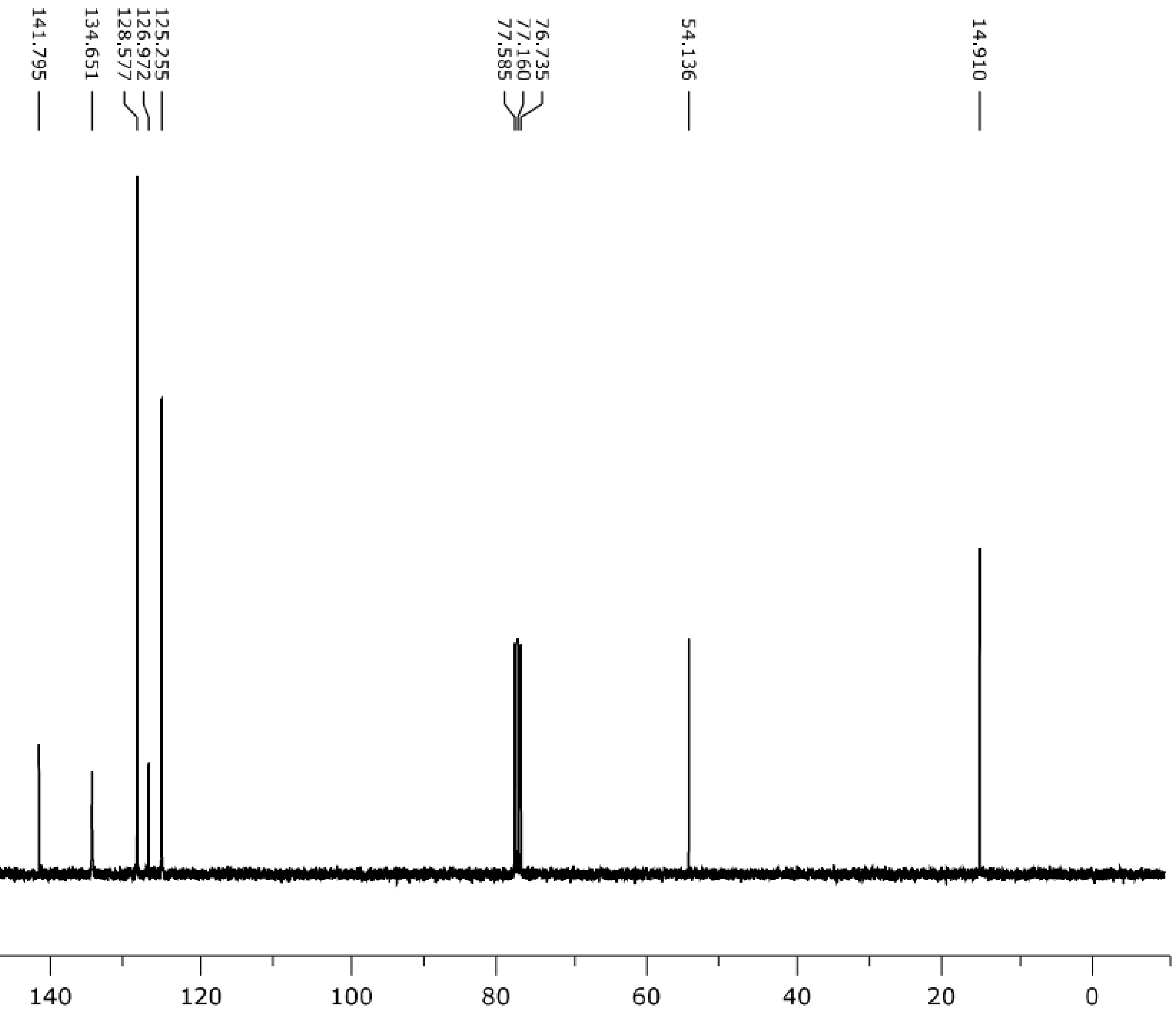

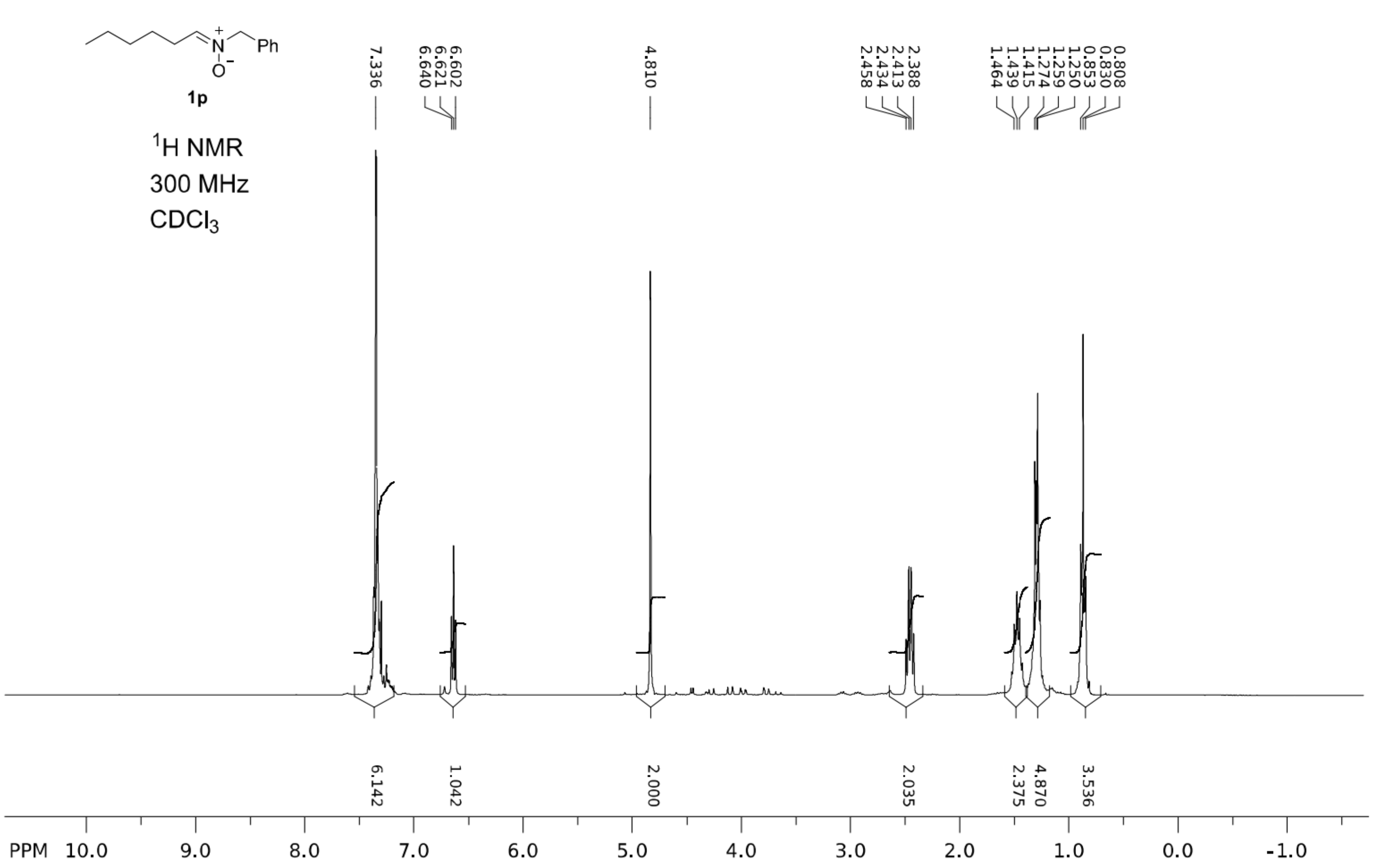

Ph
1p
1H NMR
300 MHz
CDCl3
7.336
6.640
6.621
6.602
4.810
2.458
2.434
2.413
2.388
1.464
1.439
1.415
1.274
1.259
1.250
0.853
0.830
0.808
6.142
1.042
2.000
2.035
2.375
4.870
3.536
PPM 10.0 9.0 8.0 7.0 6.0 5.0 4.0 3.0 2.0 1.0 0.0 -1.0

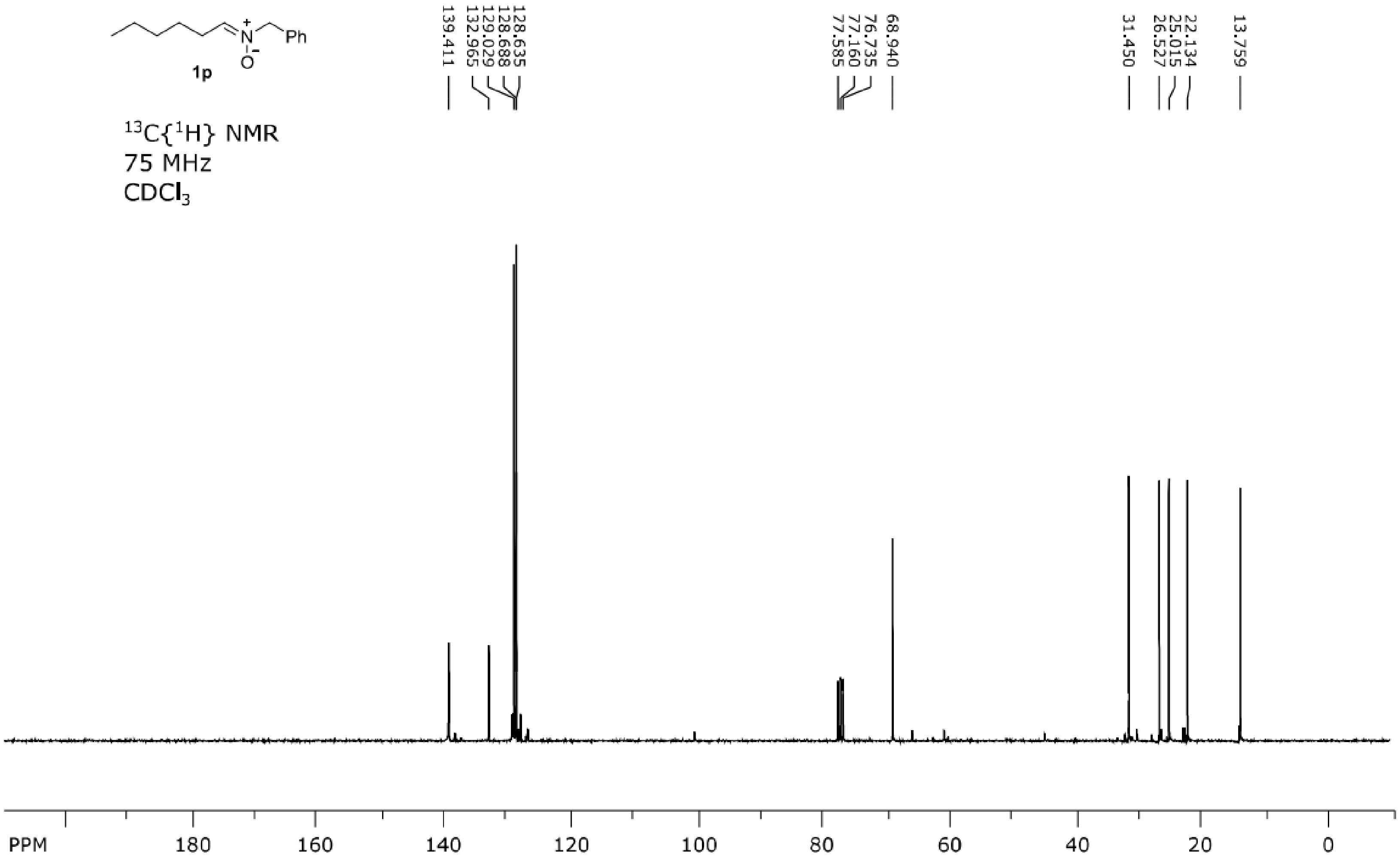

1p
$^{13}C\{^{1}H\}$ NMR
75 MHz
$CDCl_3$
139.411
132.965
129.029
128.688
128.635
77.585
77.160
76.735
68.940
31.450
26.527
25.015
22.134
13.759
PPM
180
160
140
120
100
80
60
40
20
0

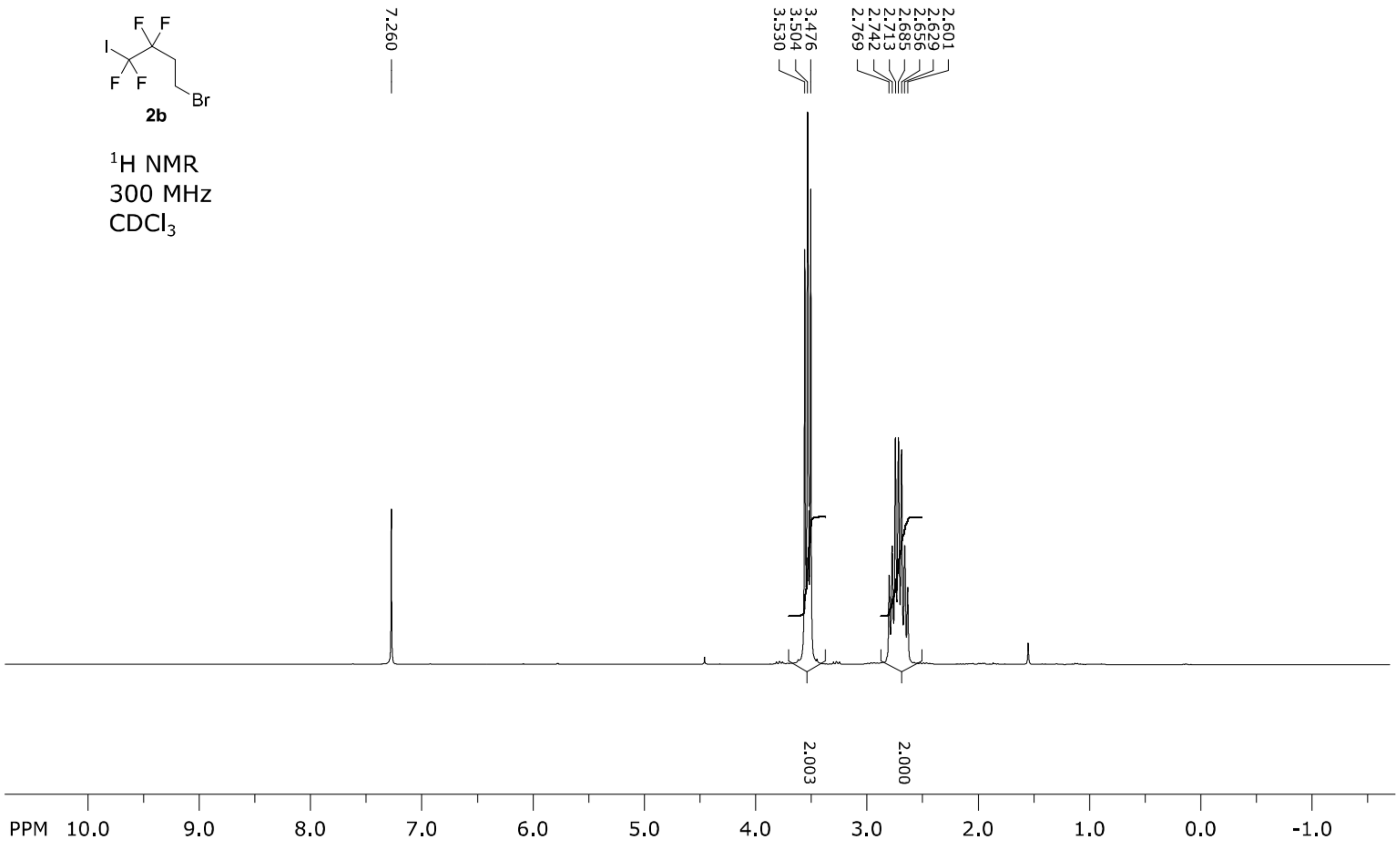
2b
1H NMR
300 MHz
CDCl3
7.260
3.530
3.504
3.476
2.769
2.742
2.713
2.685
2.656
2.629
2.601
2.003
2.000
PPM 10.0 9.0 8.0 7.0 6.0 5.0 4.0 3.0 2.0 1.0 0.0 -1.0

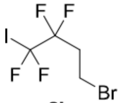

2b

$^{13}C\{^{1}H\}$ NMR
75 MHz
$CDCl_3$

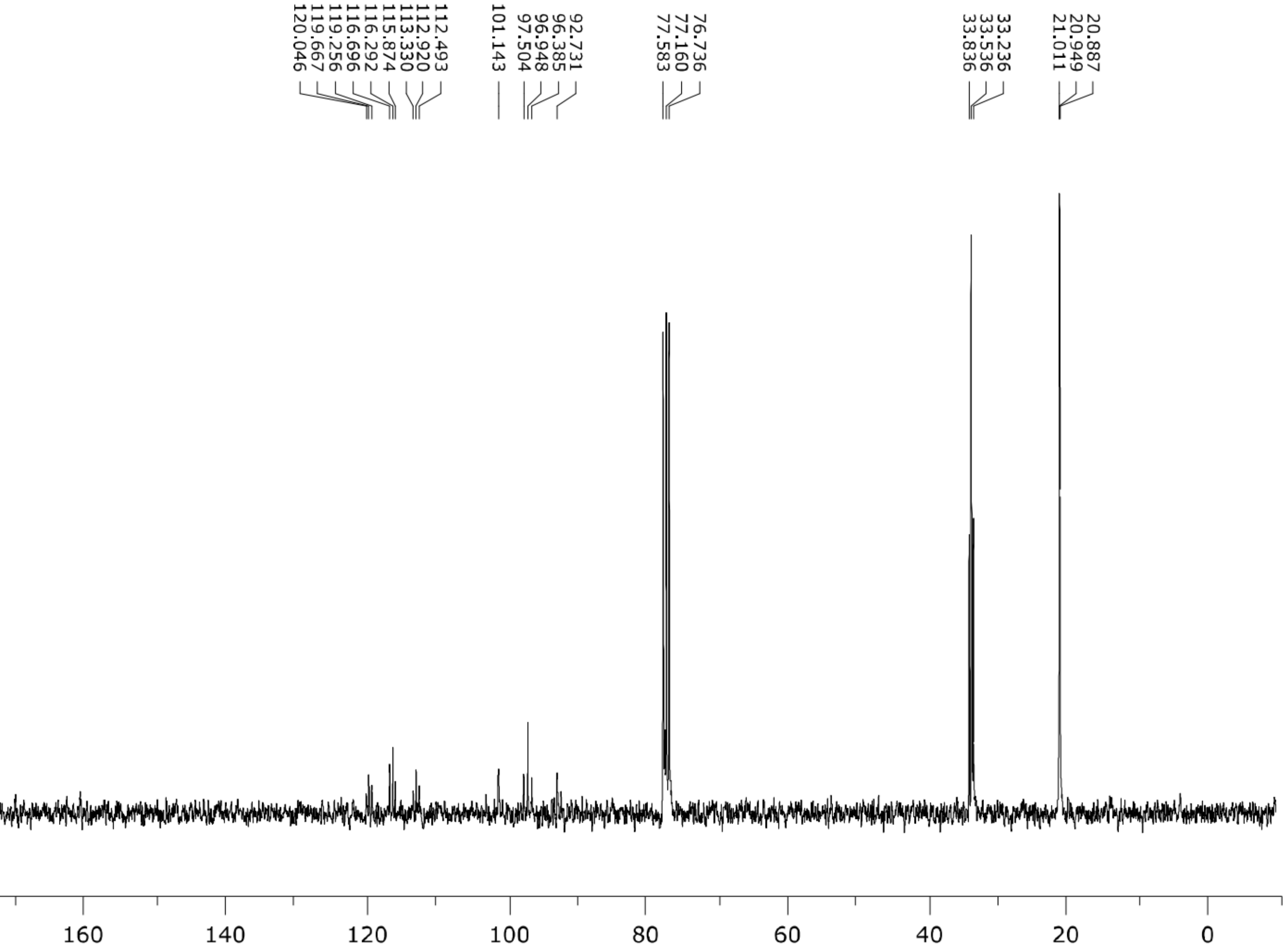

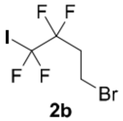

2b

^{19}F NMR
282 MHz
$CDCl_3$

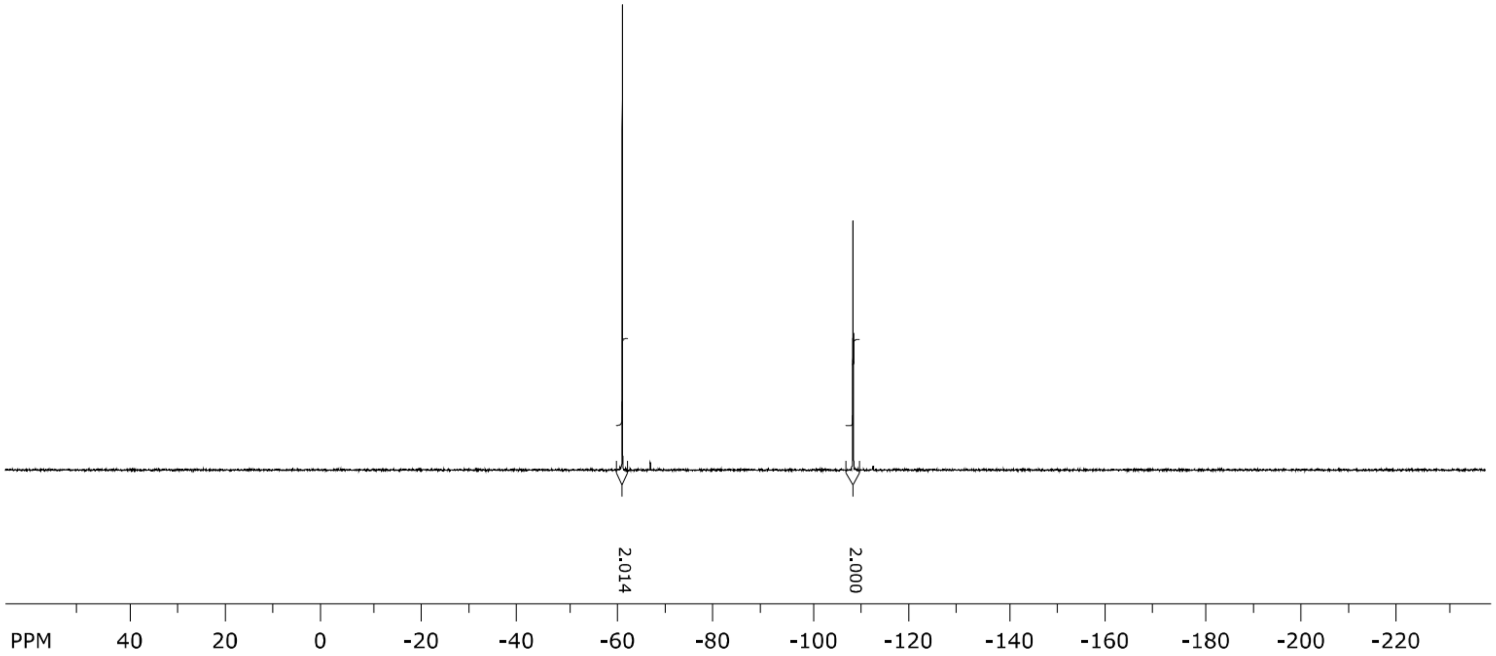

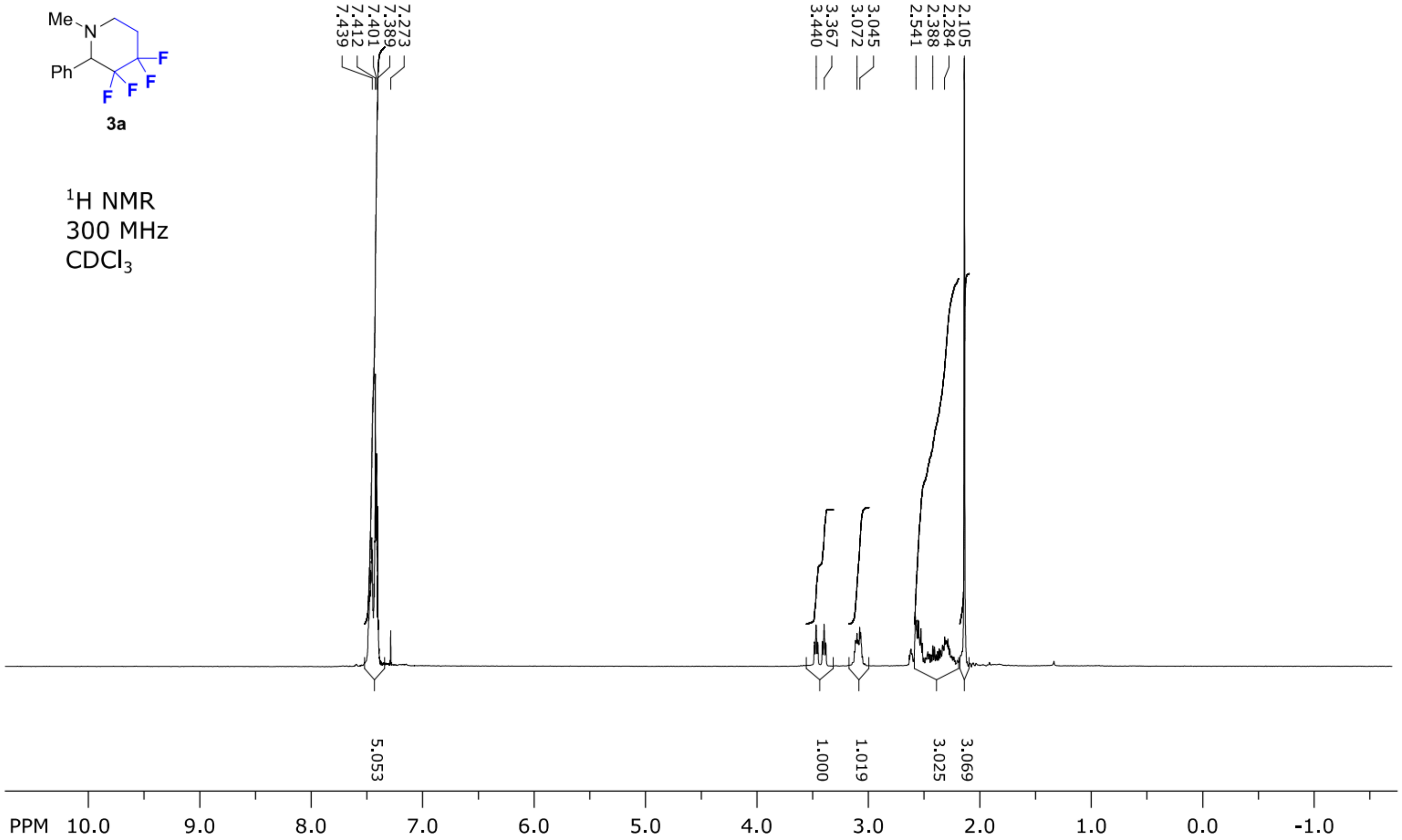

Me
N
Ph
F
F
F
F
3a
^{1}H NMR
300 MHz
$CDCl_3$
7.439
7.412
7.401
7.389
7.273
3.440
3.367
3.072
3.045
2.541
2.388
2.284
2.105
5.053
1.000
1.019
3.025
3.069
PPM 10.0 9.0 8.0 7.0 6.0 5.0 4.0 3.0 2.0 1.0 0.0 -1.0

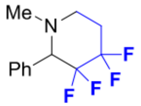

3a

$^{13}C\{^{1}H\}$ NMR
75 MHz
$CDCl_3$

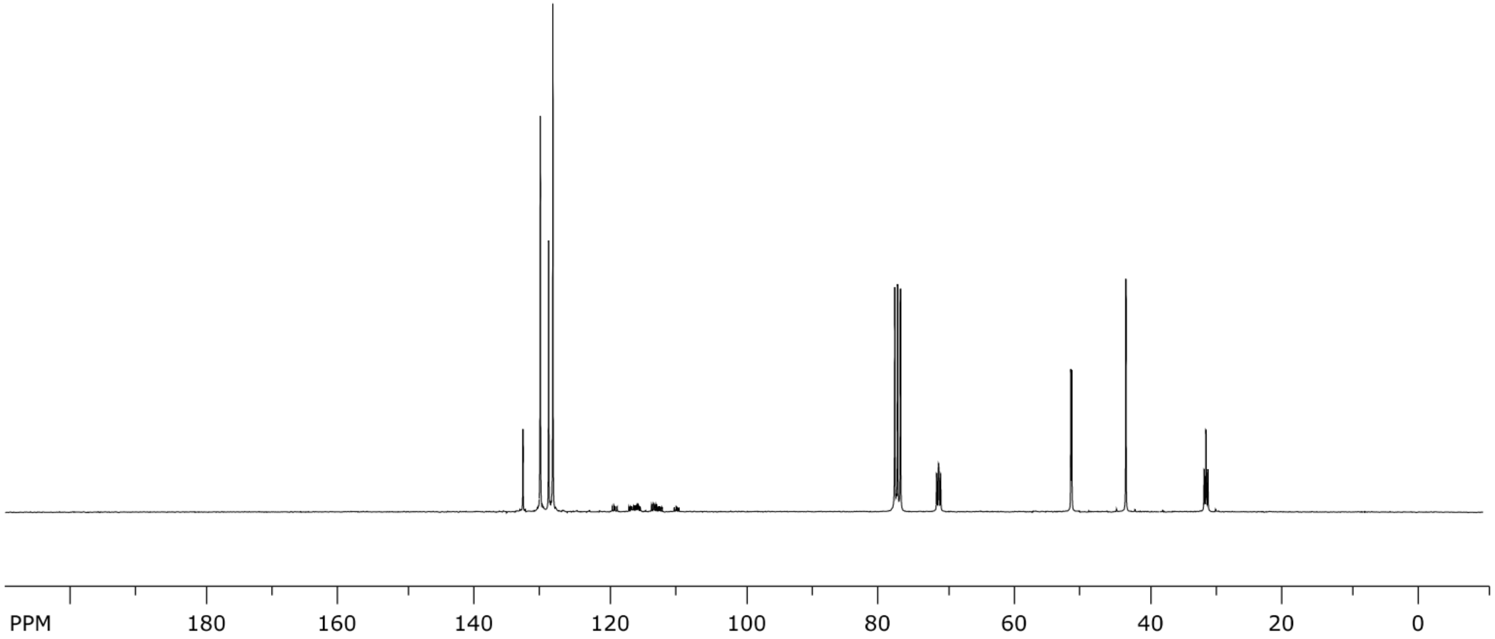

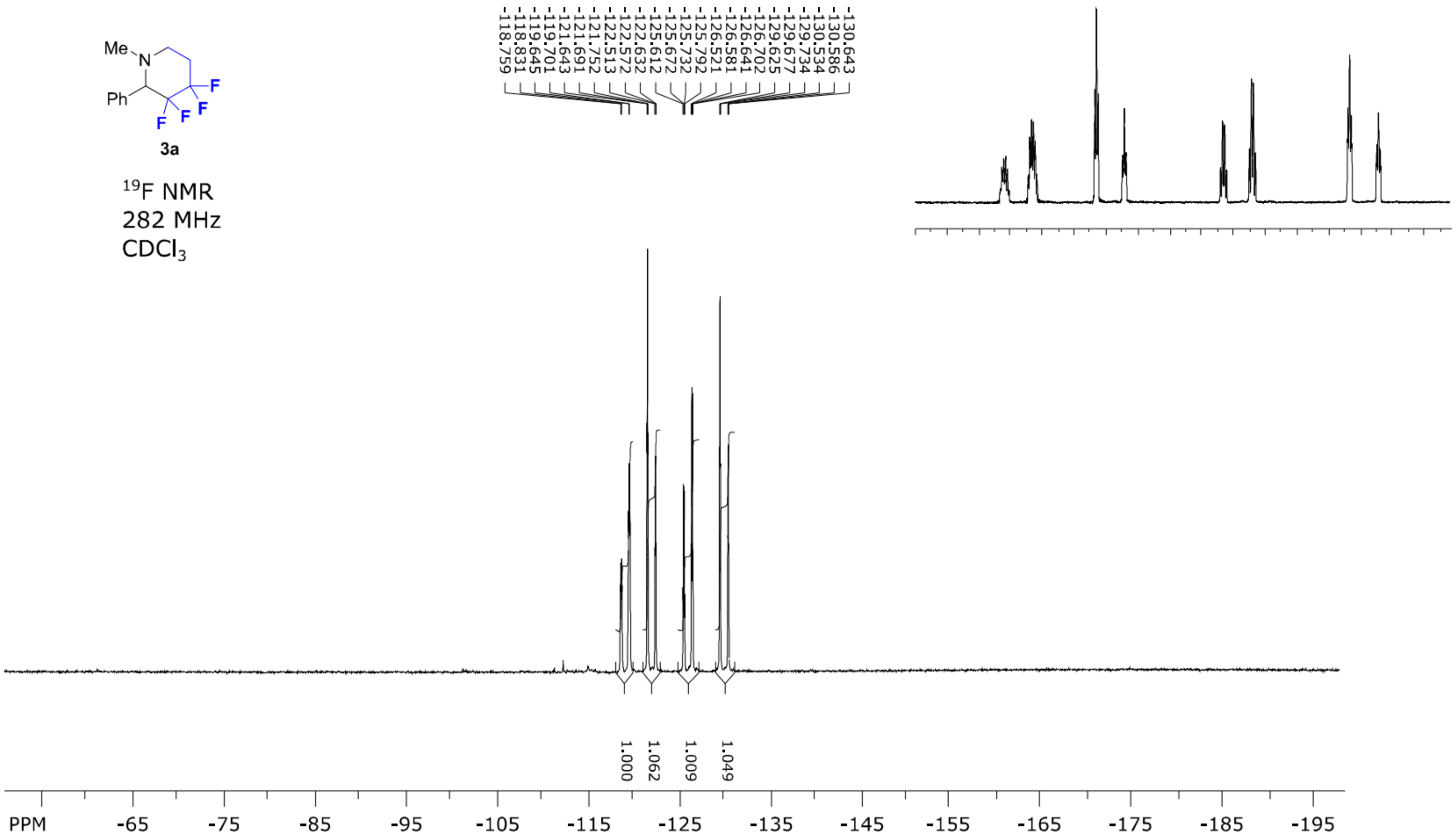
Me
N
Ph
F
F
F
F
3a
^{19}F NMR
282 MHz
$CDCl_3$
-118.759
-118.831
-119.645
-119.701
-121.643
-121.691
-121.752
-122.513
-122.572
-122.632
-125.612
-125.672
-125.732
-125.792
-126.521
-126.581
-126.641
-126.702
-129.625
-129.677
-129.734
-130.534
-130.586
-130.643
1.000
1.062
1.009
1.049
PPM
-65
-75
-85
-95
-105
-115
-125
-135
-145
-155
-165
-175
-185
-195

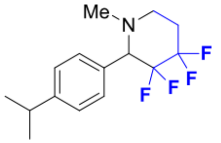

3b

^{1}H NMR
300 MHz
$CDCl_3$

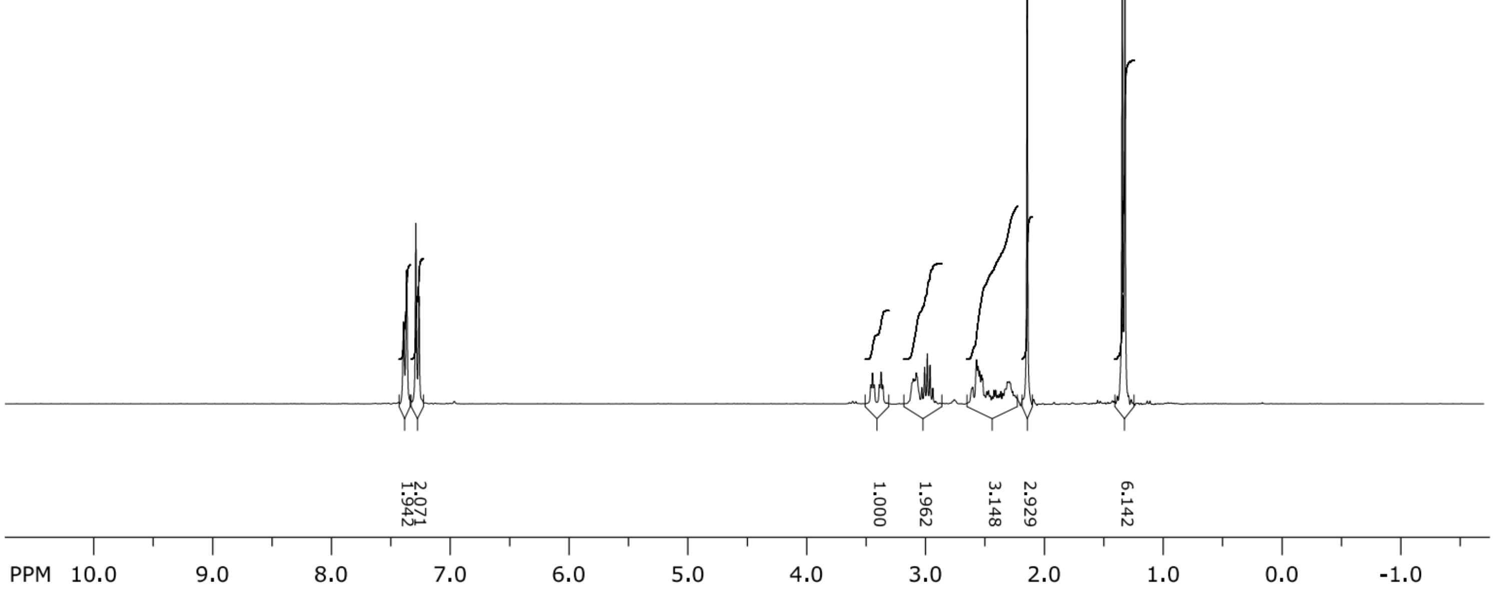

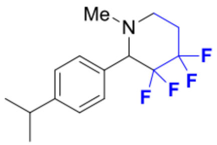

3b

$^{13}C\{^{1}H\}$ NMR
75 MHz
$CDCl_3$

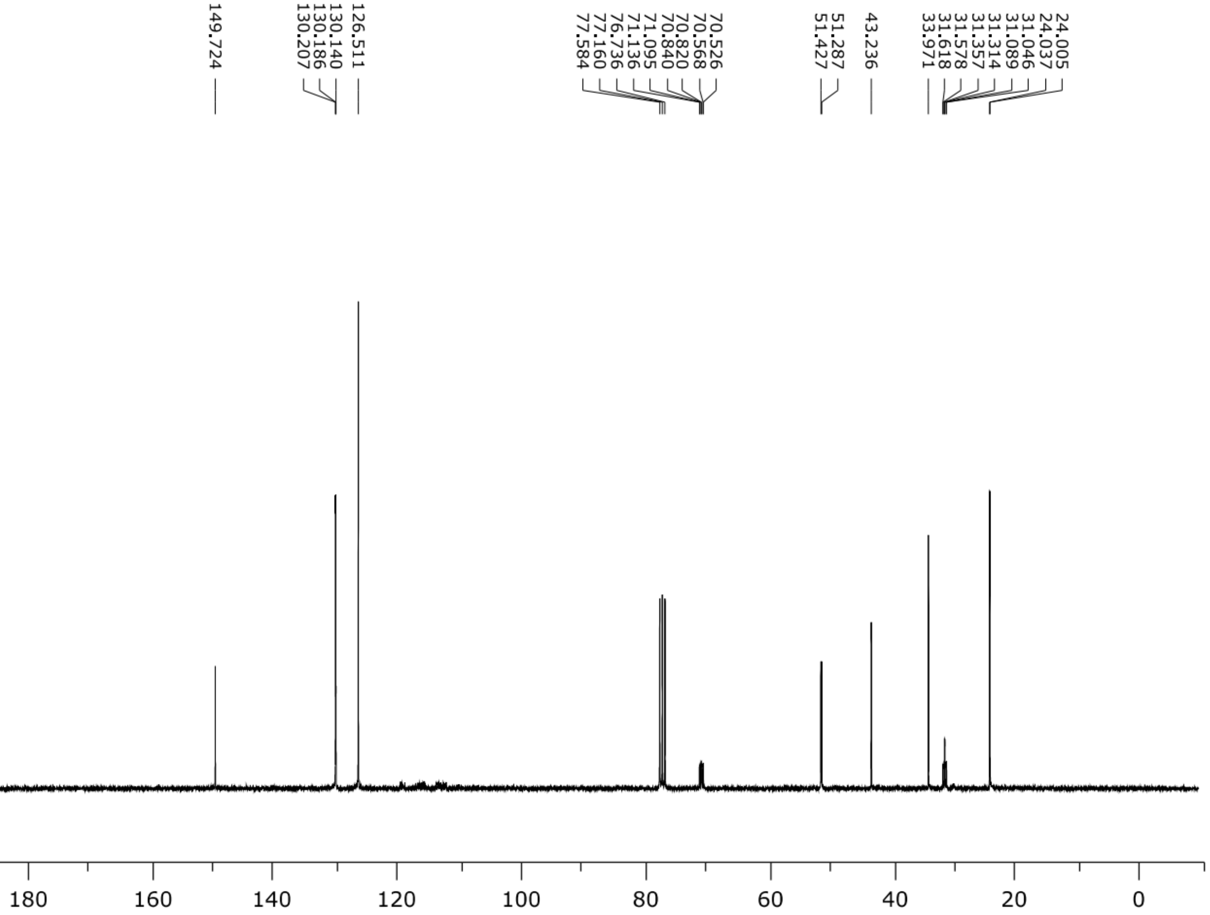

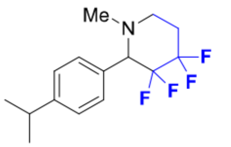

3b

^{19}F NMR
282 MHz
$CDCl_3$

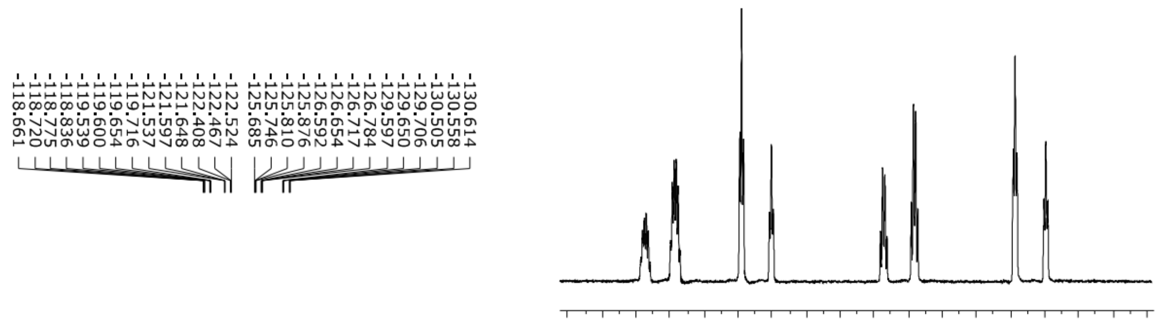

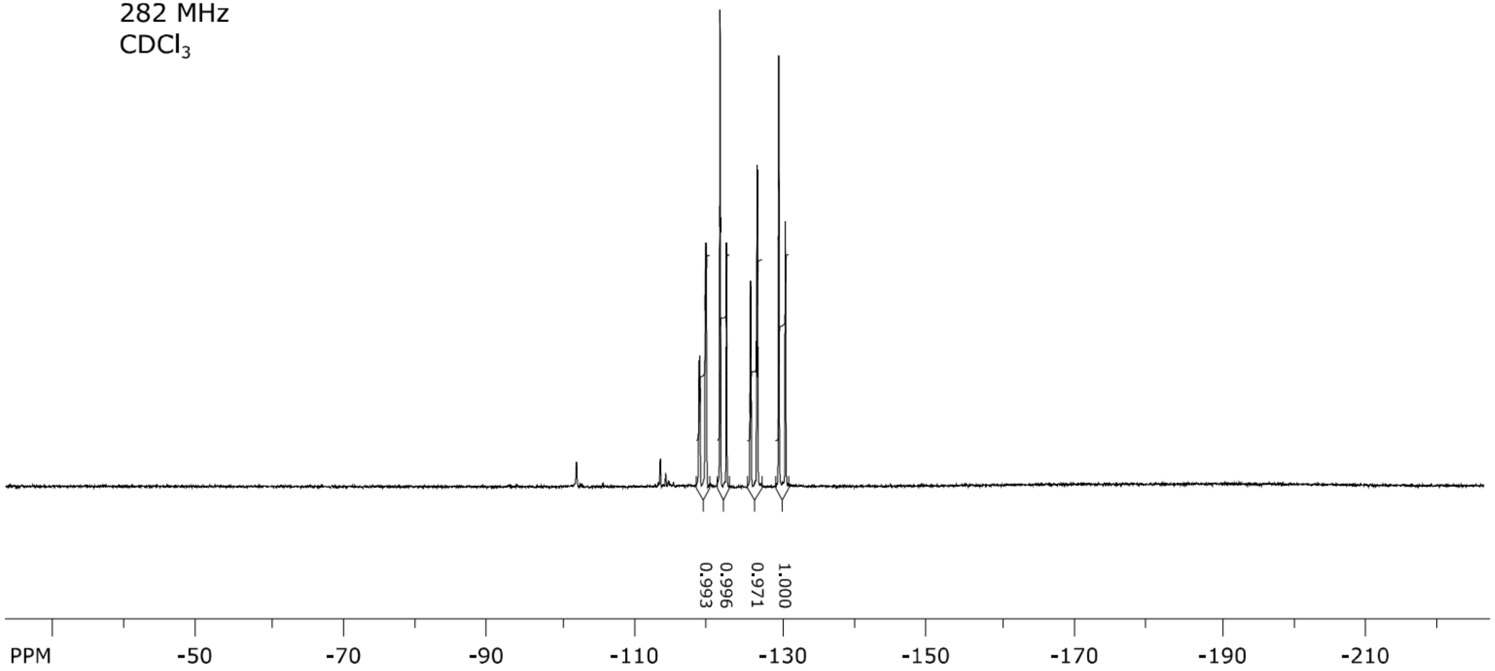

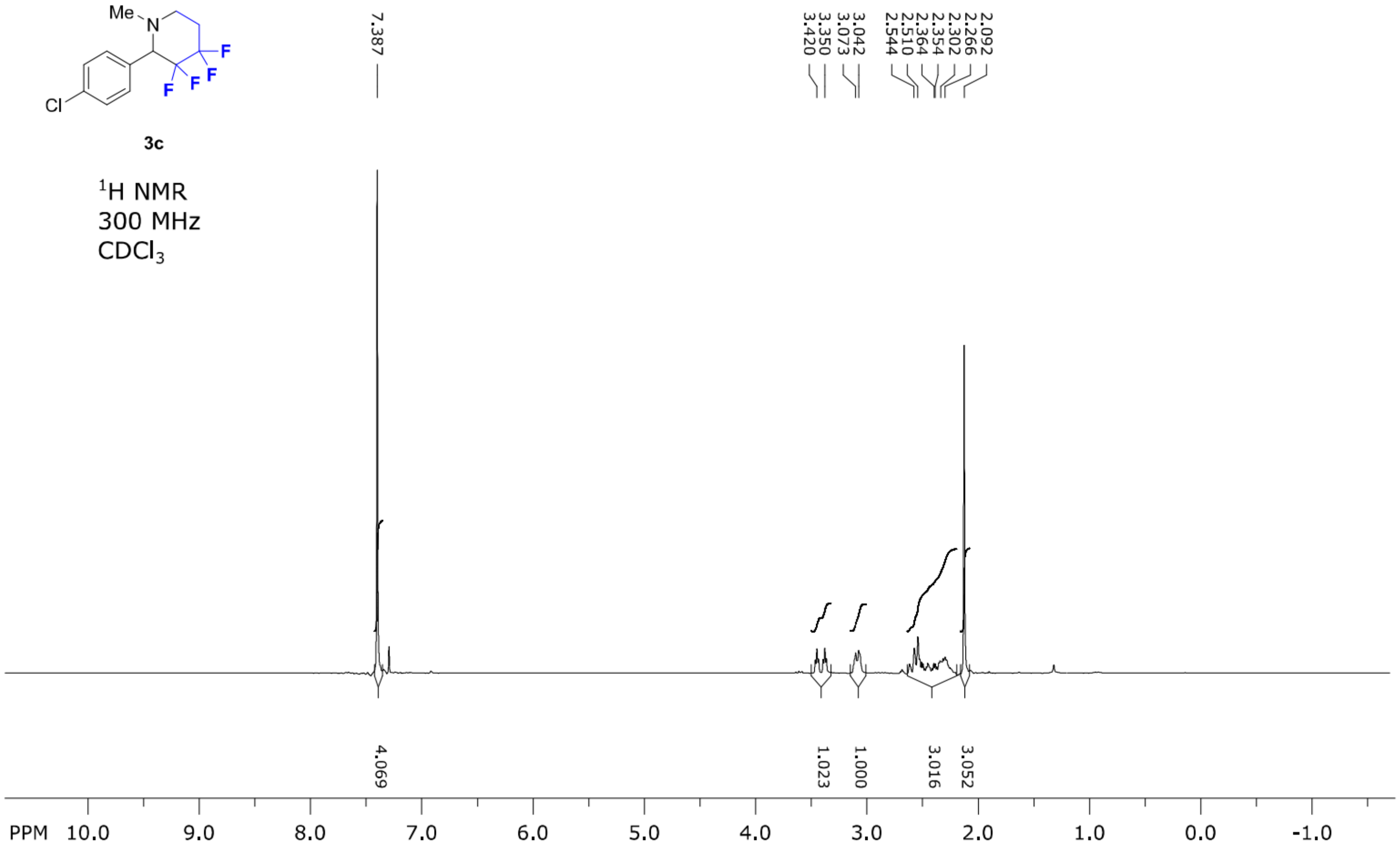
Me
N
F
F
F
F
Cl
3c
^{1}H NMR
300 MHz
$CDCl_3$
7.387
3.420
3.350
3.073
3.042
2.544
2.510
2.364
2.354
2.302
2.266
2.092
4.069
1.023
1.000
3.016
3.052
PPM 10.0 9.0 8.0 7.0 6.0 5.0 4.0 3.0 2.0 1.0 0.0 -1.0

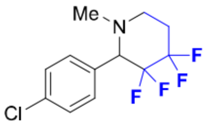

3c

$^{13}C\{^{1}H\}$ NMR
75 MHz
$CDCl_3$

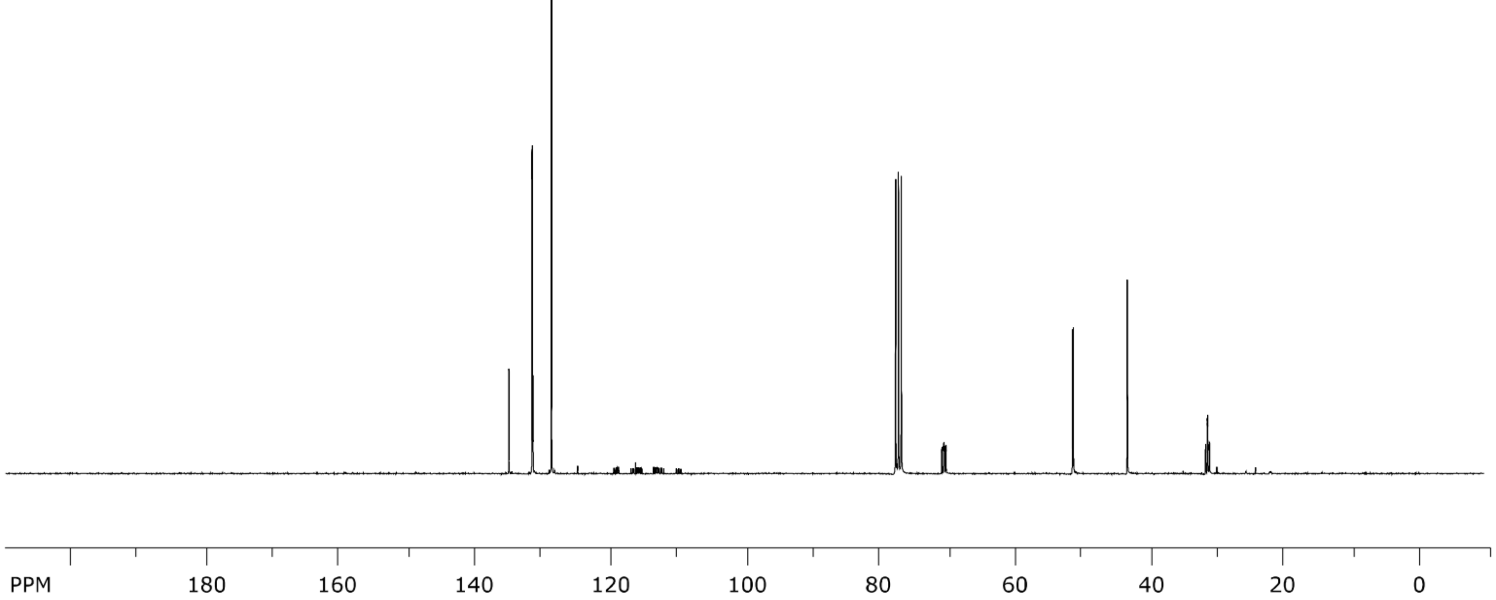

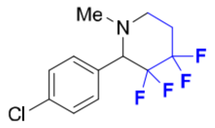

3c

^{19}F NMR
282 MHz
$CDCl_3$

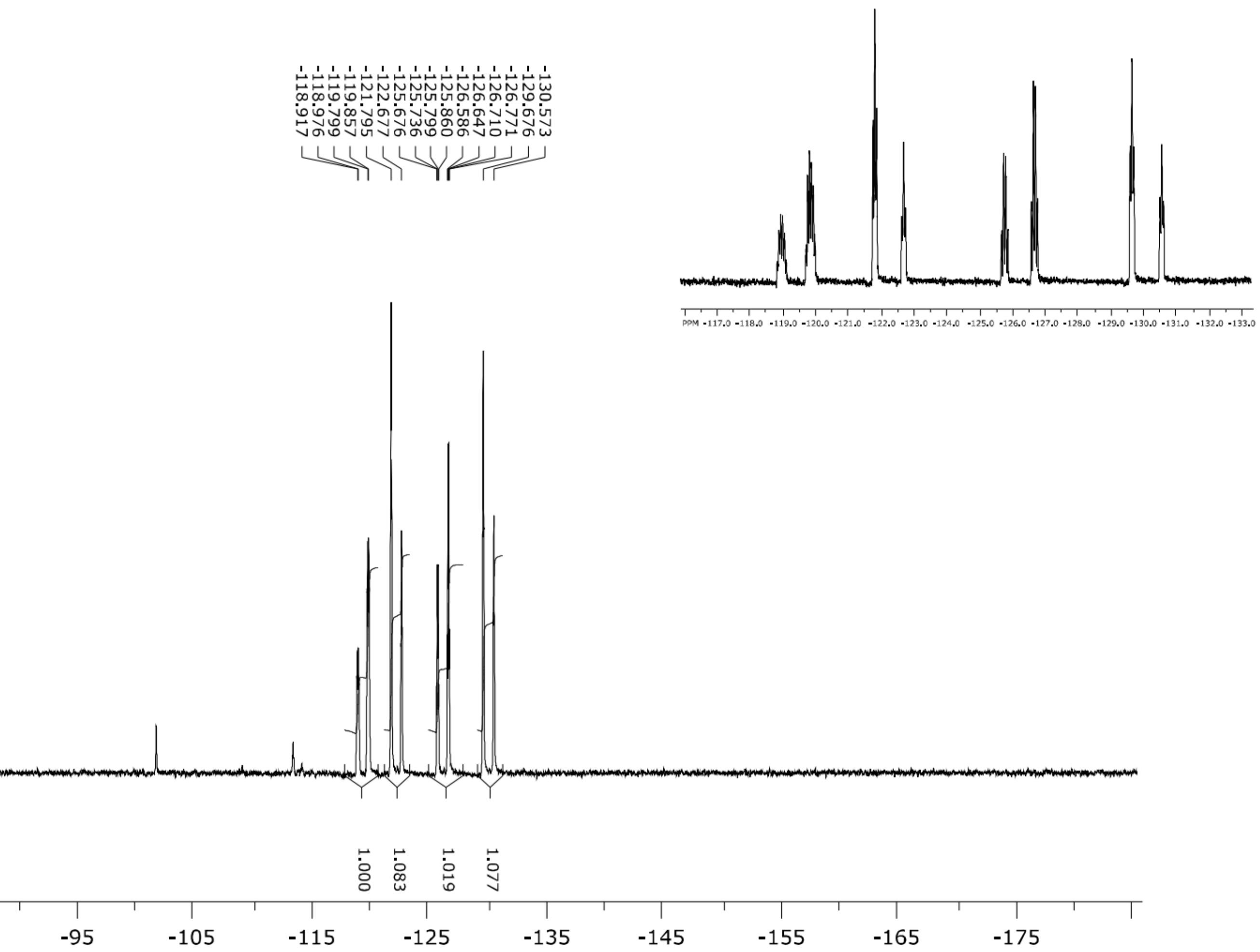

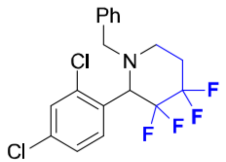

3d

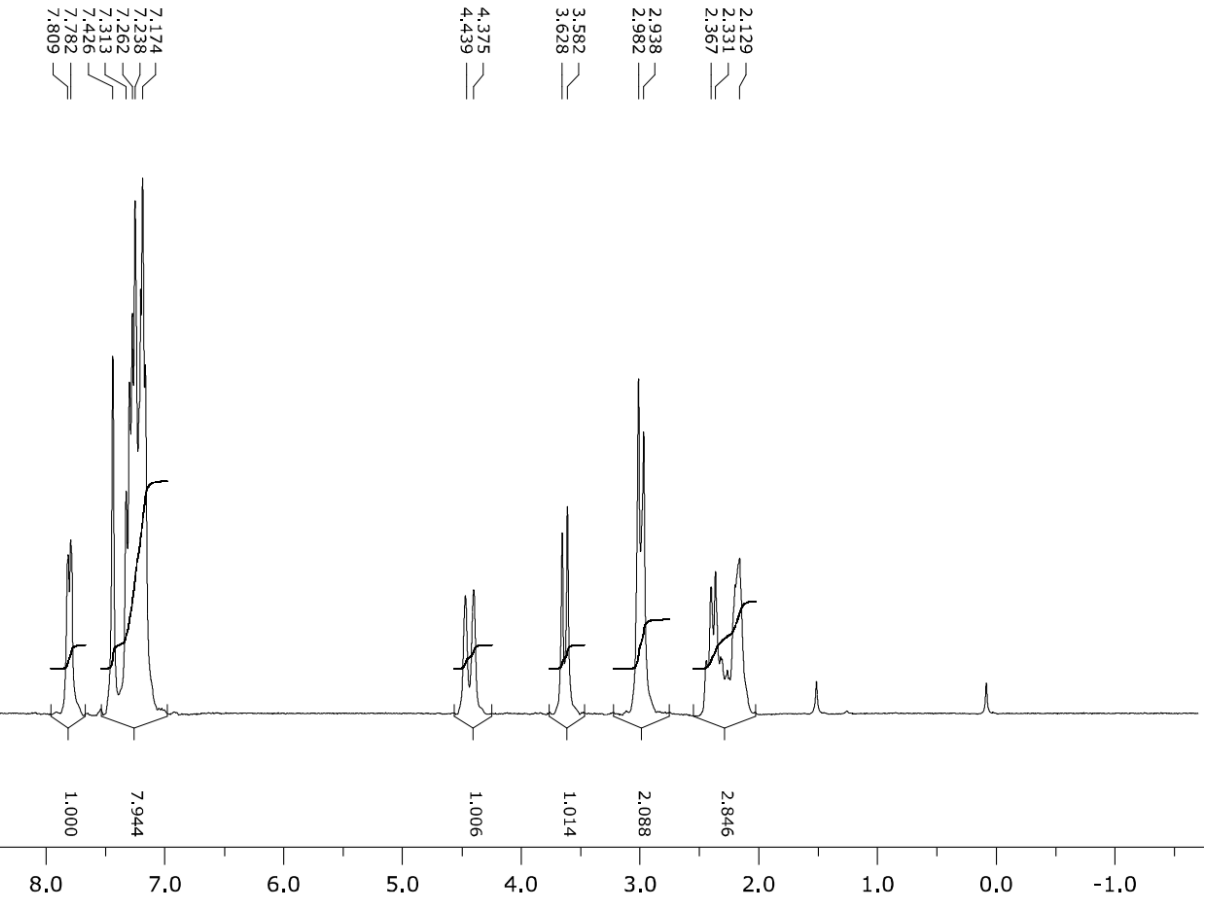

^{1}H NMR
300 MHz
$CDCl_3$
7.809
7.782
7.426
7.313
7.262
7.238
7.174
4.439
4.375
3.628
3.582
2.982
2.938
2.367
2.331
2.129
1.000
7.944
1.006
1.014
2.088
2.846
PPM 10.0 9.0 8.0 7.0 6.0 5.0 4.0 3.0 2.0 1.0 0.0 -1.0

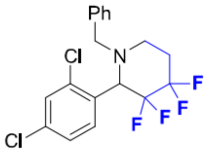

Ph
N
Cl
Cl
F
F
F
F

3d

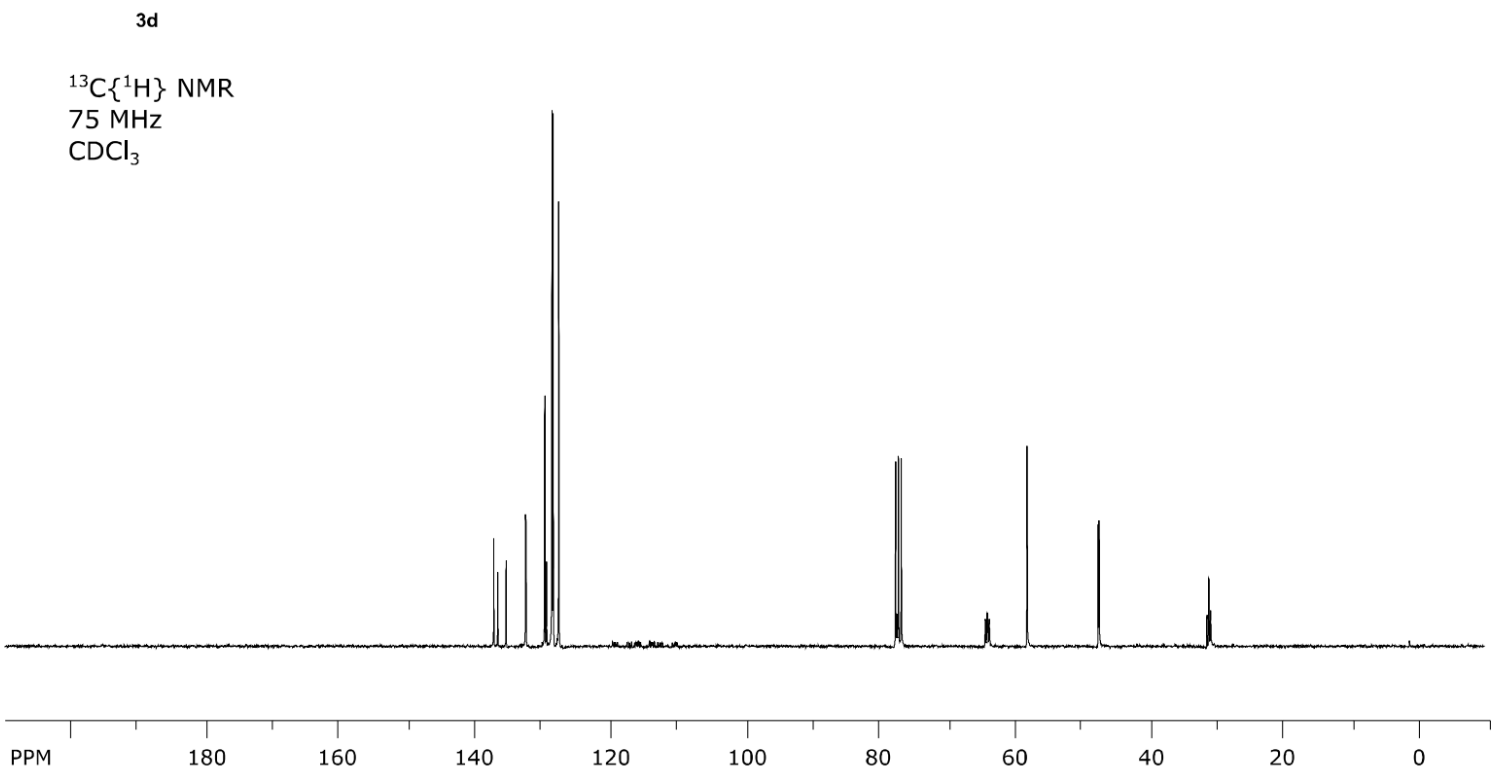

$^{13}C\{^1H\}$ NMR
75 MHz
$CDCl_3$
137.325
136.732
135.499
132.592
132.531
129.741
129.484
128.687
128.501
127.667
77.583
77.160
76.737
64.235
63.957
63.656
58.035
47.457
47.319
31.284
31.001
30.742
PPM
180
160
140
120
100
80
60
40
20
0

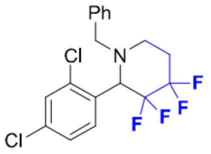

3d

^{19}F NMR
282 MHz
$CDCl_3$

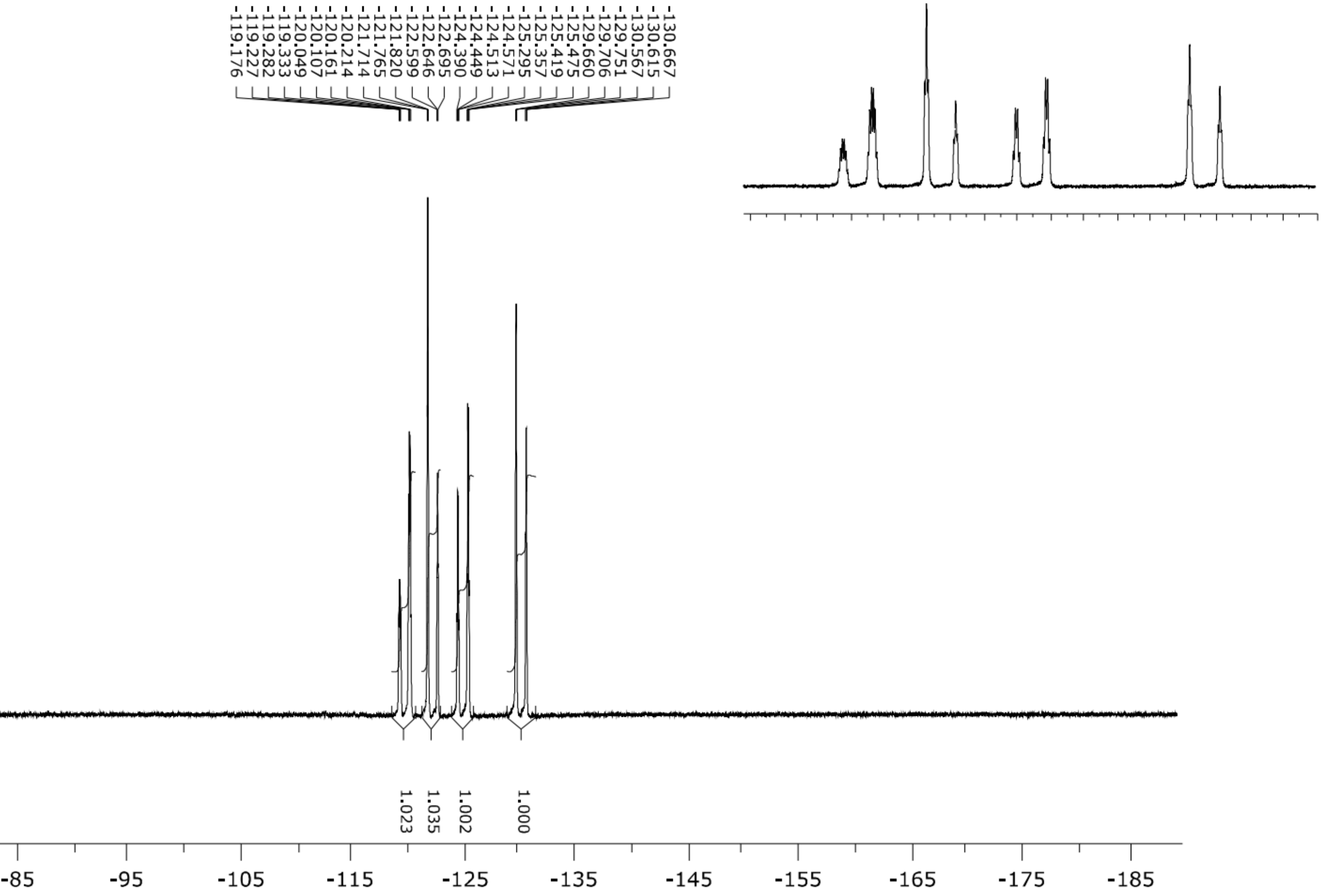

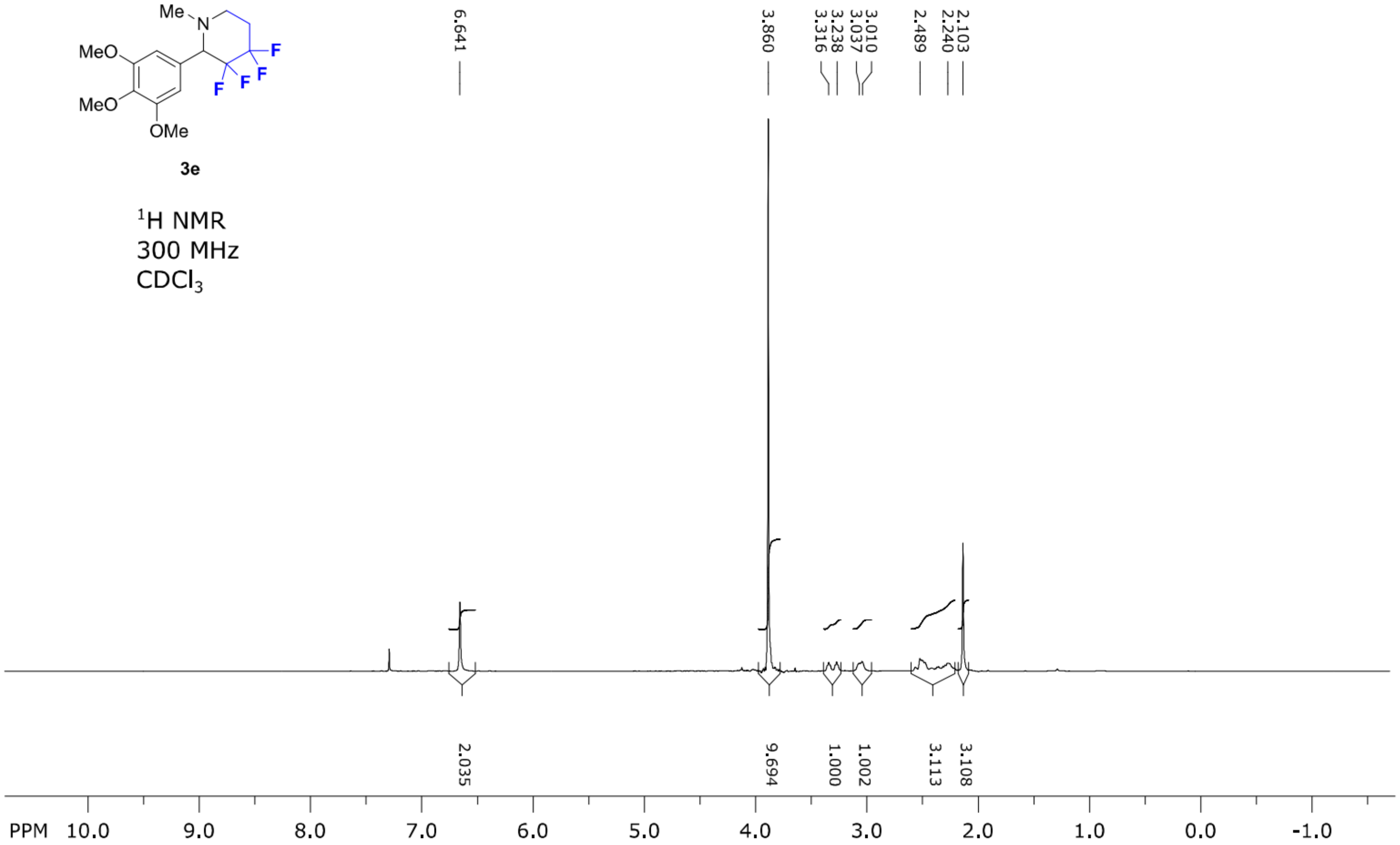

3e

^{1}H NMR
300 MHz
$CDCl_3$

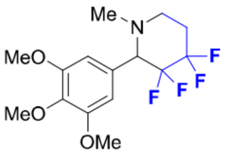

3e

$^{13}C\{^{1}H\}$ NMR
75 MHz
$CDCl_3$

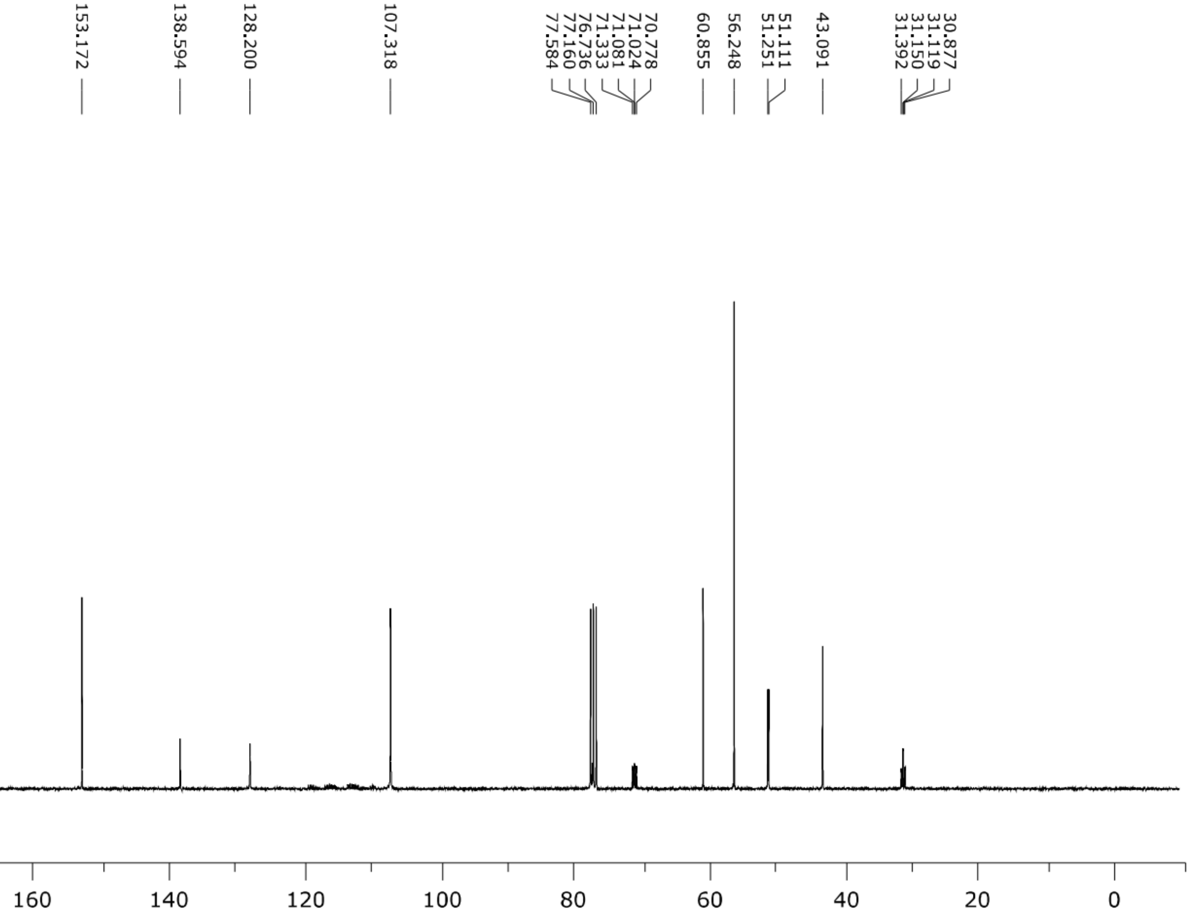

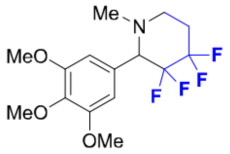

3e

^{19}F NMR
282 MHz
$CDCl_3$

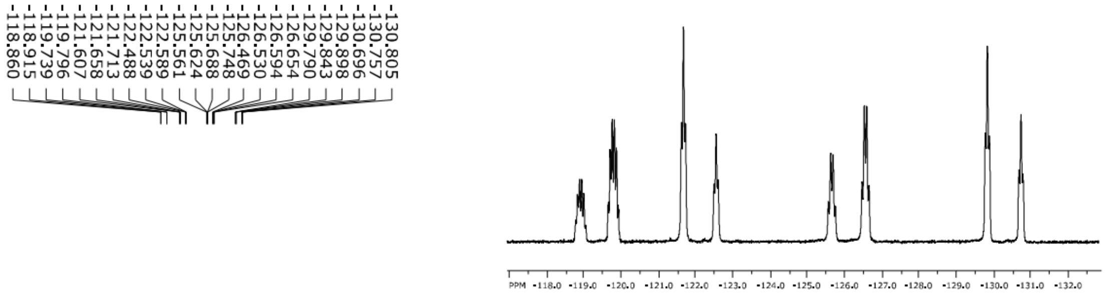
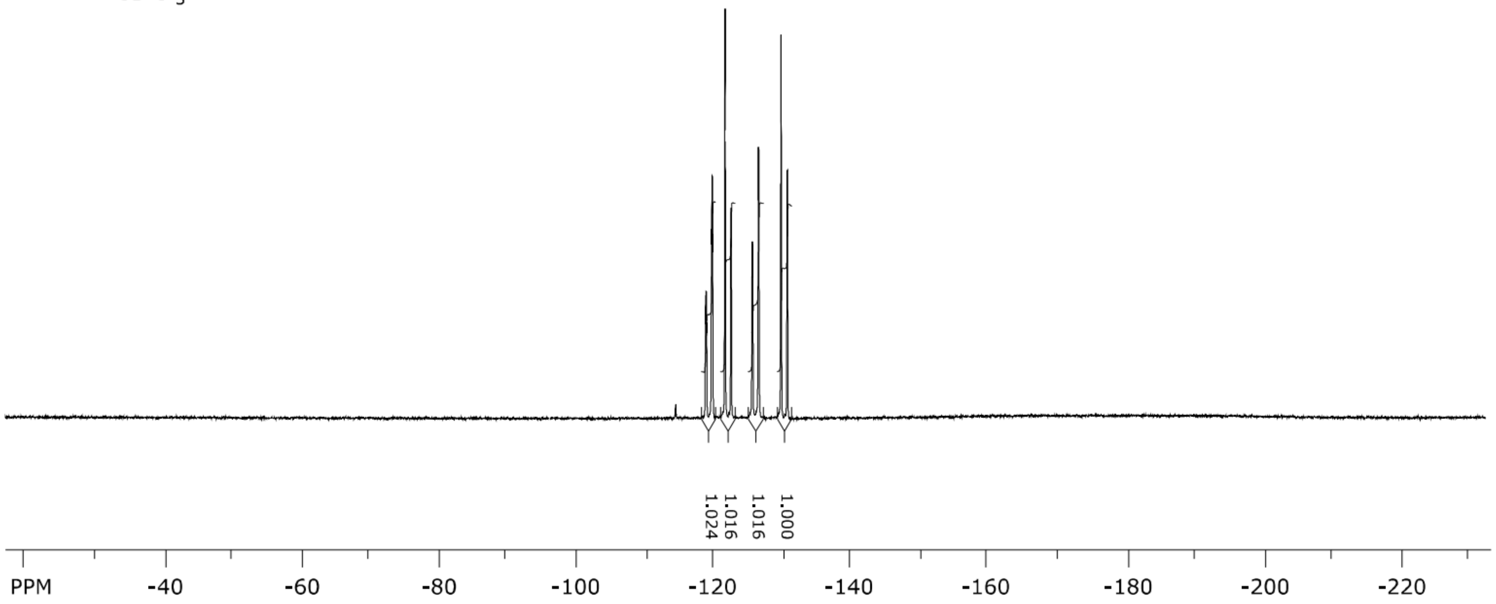

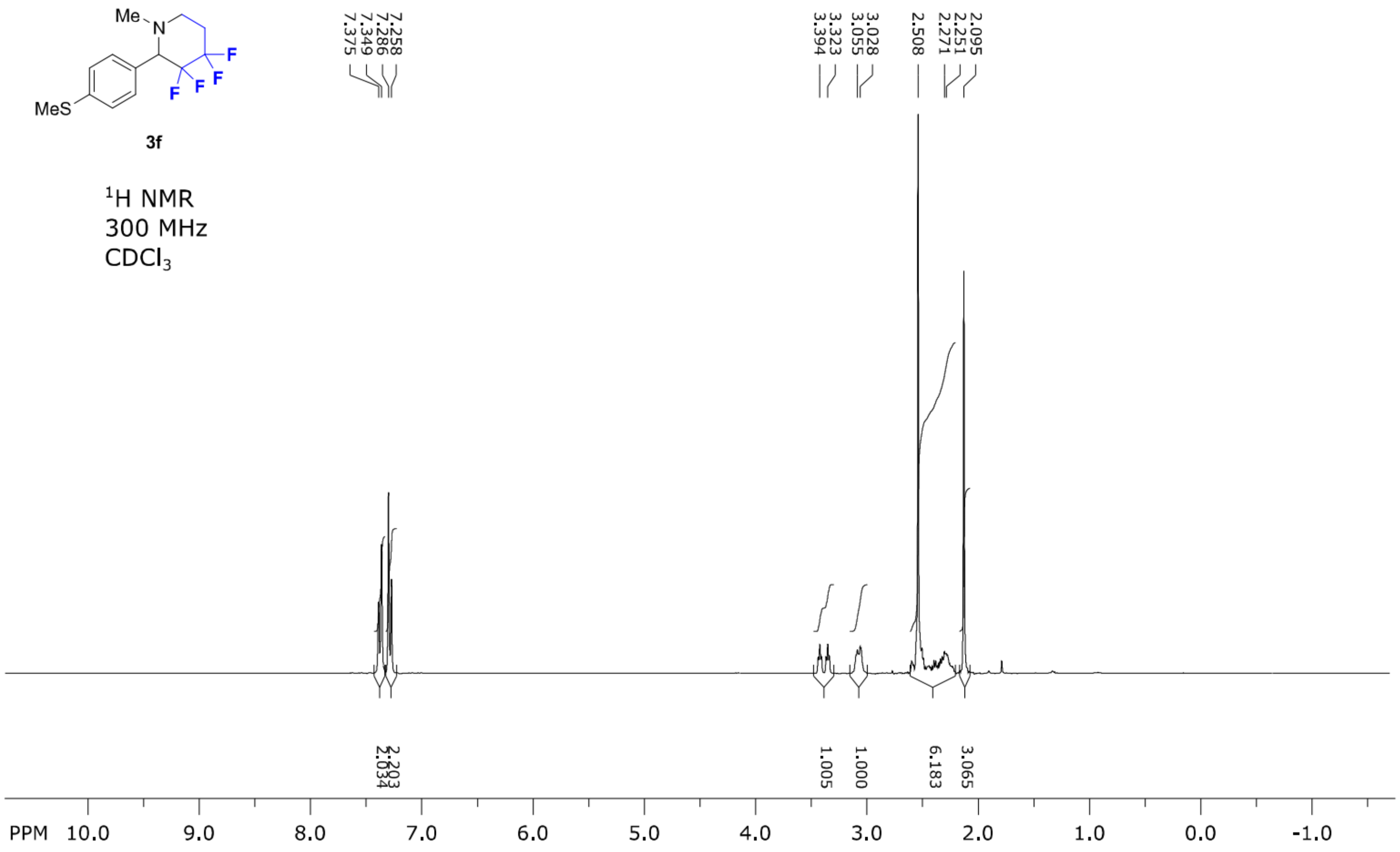

Me
N
F
F
F
F
MeS
3f
¹H NMR
300 MHz
CDCl3
7.375
7.349
7.286
7.258
3.394
3.323
3.055
3.028
2.508
2.271
2.251
2.095
2.034
2.203
1.005
1.000
6.183
3.065
PPM 10.0 9.0 8.0 7.0 6.0 5.0 4.0 3.0 2.0 1.0 0.0 -1.0

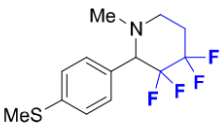

3f

$^{13}C\{^{1}H\}$ NMR
75 MHz
$CDCl_3$

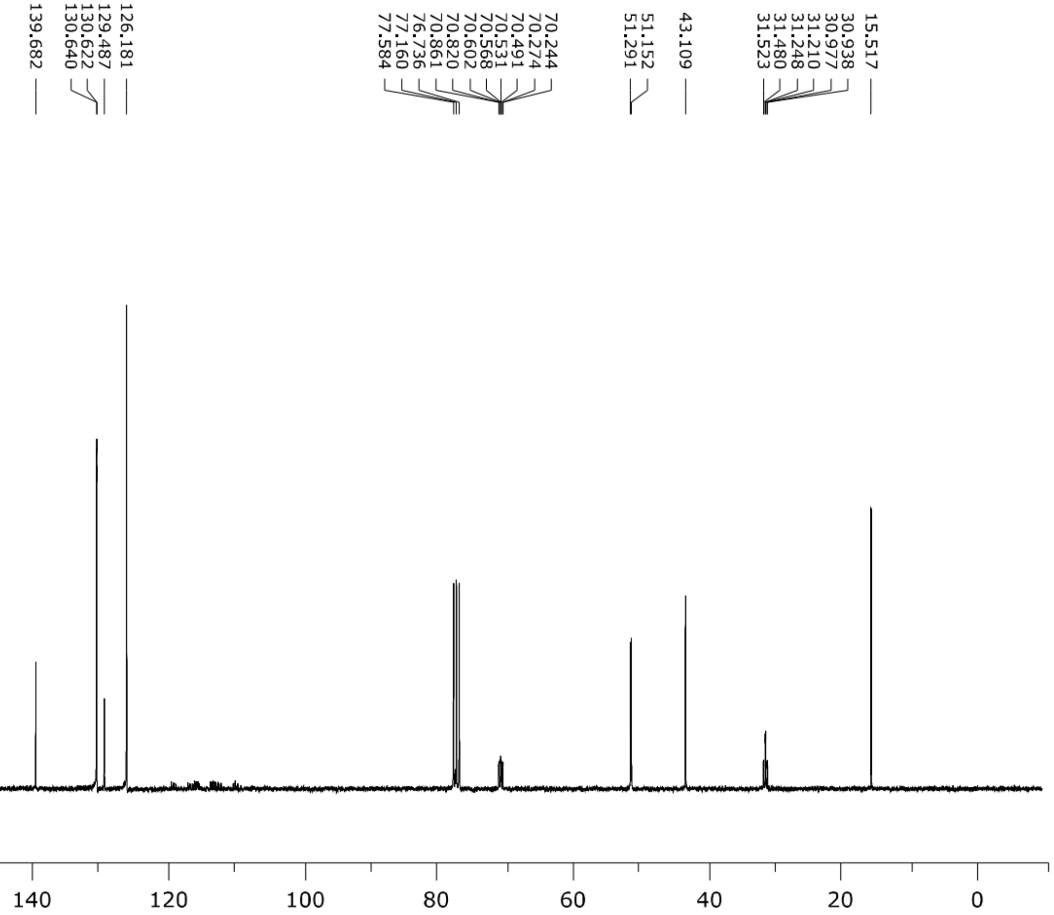

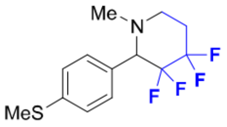

3f

^{19}F NMR
282 MHz
$CDCl_3$

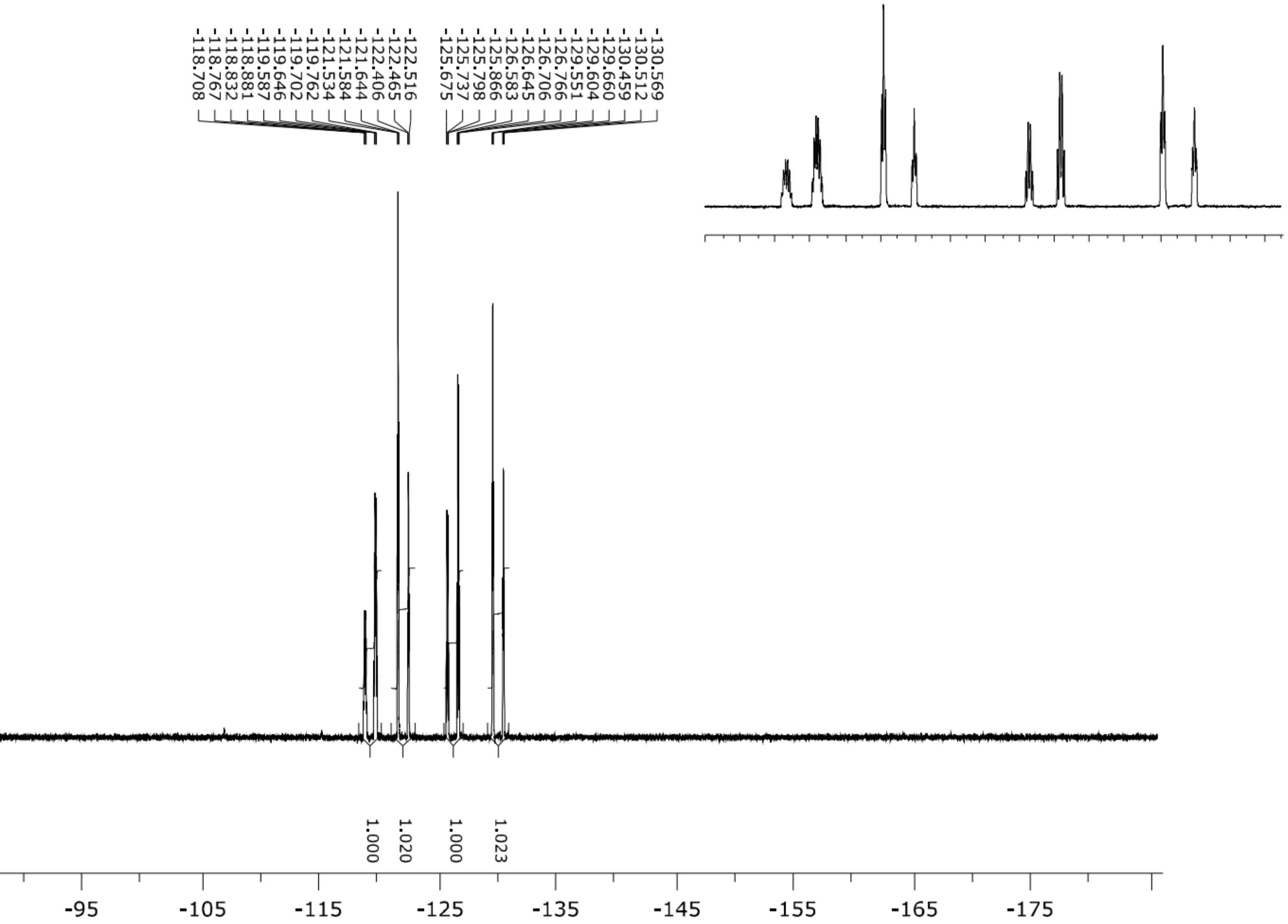

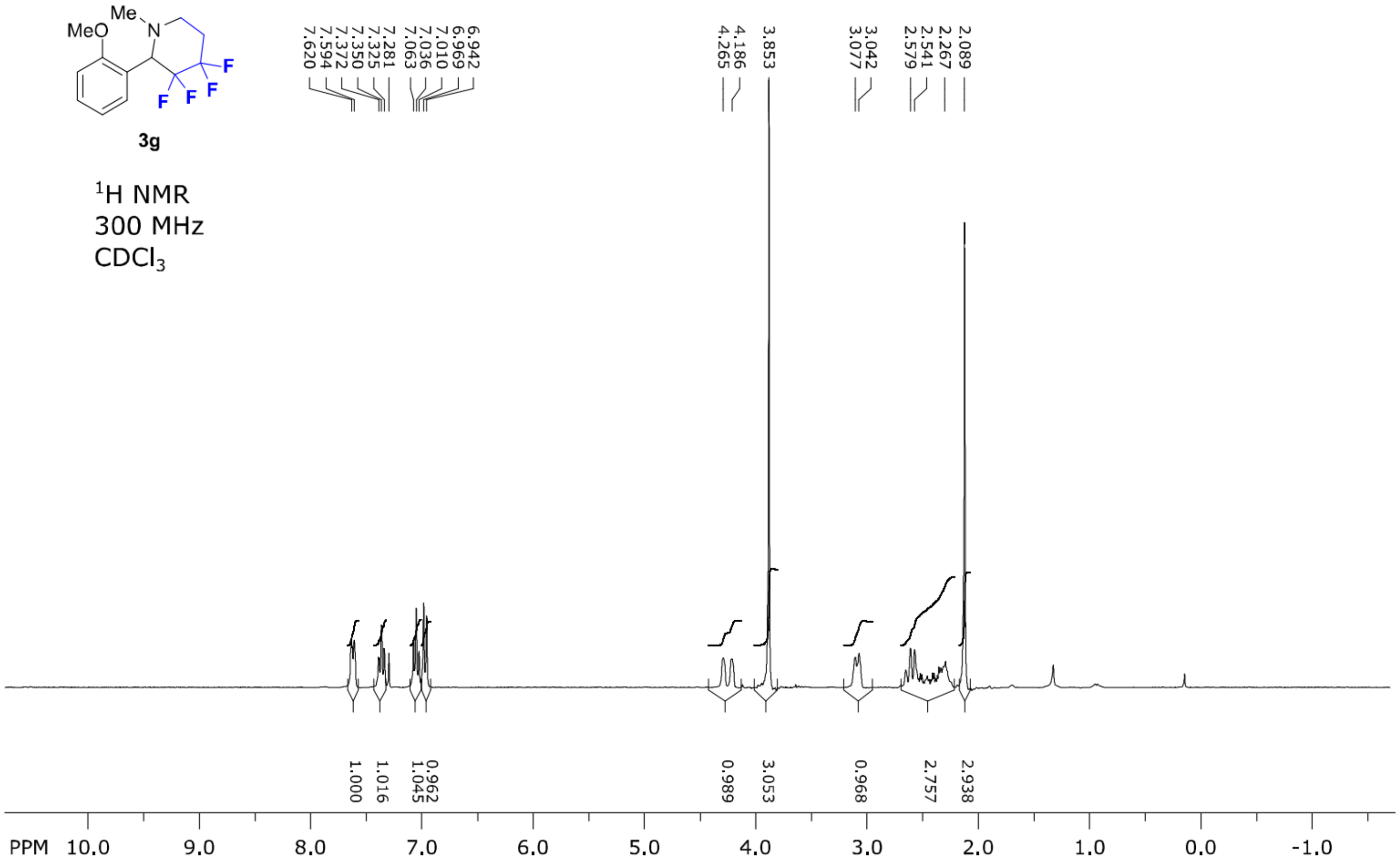

Me
MeO
N
F
F
F
F
3g
¹H NMR
300 MHz
CDCl3
7.620
7.594
7.372
7.350
7.325
7.281
7.063
7.036
7.010
6.969
6.942
4.265
4.186
3.853
3.077
3.042
2.579
2.541
2.267
2.089
1.000
1.016
1.045
0.962
0.989
3.053
0.968
2.757
2.938
PPM 10.0 9.0 8.0 7.0 6.0 5.0 4.0 3.0 2.0 1.0 0.0 -1.0

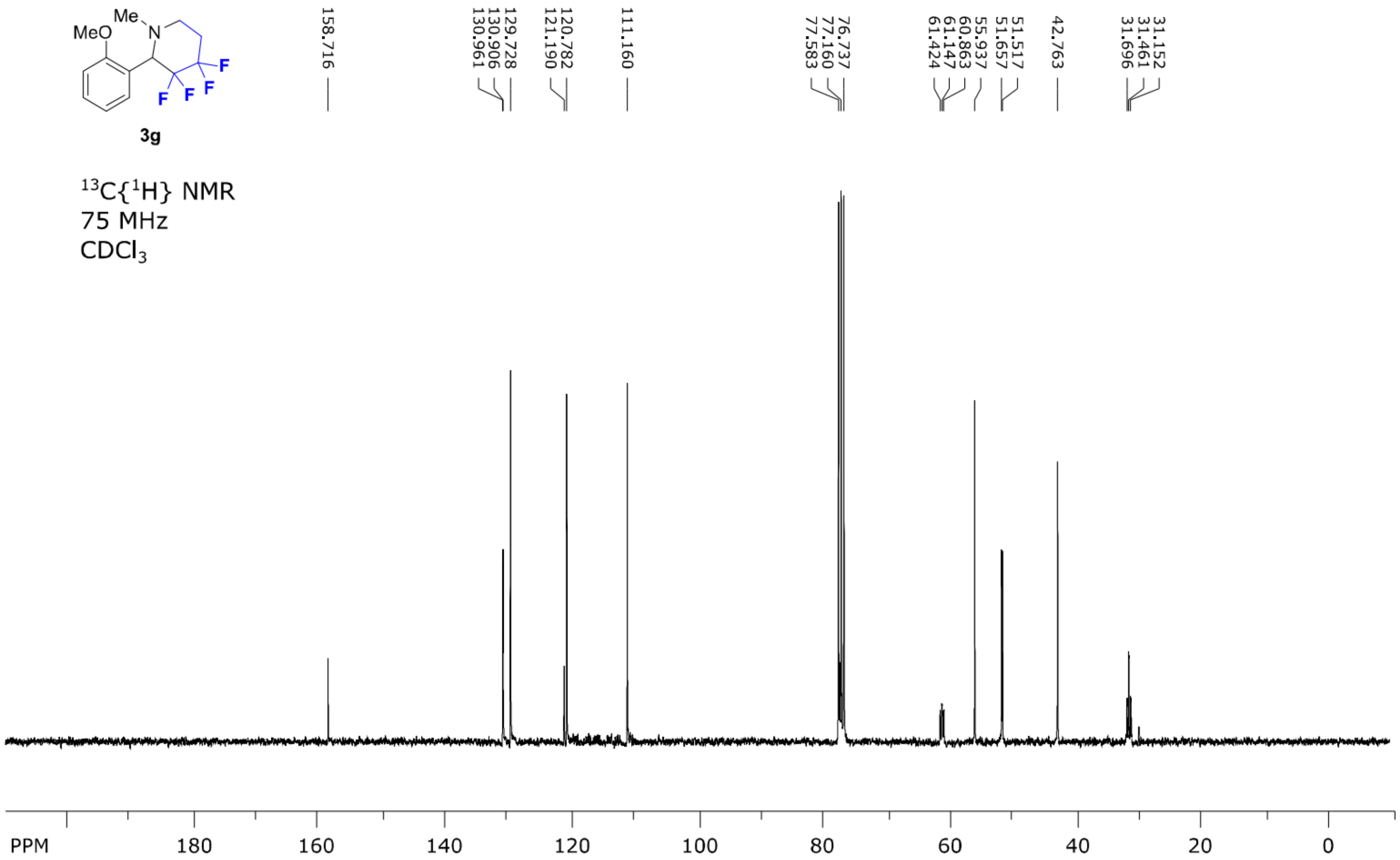
MeO
Me
N
F
F
F
F
3g
$^{13}C\{^{1}H\}$ NMR
75 MHz
$CDCl_3$
158.716
130.961
130.906
129.728
121.190
120.782
111.160
77.583
77.160
76.737
61.424
61.147
60.863
55.937
51.657
51.517
42.763
31.696
31.461
31.152
PPM
180
160
140
120
100
80
60
40
20
0

3g

^{19}F NMR
282 MHz
$CDCl_3$

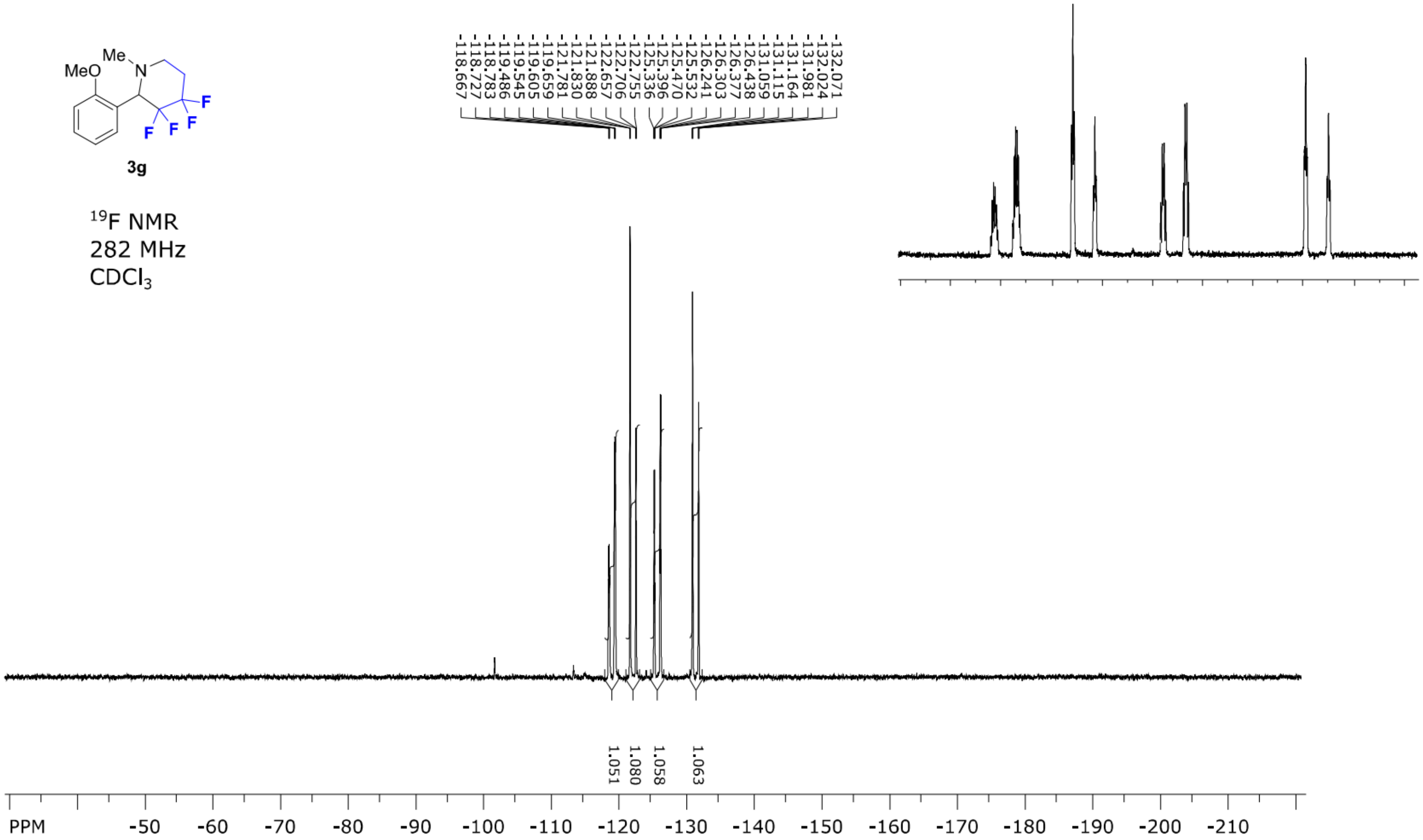

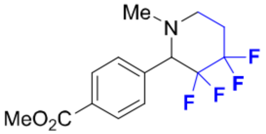

3h

^{1}H NMR
300 MHz
$CDCl_3$

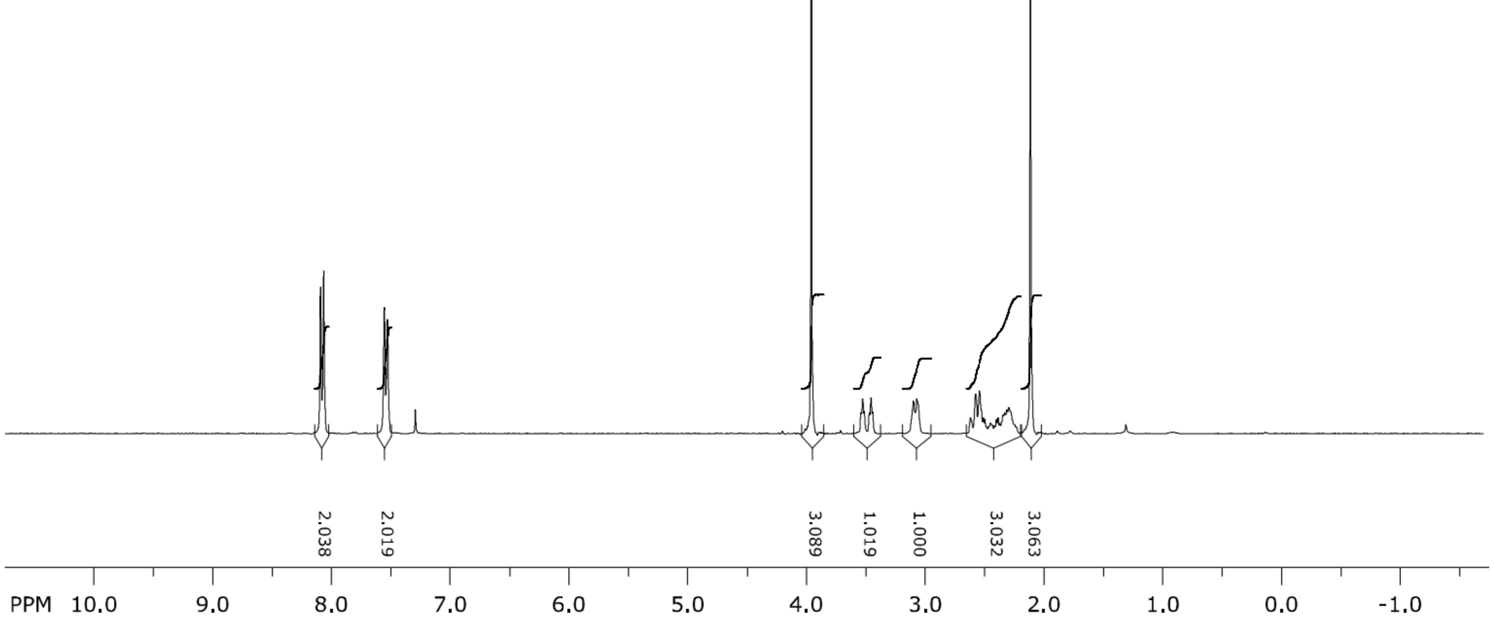

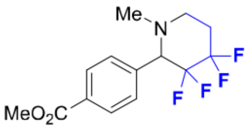
3h

$^{13}C\{^1H\}$ NMR
75 MHz
$CDCl_3$

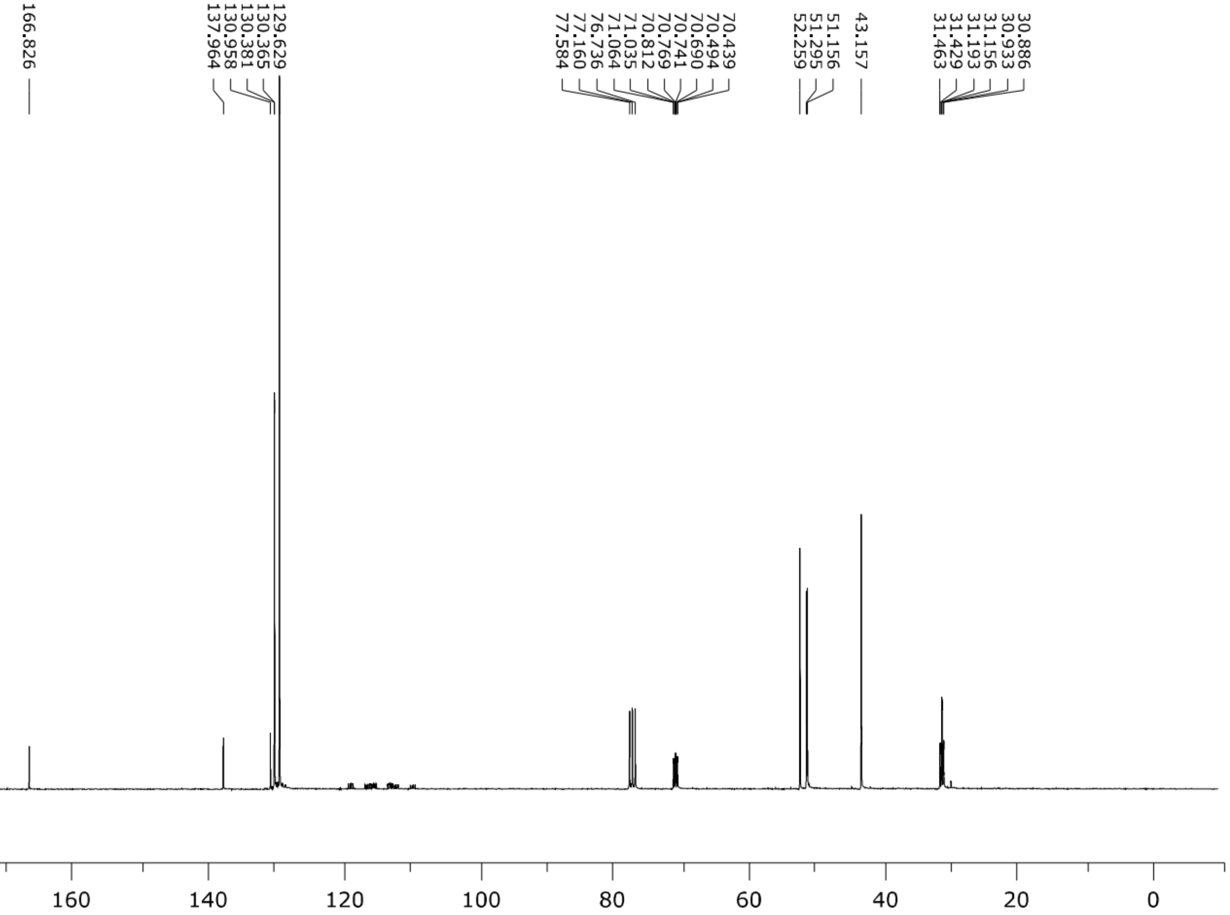

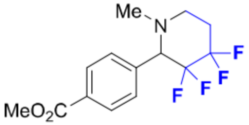

3h

^{19}F NMR
282 MHz
$CDCl_3$

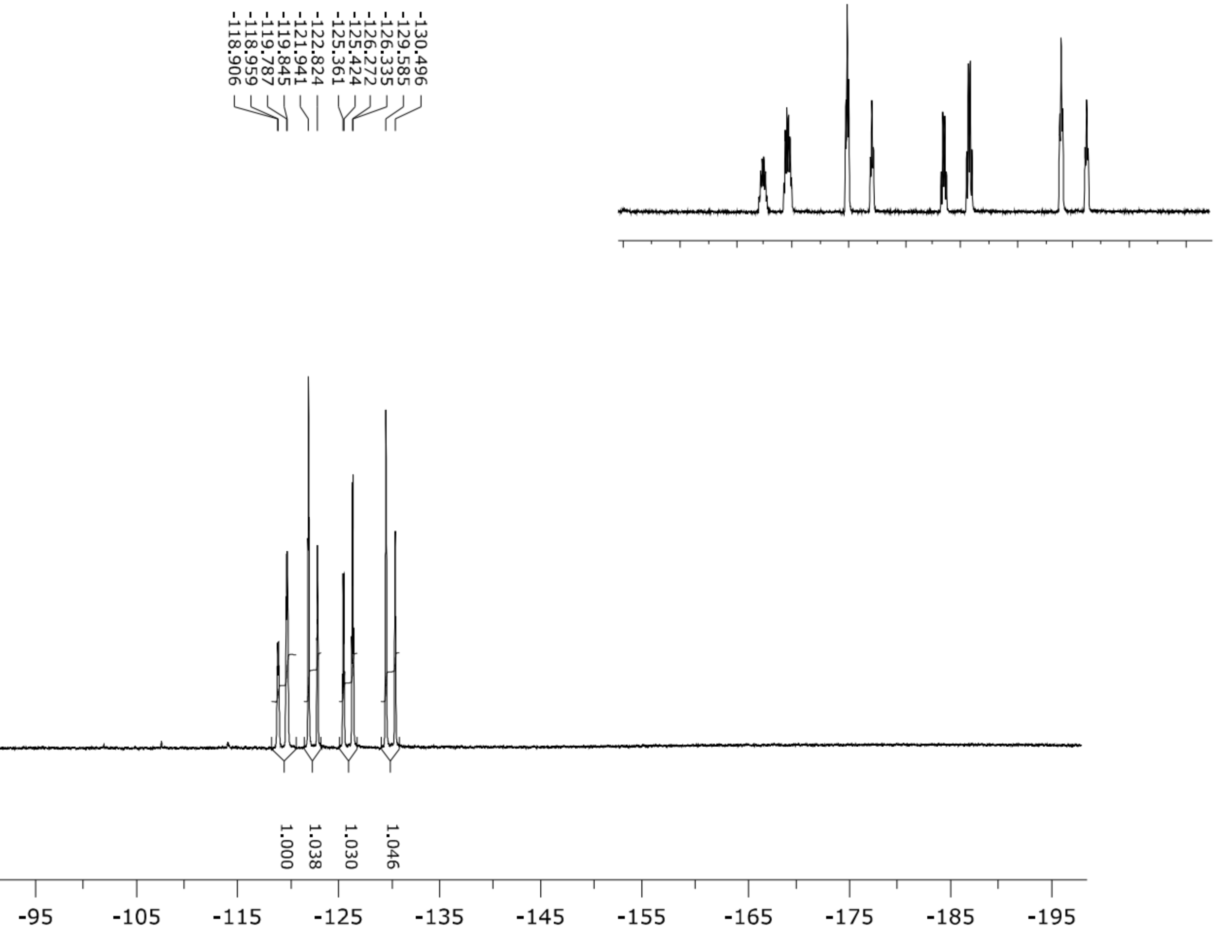

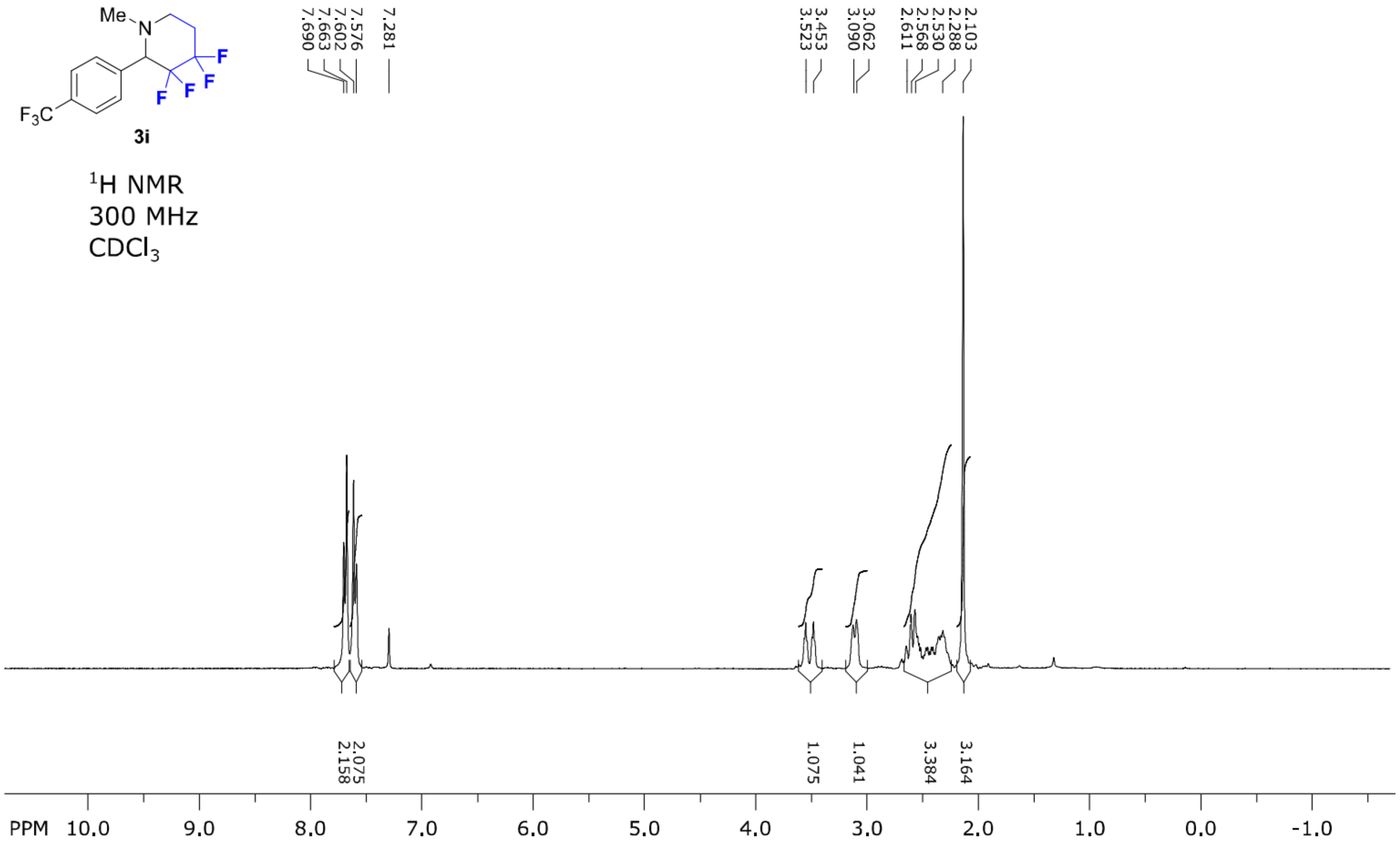

Me
N
F
F
F
F
F3C
3i
1H NMR
300 MHz
CDCl3
7.690
7.663
7.602
7.576
7.281
3.523
3.453
3.090
3.062
2.611
2.568
2.530
2.288
2.103
2.158
2.075
1.075
1.041
3.384
3.164
PPM 10.0 9.0 8.0 7.0 6.0 5.0 4.0 3.0 2.0 1.0 0.0 -1.0

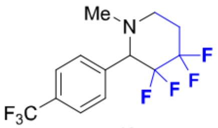

3i

$^{13}C\{^{1}H\}$ NMR
75 MHz
$CDCl_3$

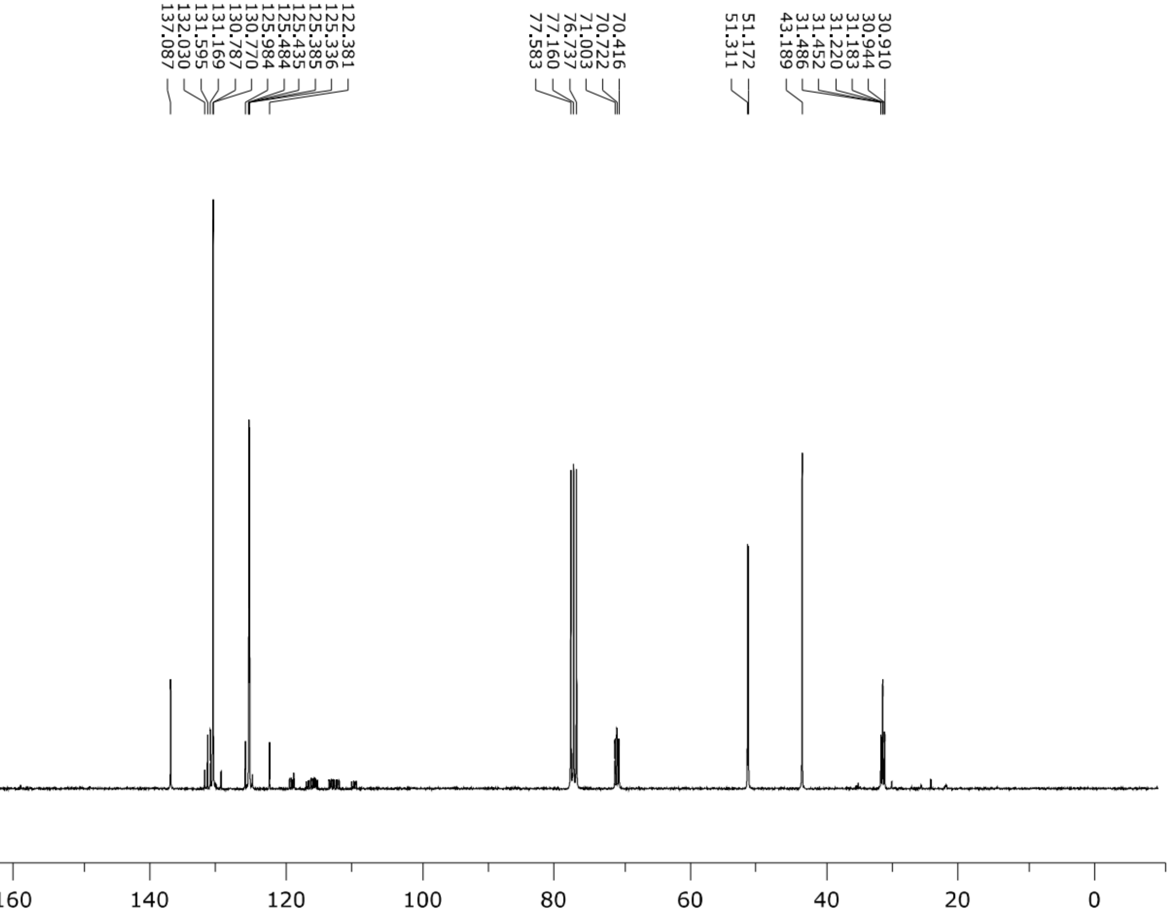

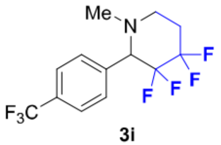

3i

^{19}F NMR
282 MHz
$CDCl_3$

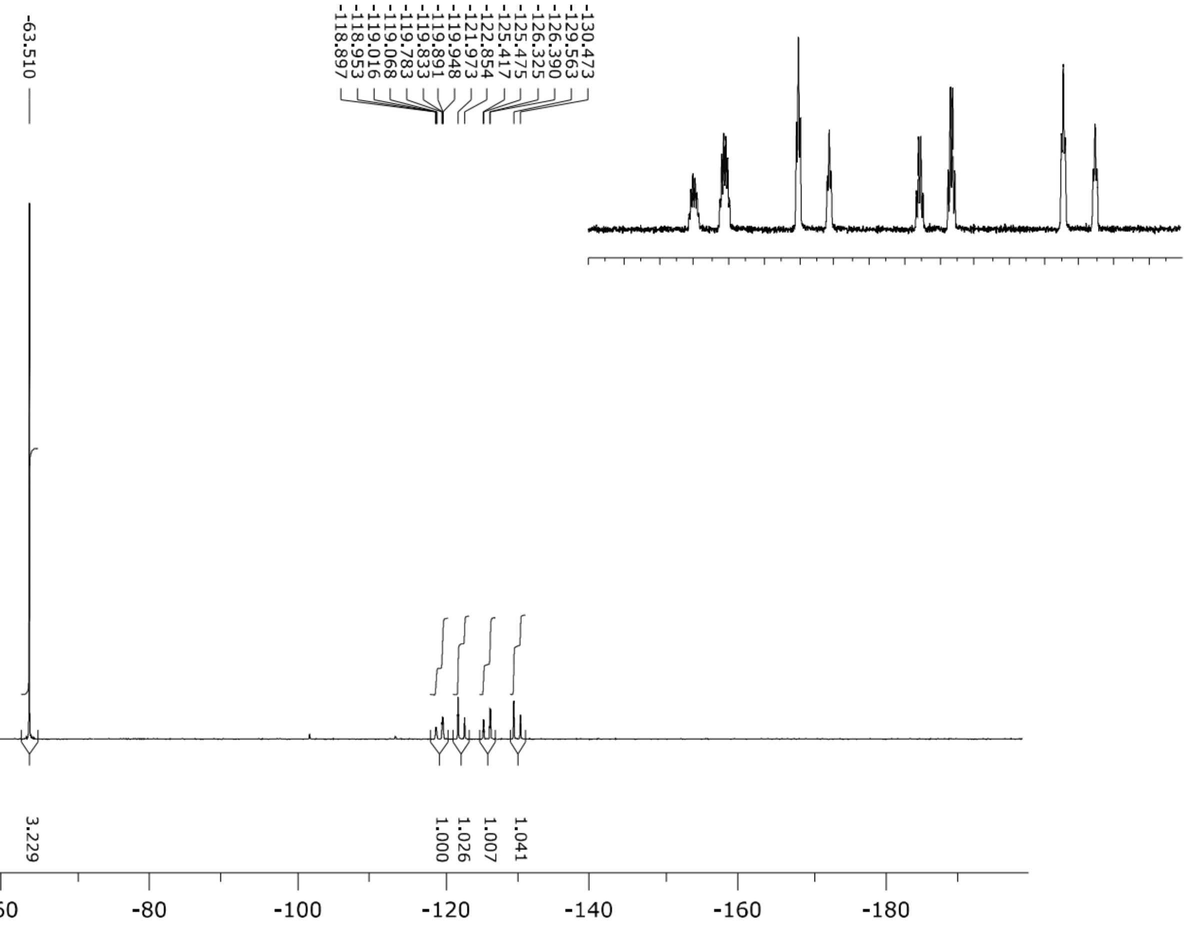

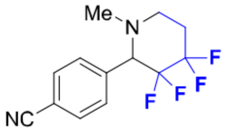

3j

^{1}H NMR
300 MHz
$CDCl_3$

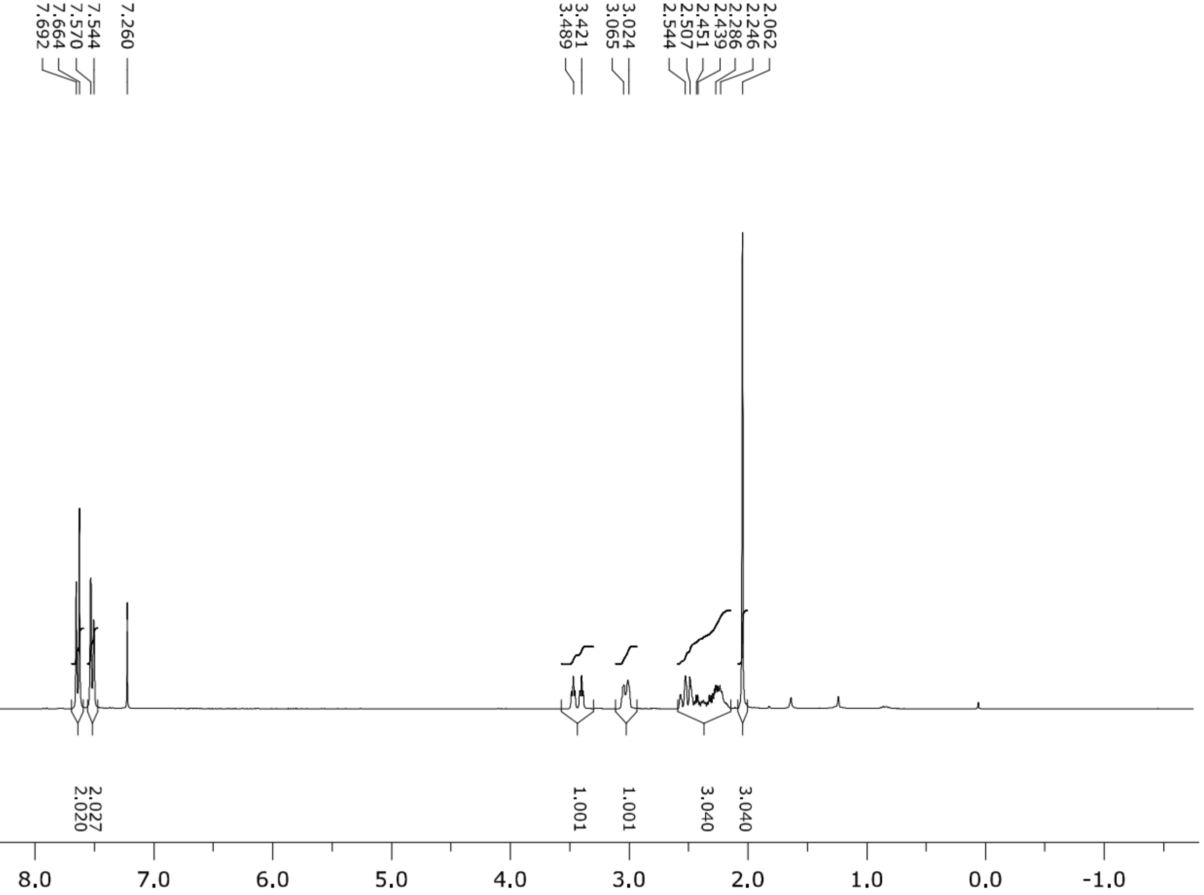

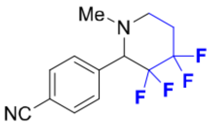

3j

$^{13}C\{^1H\}$ NMR
75 MHz
$CDCl_3$

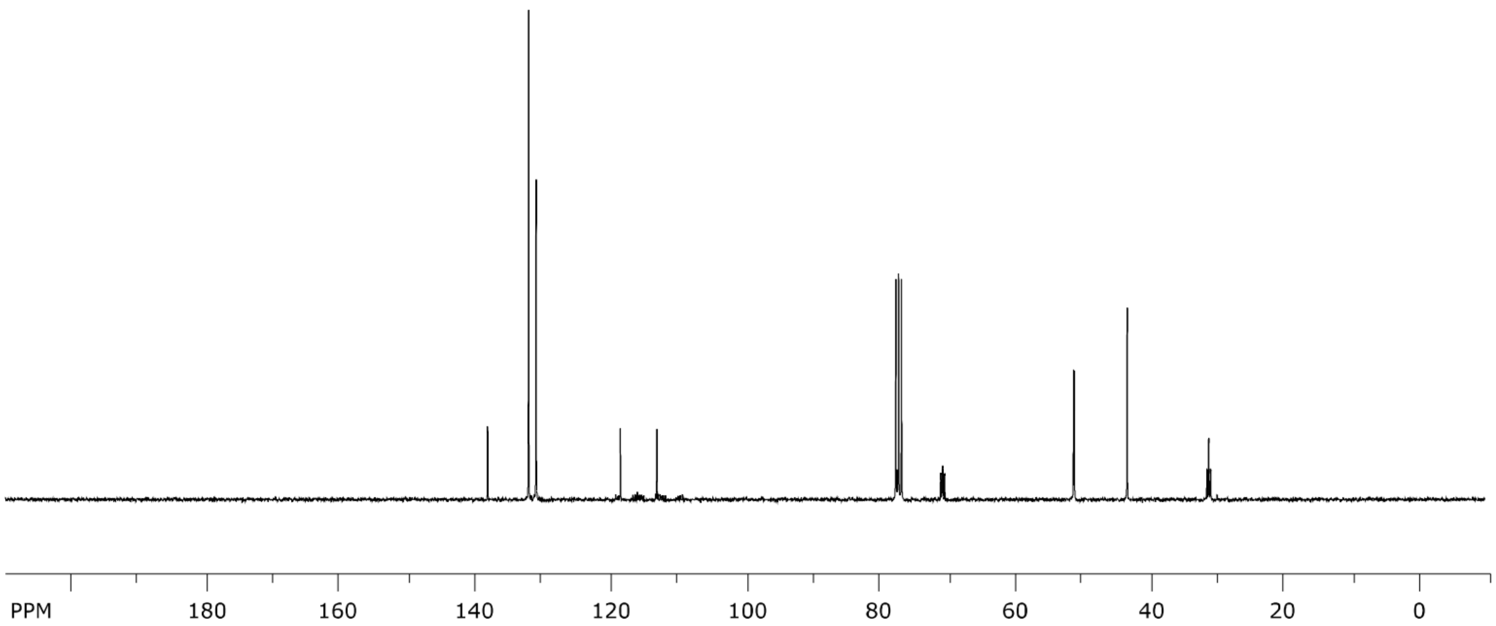

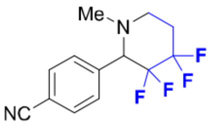

3j

^{19}F NMR
282 MHz
$CDCl_3$

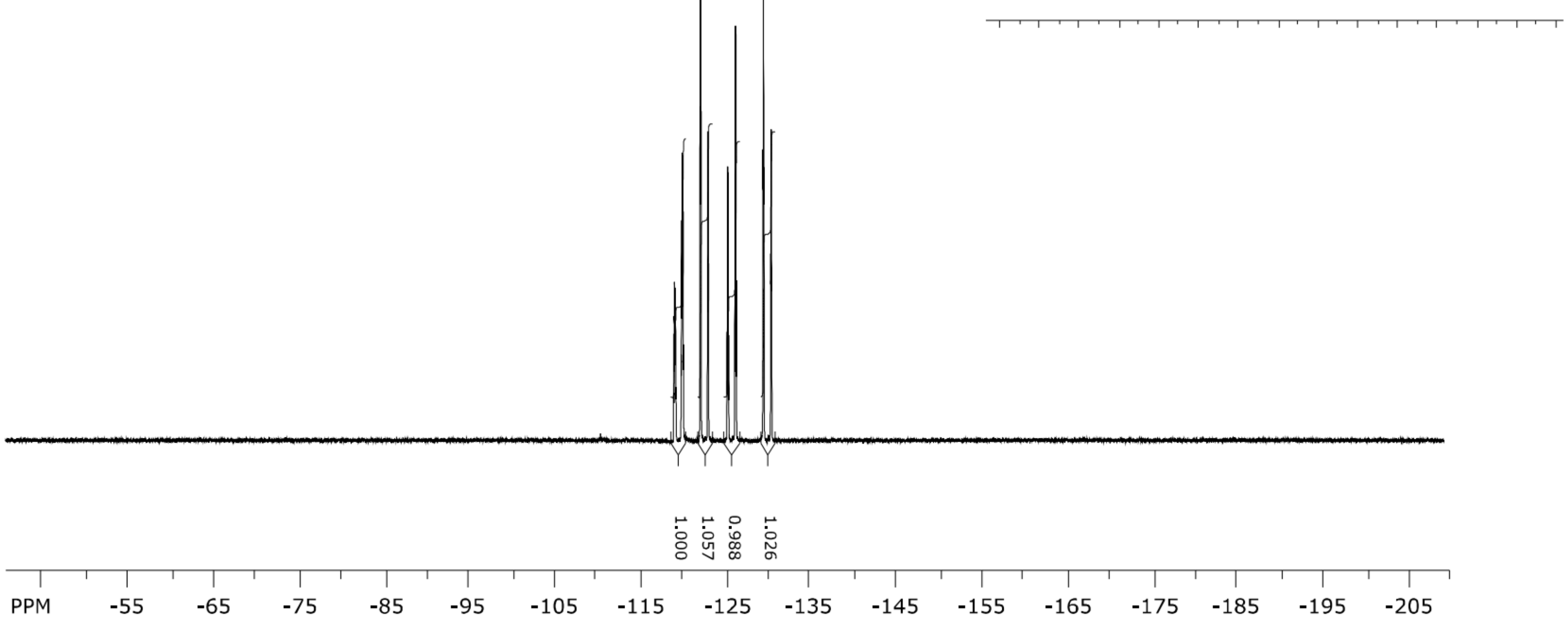

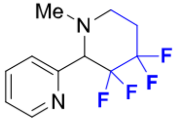

3k

^1H NMR
300 MHz
$CDCl_3$

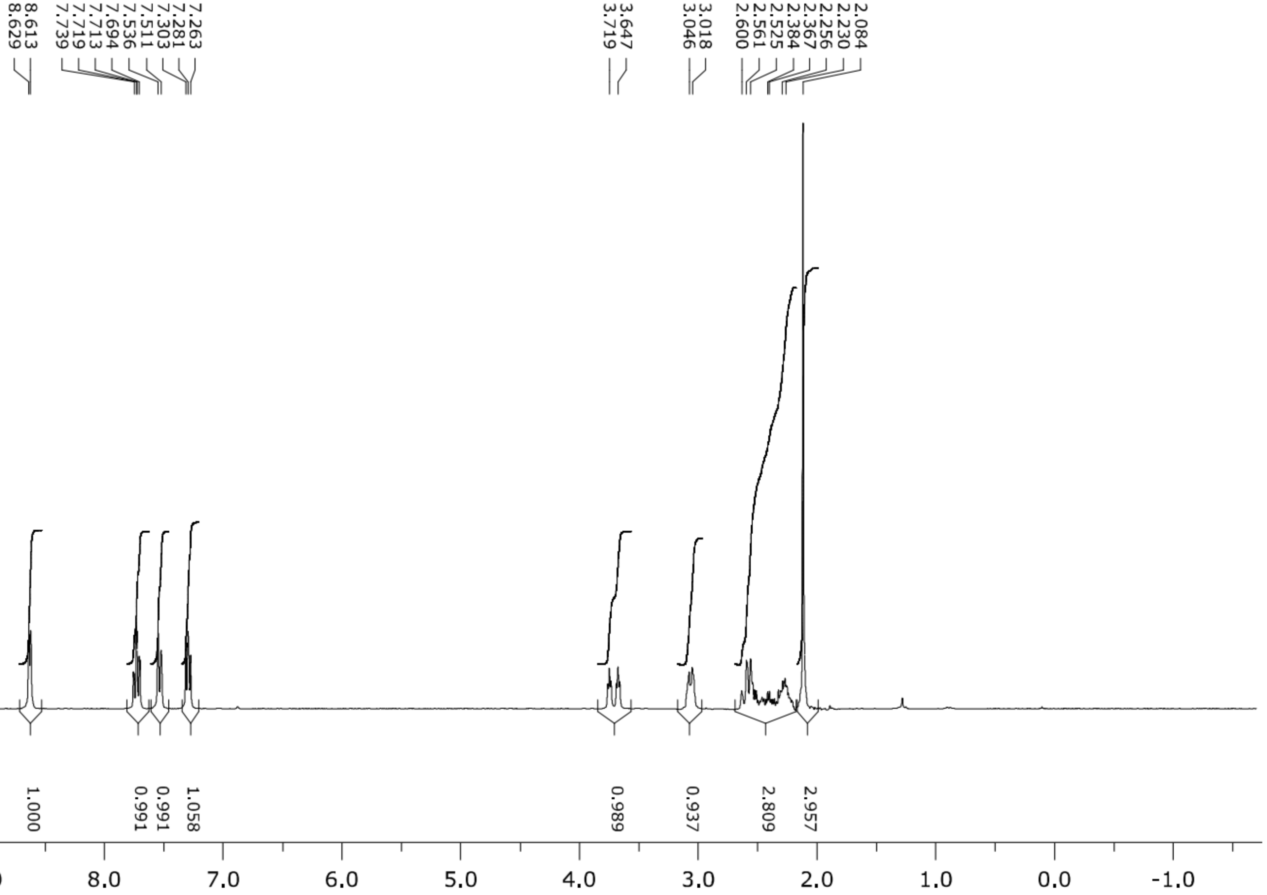

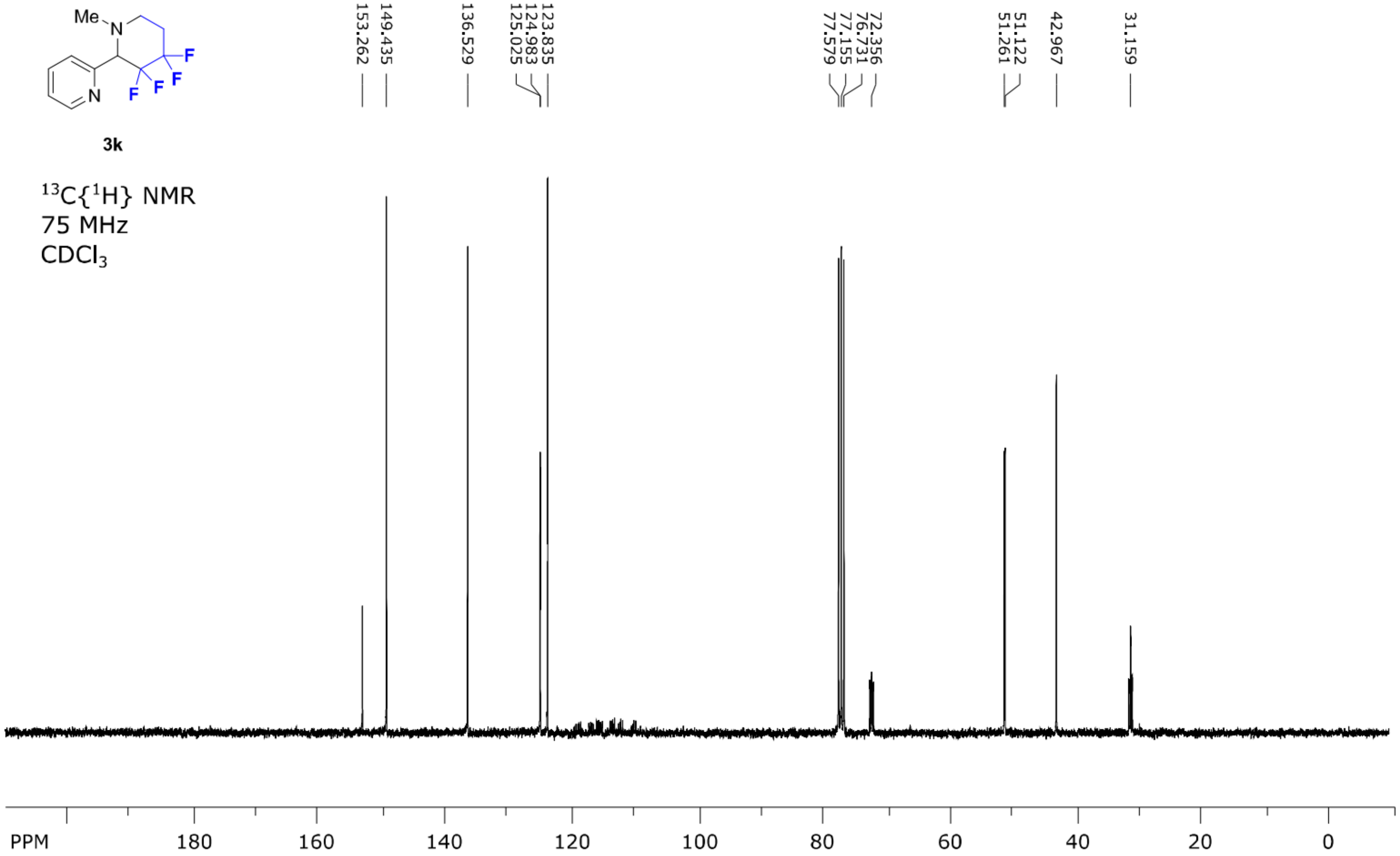
3k
$^{13}C\{^{1}H\}$ NMR
75 MHz
$CDCl_3$
153.262
149.435
136.529
125.025
124.983
123.835
77.579
77.155
76.731
72.356
51.261
51.122
42.967
31.159
PPM
180
160
140
120
100
80
60
40
20
0

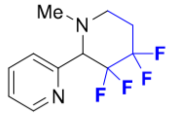

3k

^{19}F NMR
282 MHz
$CDCl_3$

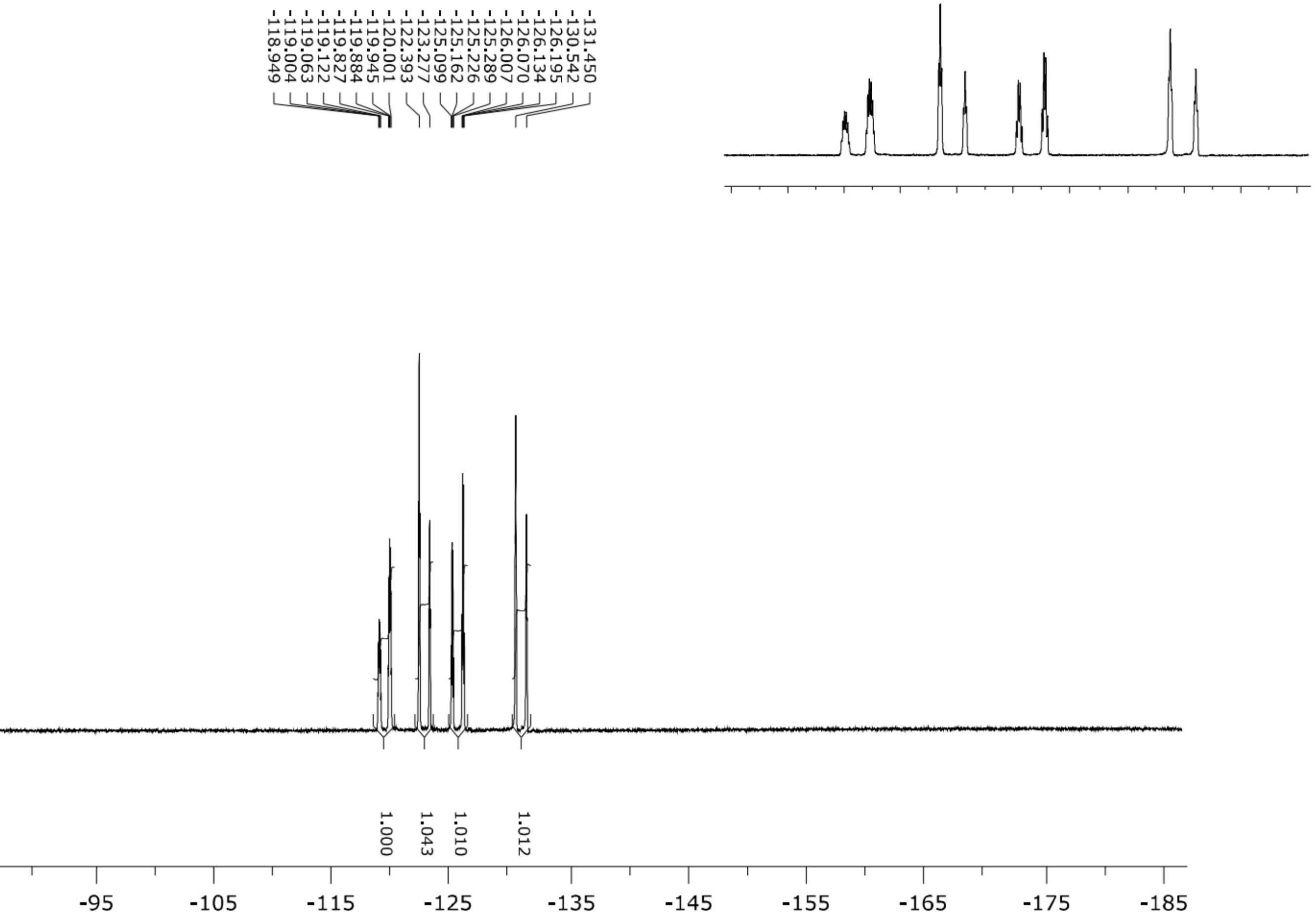

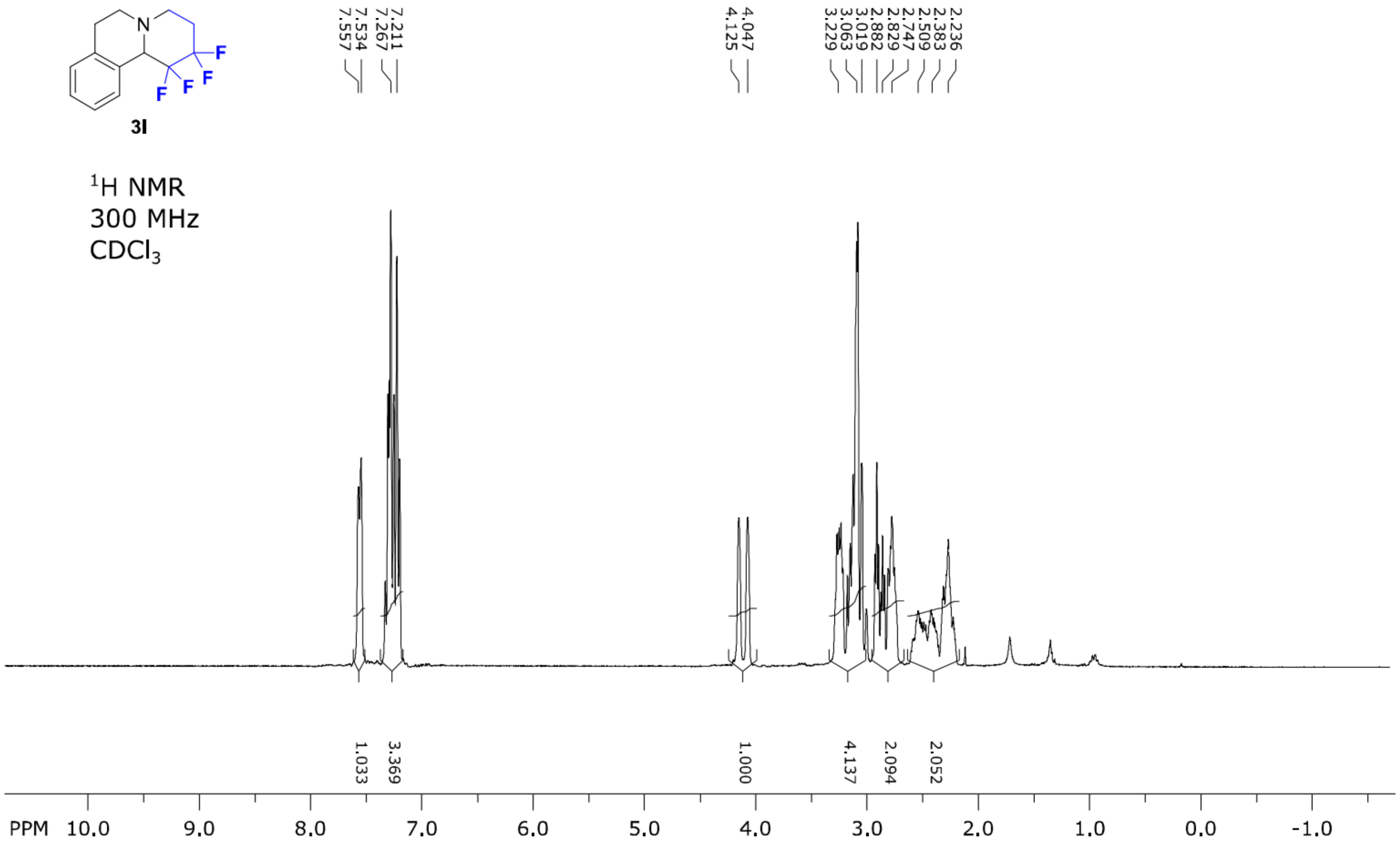
3l
^{1}H NMR
300 MHz
$CDCl_3$
7.557
7.534
7.267
7.211
4.125
4.047
3.229
3.063
3.019
2.882
2.829
2.747
2.509
2.383
2.236
1.033
3.369
1.000
4.137
2.094
2.052
PPM 10.0 9.0 8.0 7.0 6.0 5.0 4.0 3.0 2.0 1.0 0.0 -1.0

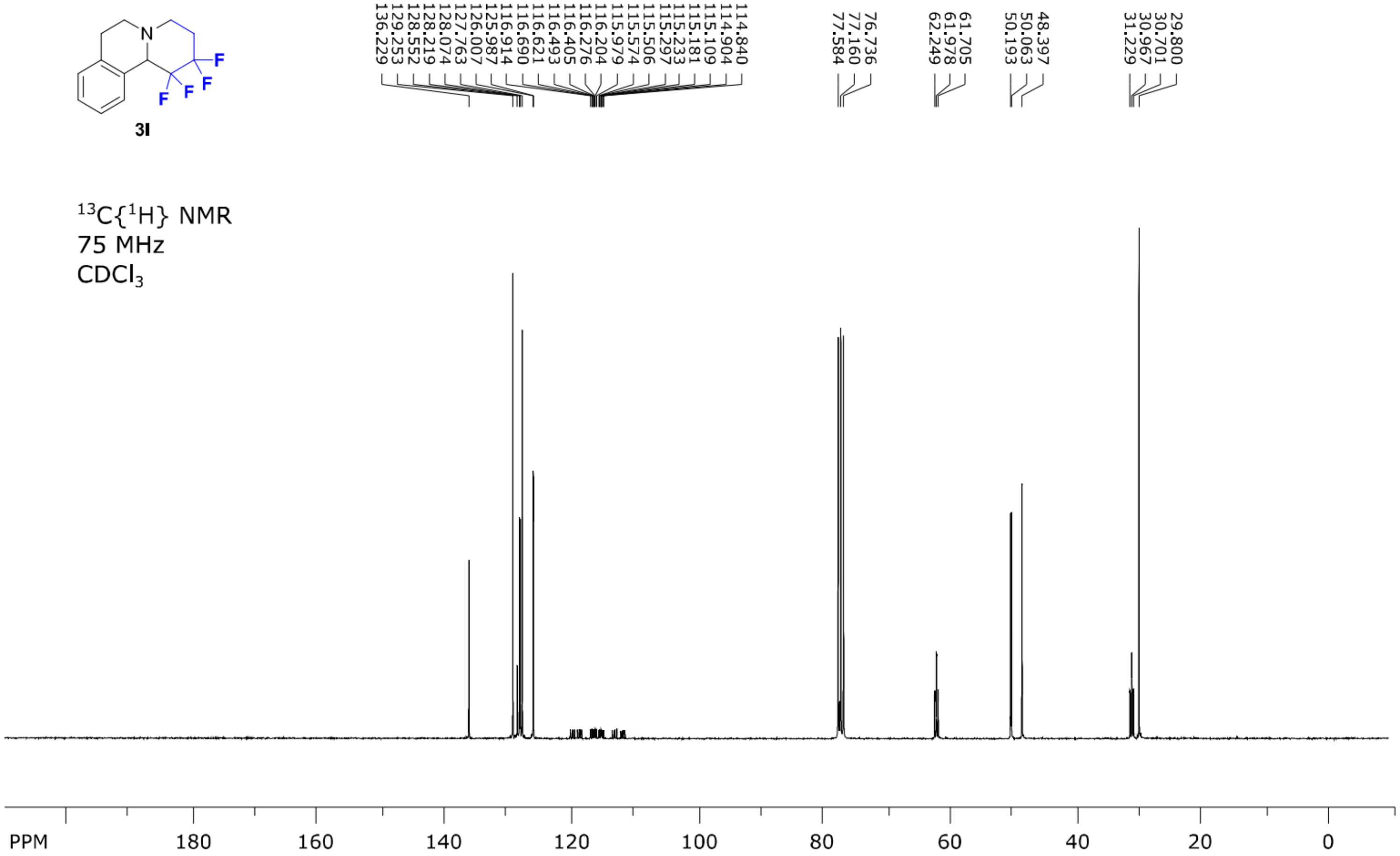

3l
$^{13}C\{^1H\}$ NMR
75 MHz
$CDCl_3$
136.229
129.253
128.552
128.219
128.074
127.763
126.007
125.987
116.914
116.690
116.621
116.493
116.405
116.276
116.204
115.979
115.574
115.506
115.297
115.233
115.181
115.109
114.904
114.840
77.584
77.160
76.736
62.249
61.978
61.705
50.193
50.063
48.397
31.229
30.967
30.701
29.800
PPM
180
160
140
120
100
80
60
40
20
0

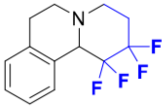

3l

^{19}F NMR
282 MHz
$CDCl_3$

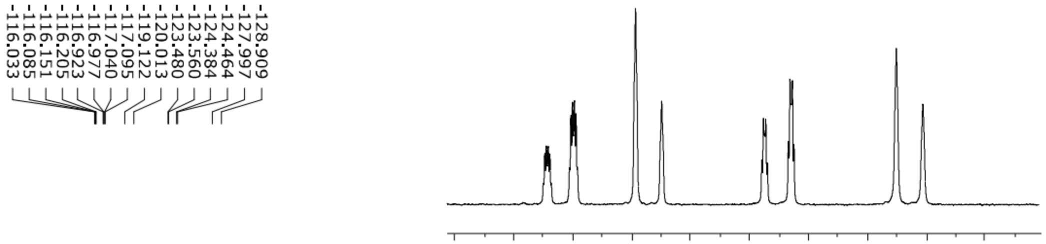
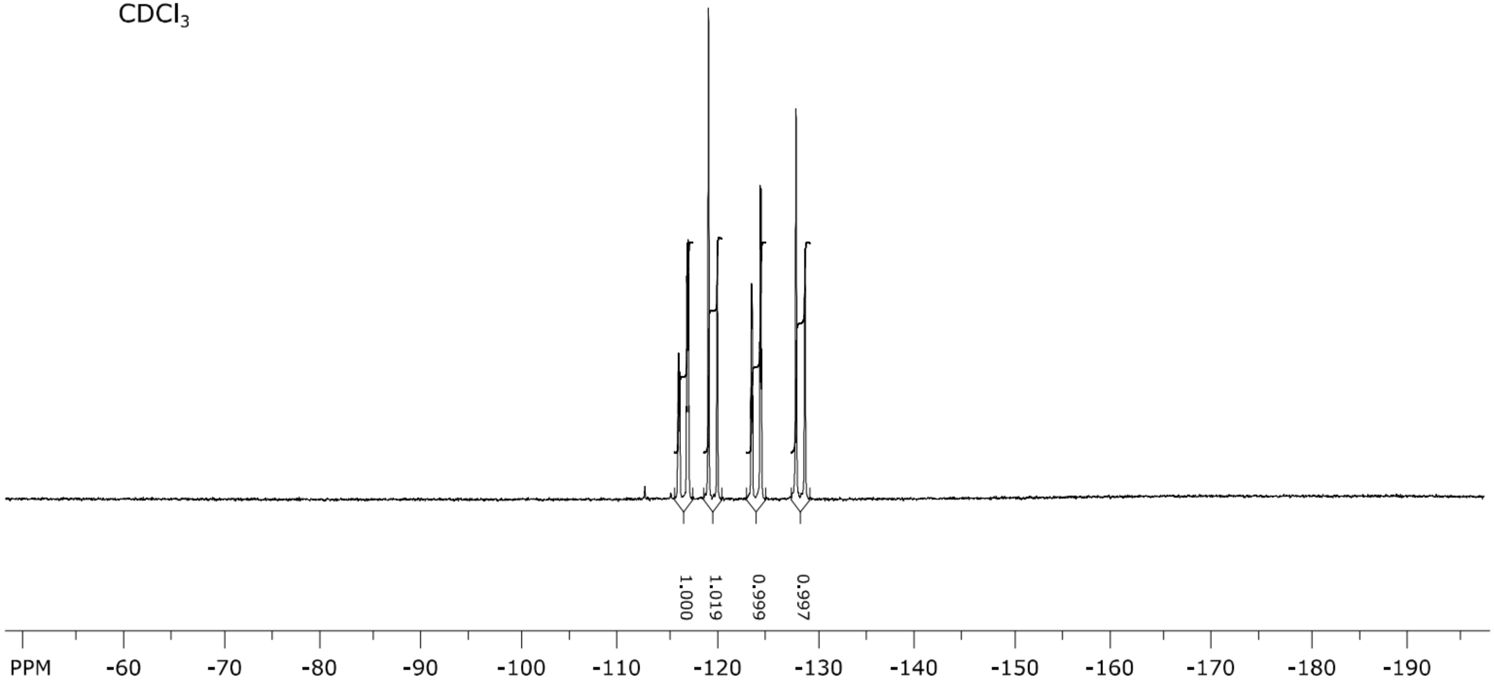

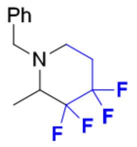

3m

^{1}H NMR
300 MHz
$CDCl_3$

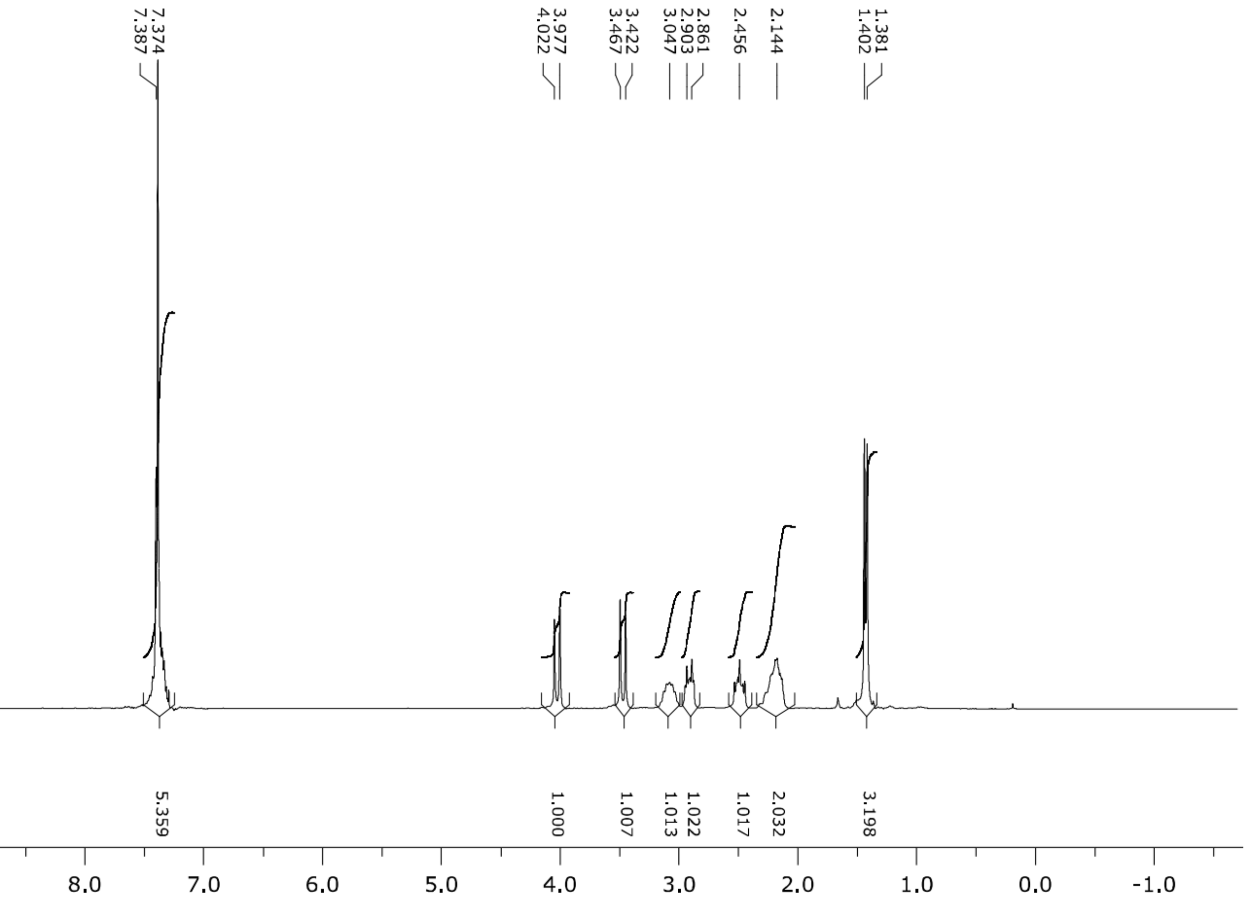

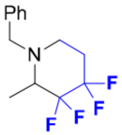

3m

$^{13}C\{^1H\}$ NMR
75 MHz
$CDCl_3$

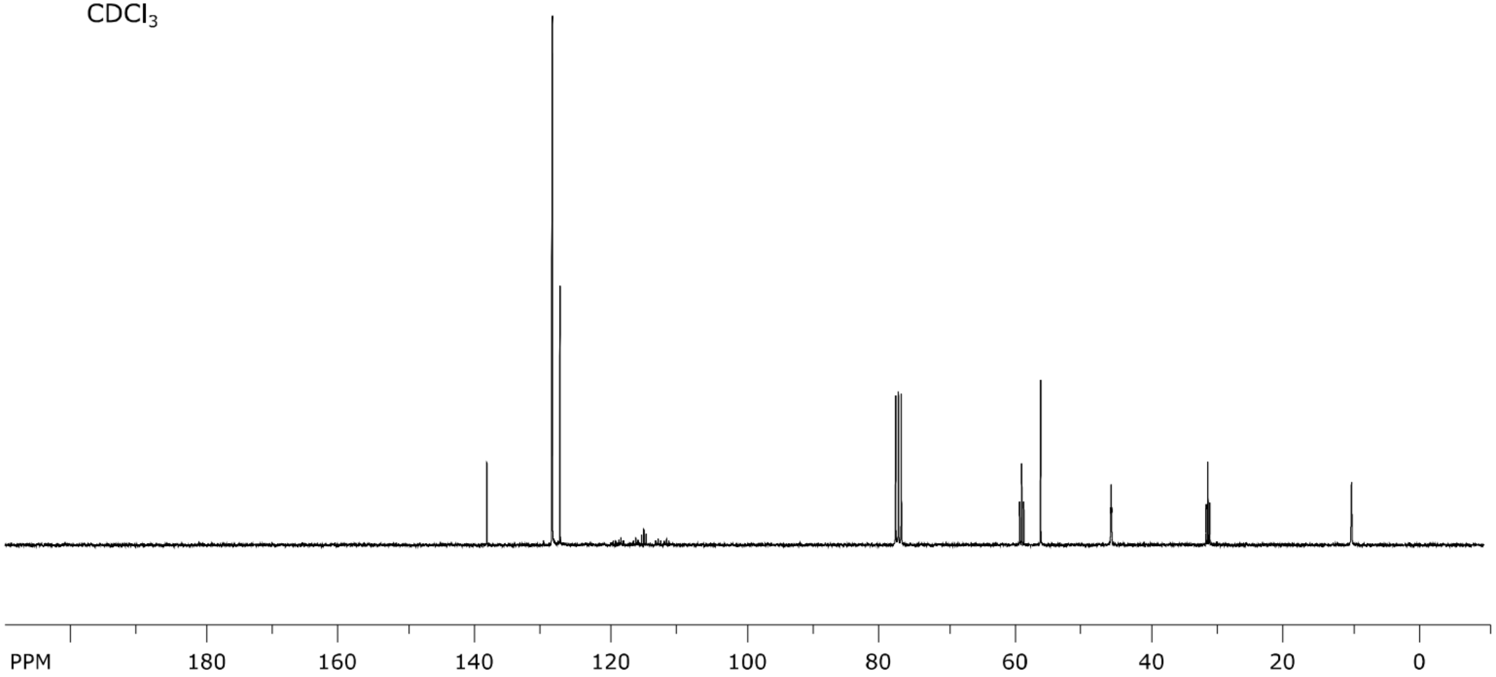

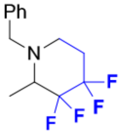

3m

^{19}F NMR
282 MHz
$CDCl_3$

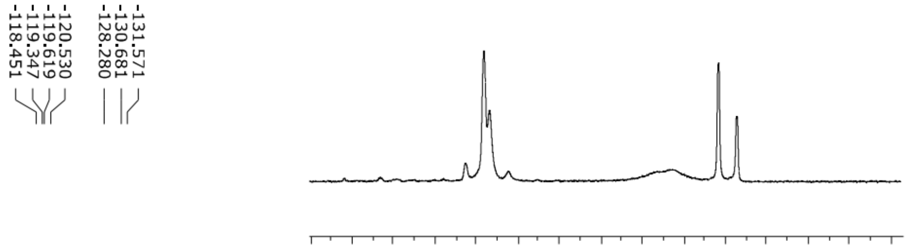

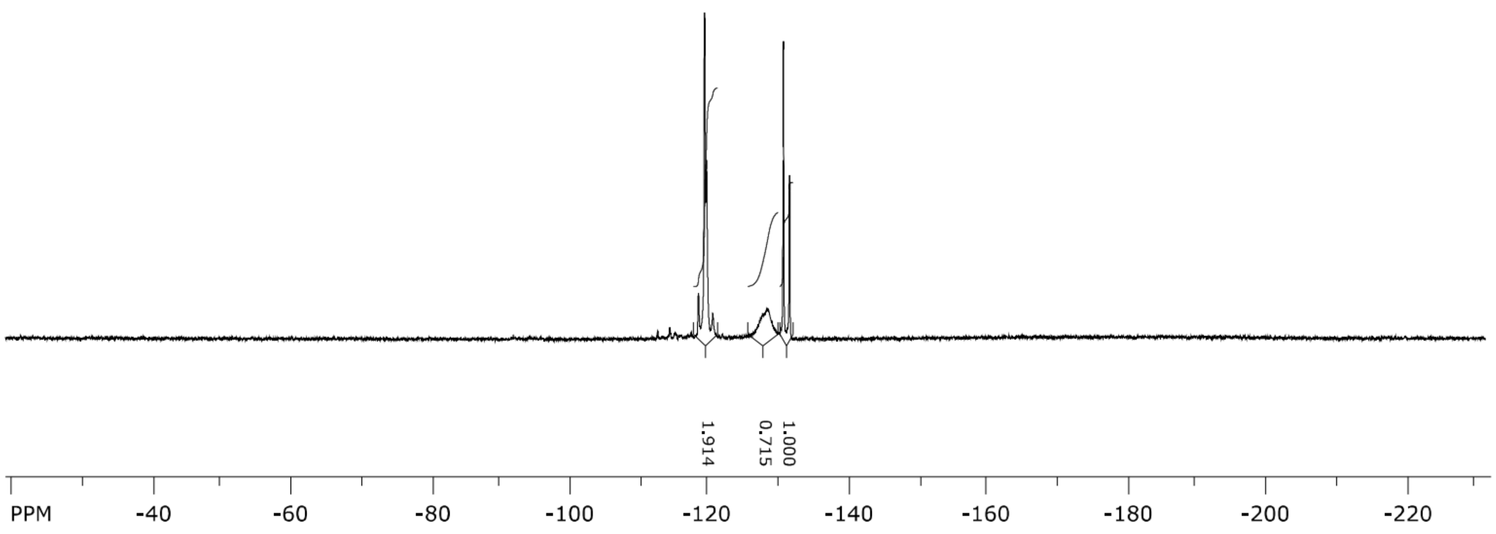

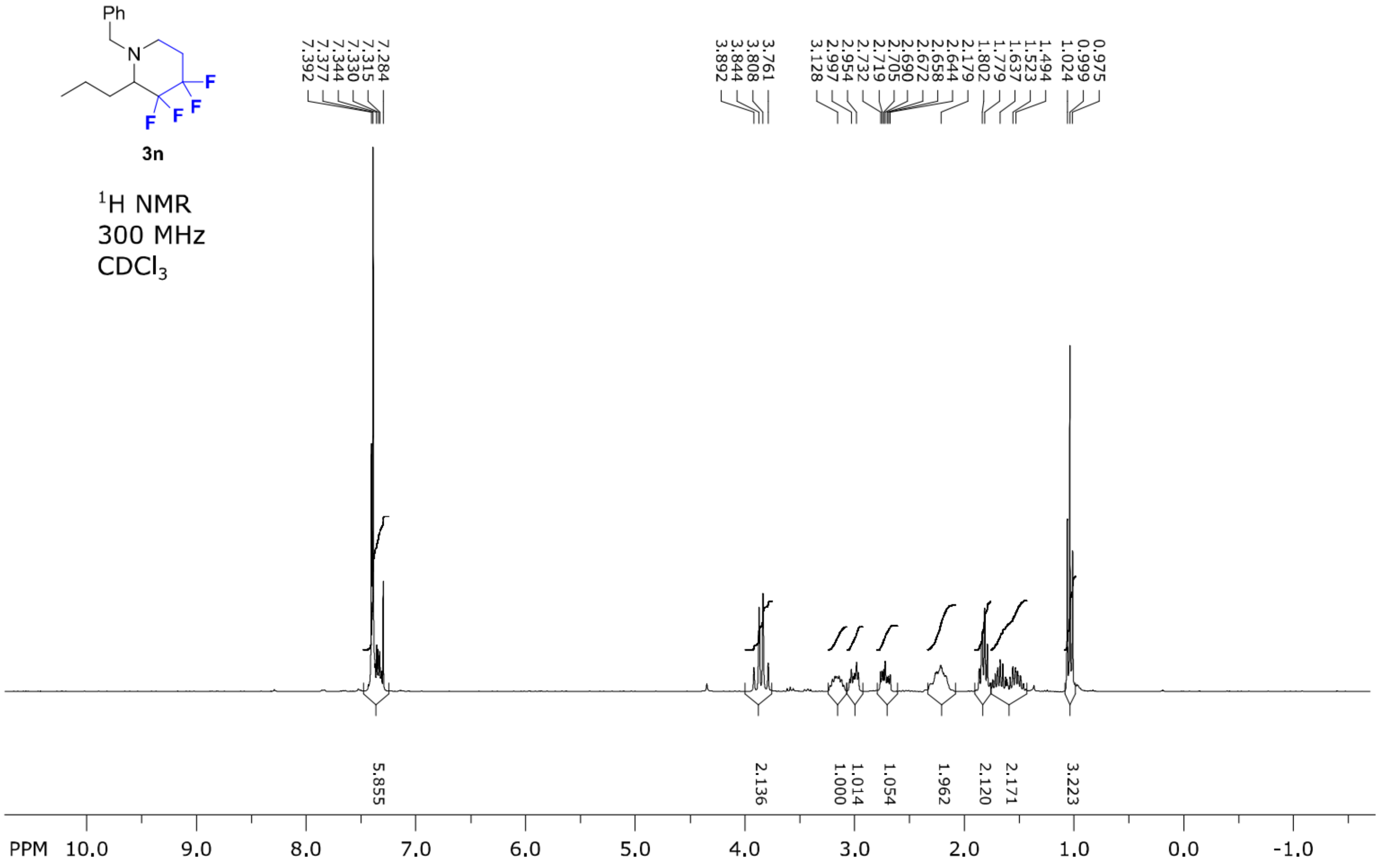

Ph
N
F
F
F
F
3n
^{1}H NMR
300 MHz
CDCl3
7.392
7.377
7.344
7.330
7.315
7.284
3.892
3.844
3.808
3.761
3.128
2.997
2.954
2.732
2.719
2.705
2.690
2.672
2.658
2.644
2.179
1.802
1.779
1.637
1.523
1.494
1.024
0.999
0.975
5.855
2.136
1.000
1.014
1.054
1.962
2.120
2.171
3.223
PPM 10.0 9.0 8.0 7.0 6.0 5.0 4.0 3.0 2.0 1.0 0.0 -1.0

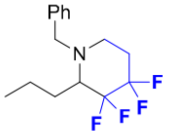

3n

$^{13}C\{^{1}H\}$ NMR
75 MHz
$CDCl_3$

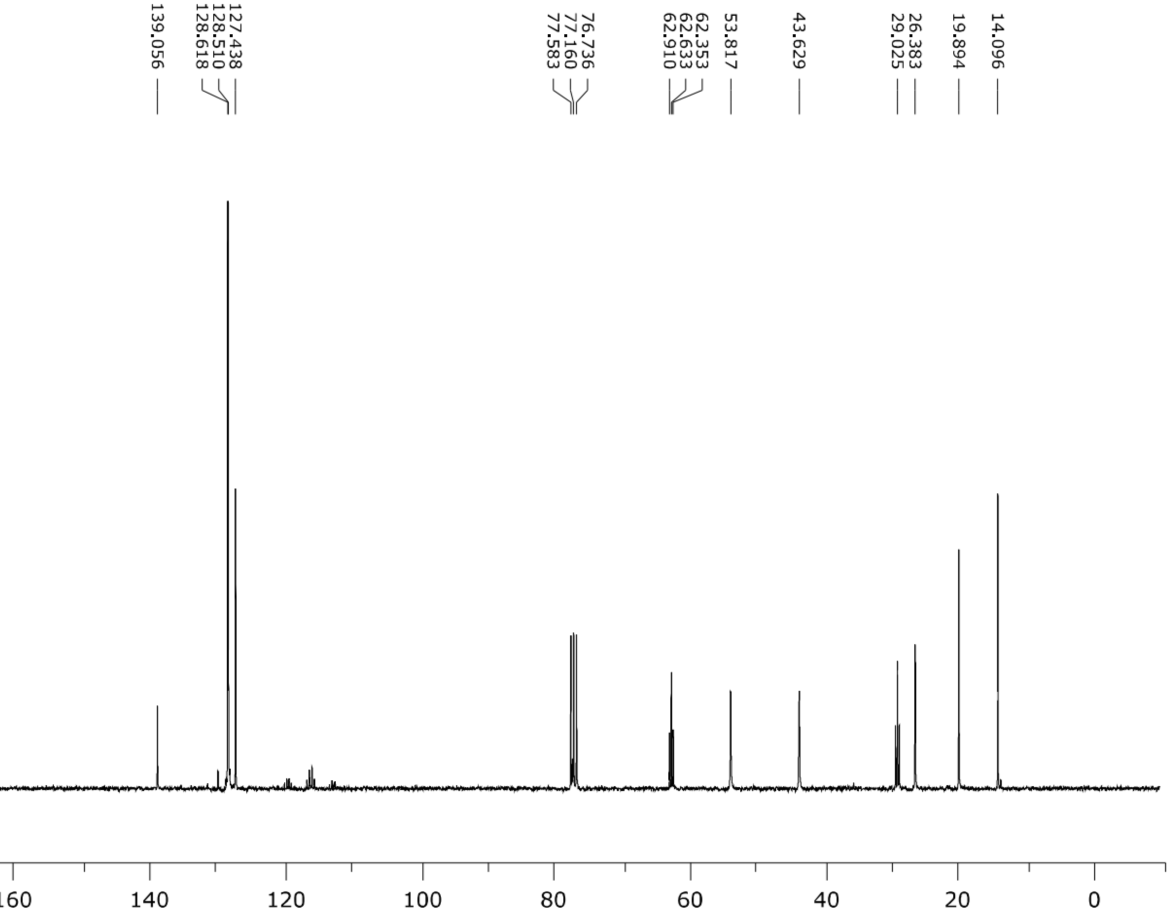

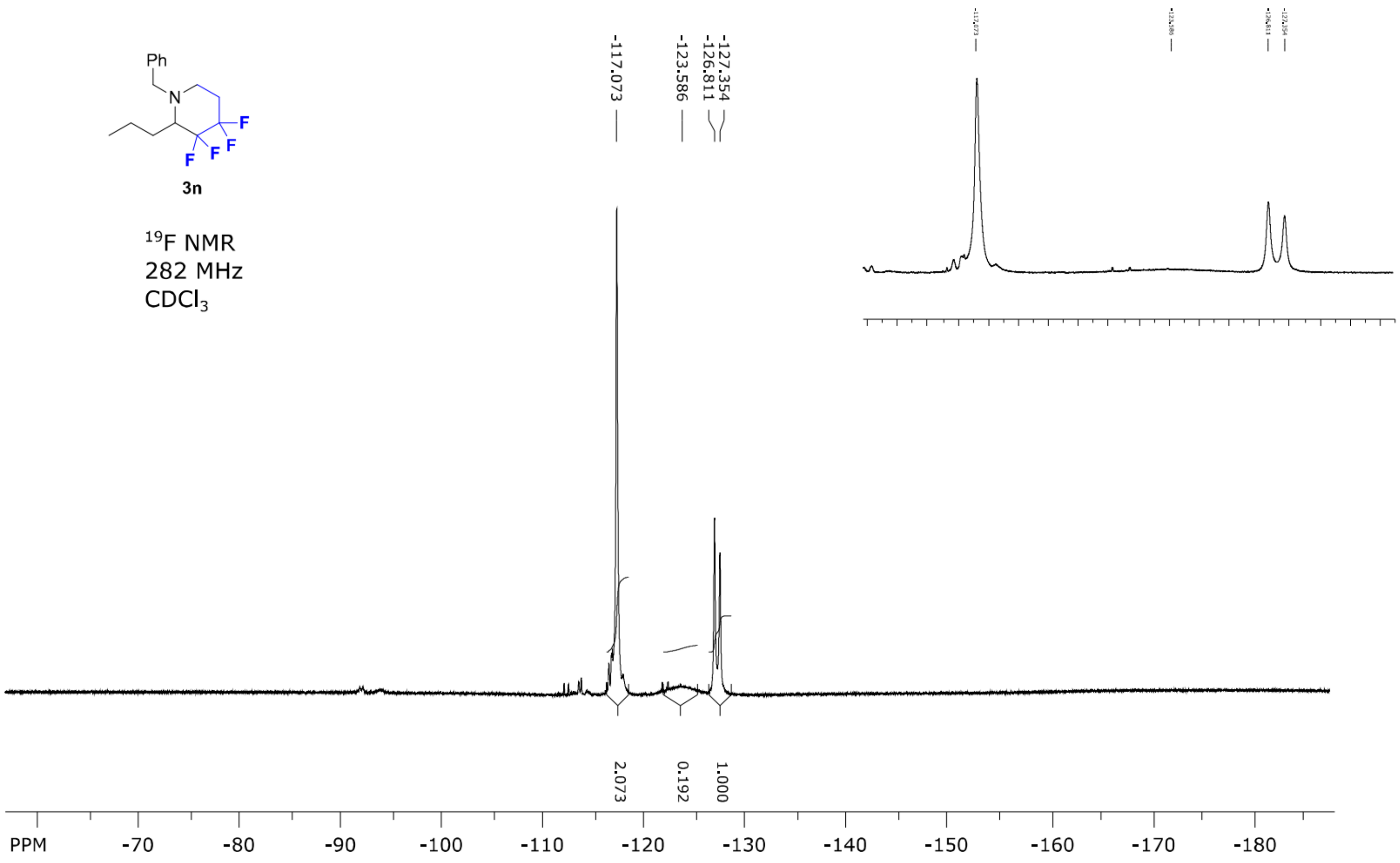
Ph
N
F
F
F
F
3n
^{19}F NMR
282 MHz
$CDCl_3$
-117.073
-123.586
-126.811
-127.354
2.073
0.192
1.000
PPM
-70
-80
-90
-100
-110
-120
-130
-140
-150
-160
-170
-180

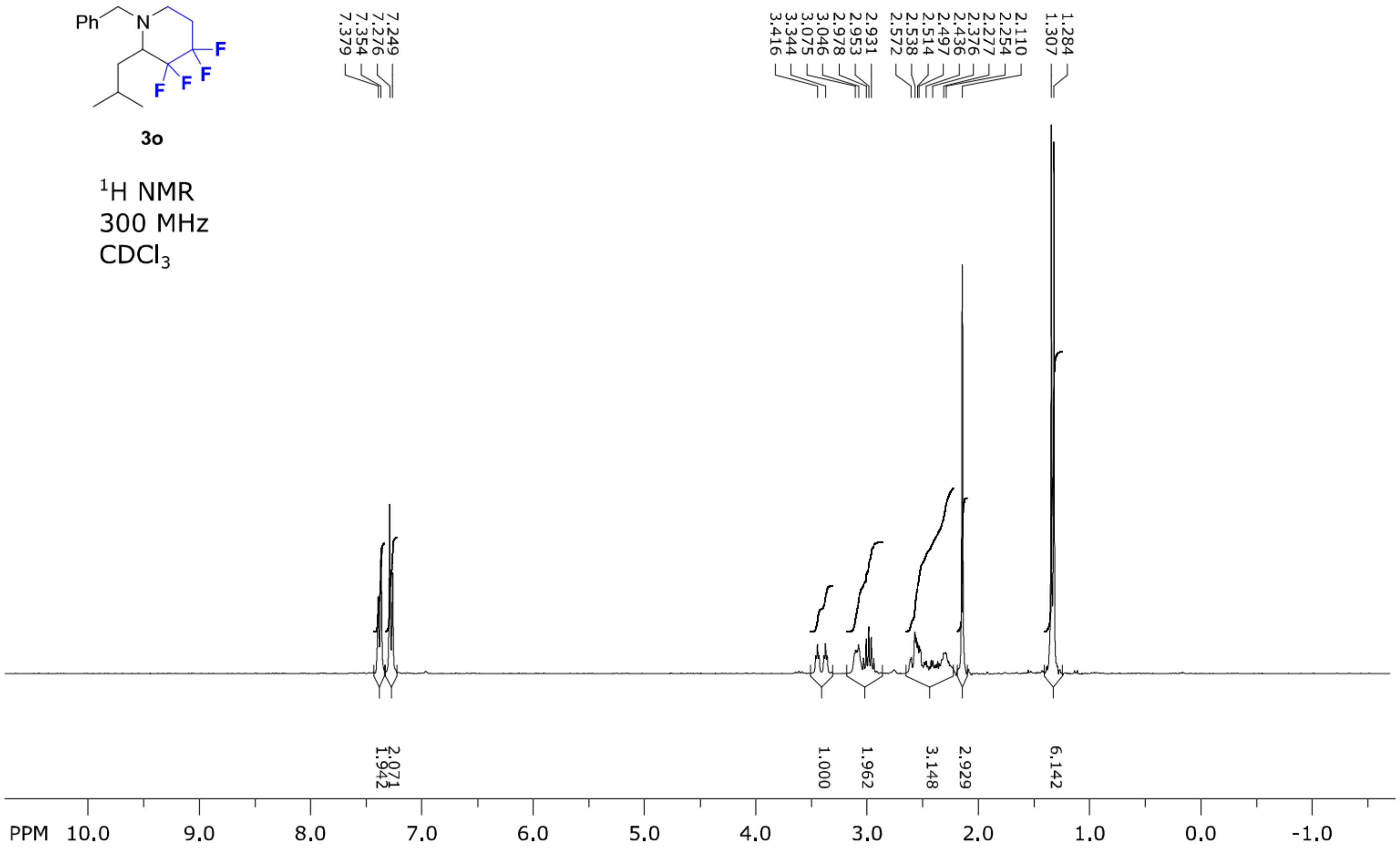
Ph
N
F
F
F
F
3o
1H NMR
300 MHz
CDCl3
7.379
7.354
7.276
7.249
3.416
3.344
3.075
3.046
2.978
2.953
2.931
2.572
2.538
2.514
2.497
2.436
2.376
2.277
2.254
2.110
1.307
1.284
1.942
2.071
1.000
1.962
3.148
2.929
6.142
PPM 10.0 9.0 8.0 7.0 6.0 5.0 4.0 3.0 2.0 1.0 0.0 -1.0

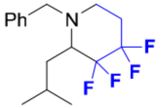

3o

$^{13}C\{^{1}H\}$ NMR
75 MHz
$CDCl_3$

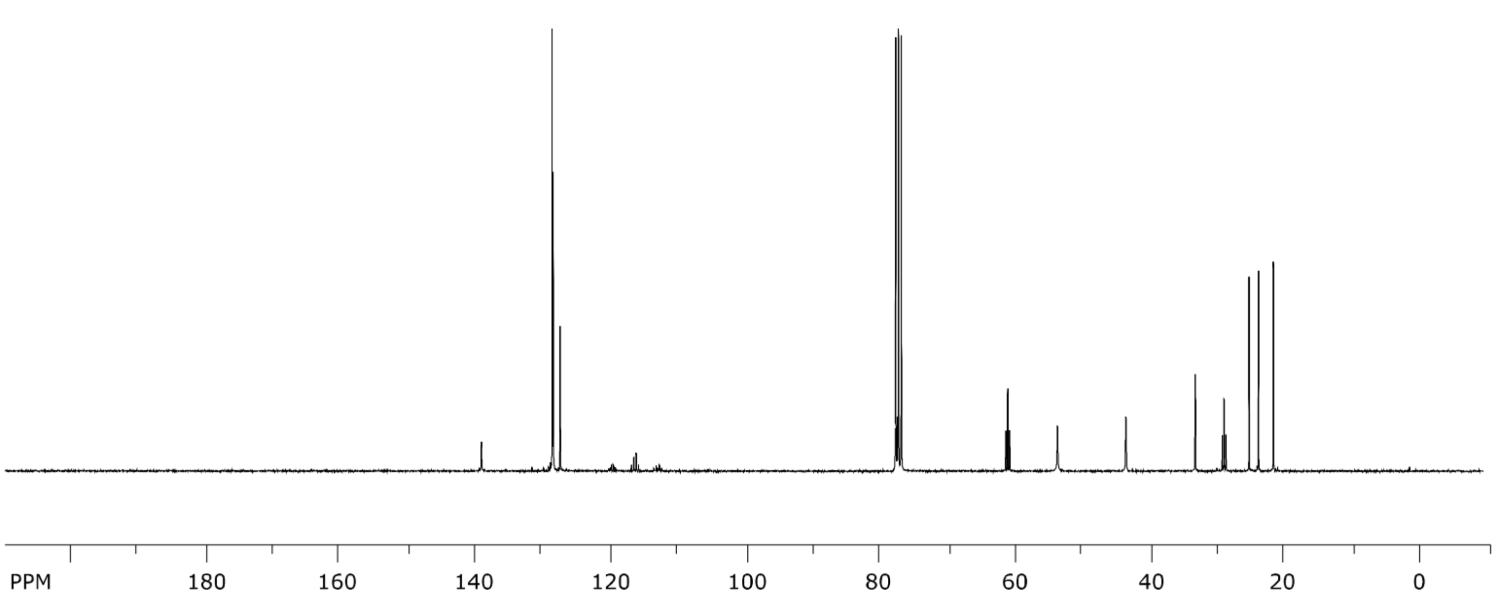

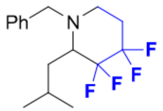

3o

^{19}F NMR
282 MHz
$CDCl_3$

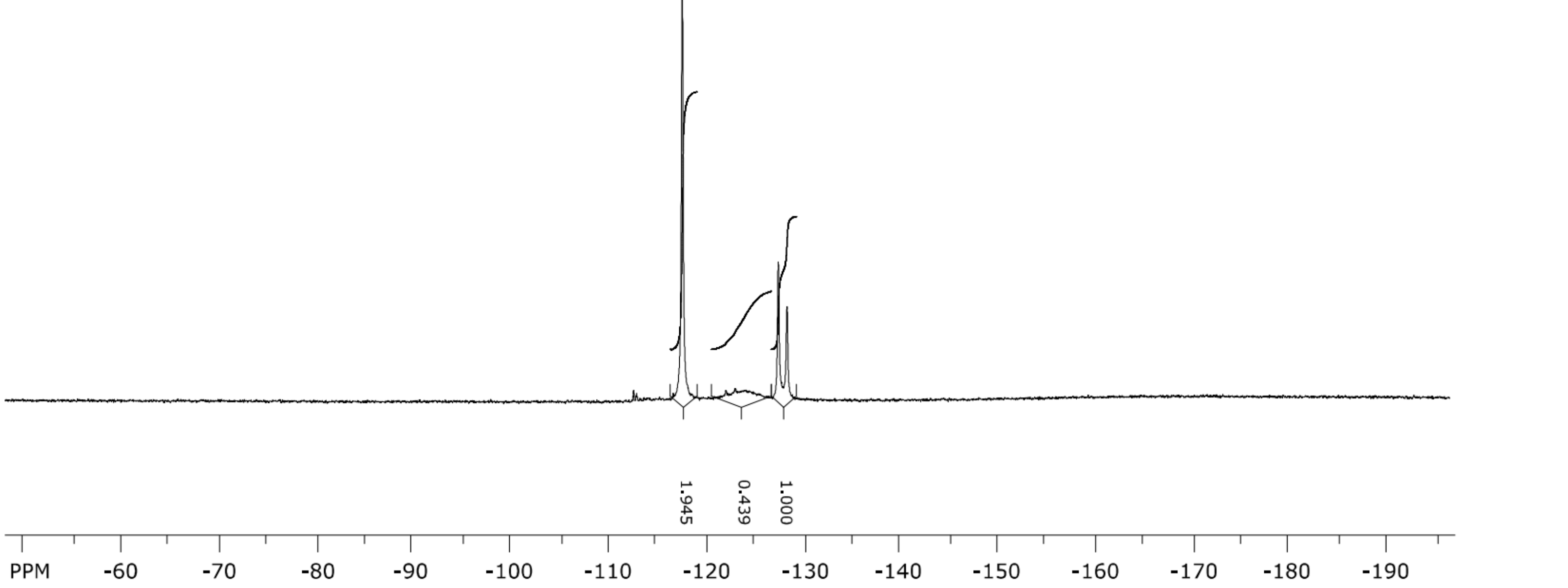

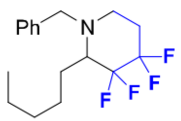

3p

^{1}H NMR
300 MHz
$CDCl_3$

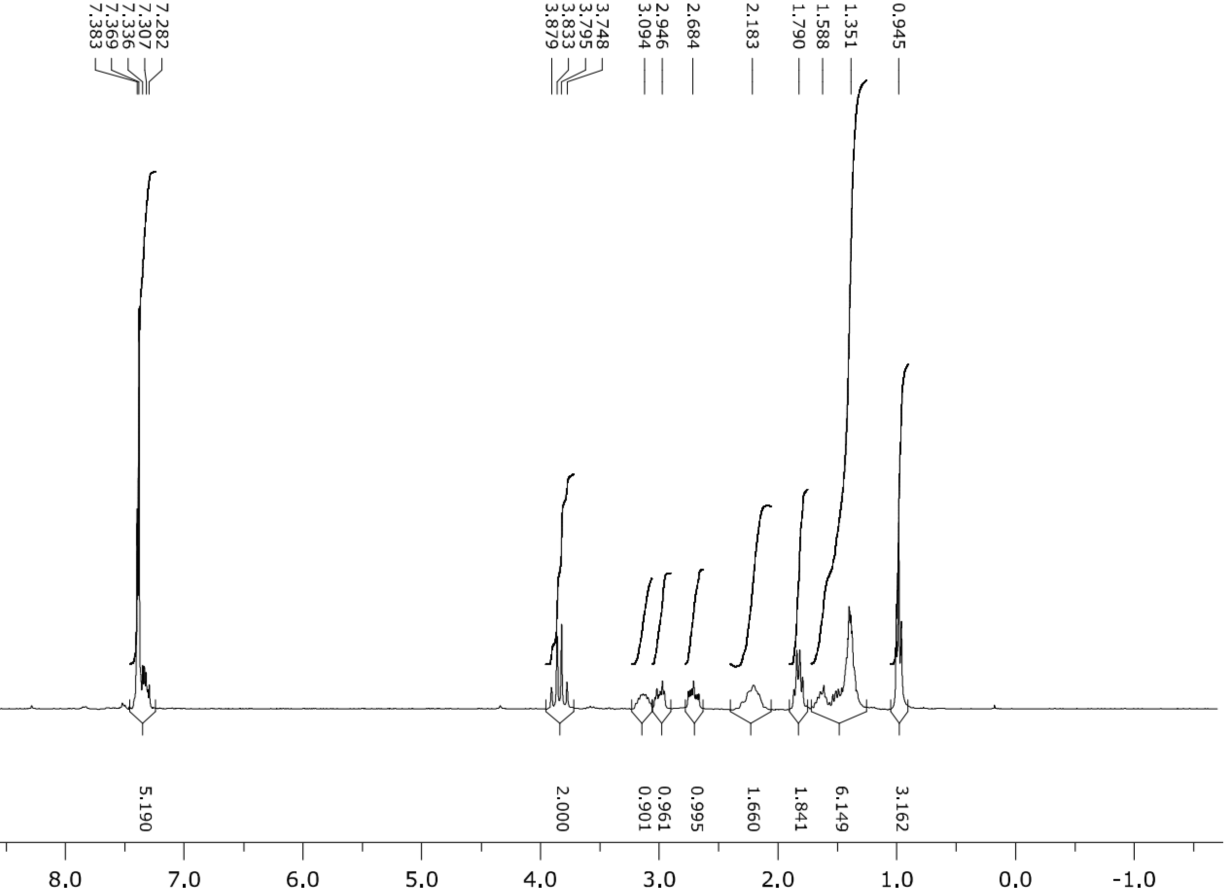

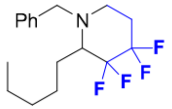

3p

$^{13}C\{^1H\}$ NMR
75 MHz
$CDCl_3$

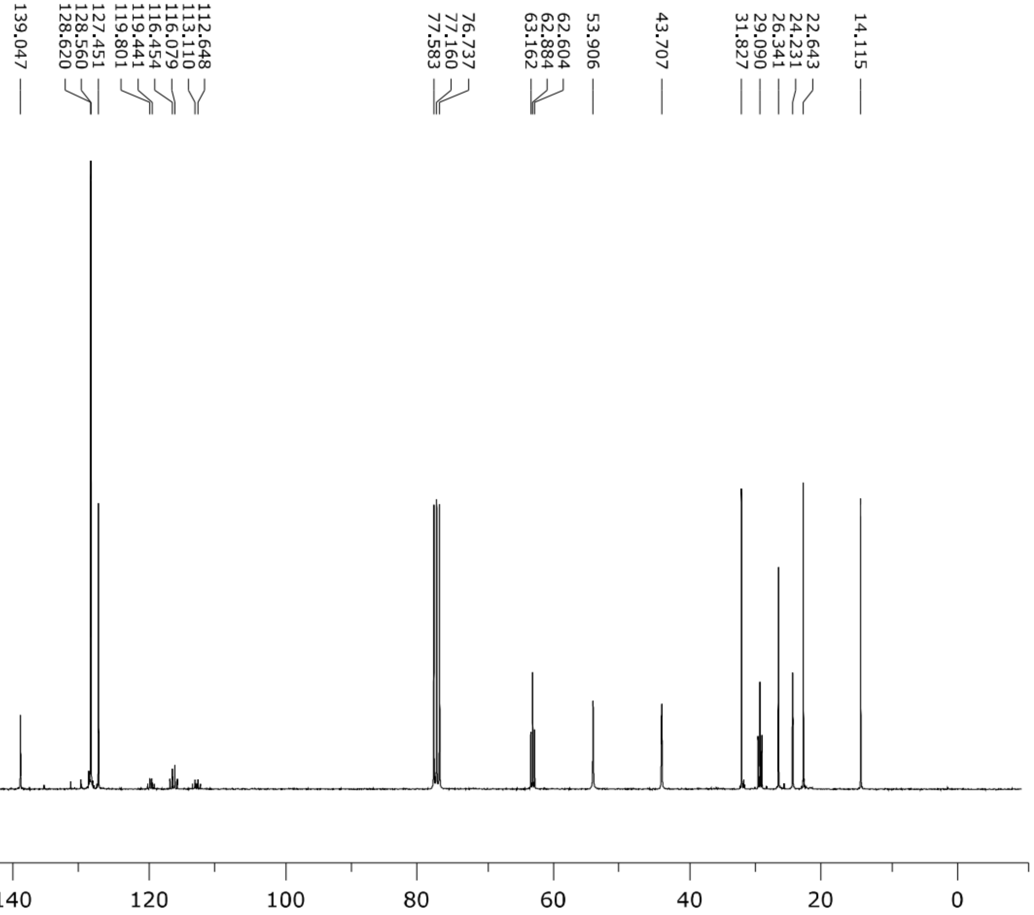

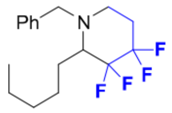

3p

^{19}F NMR
282 MHz
$CDCl_3$

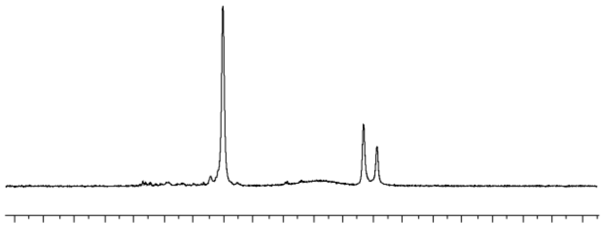

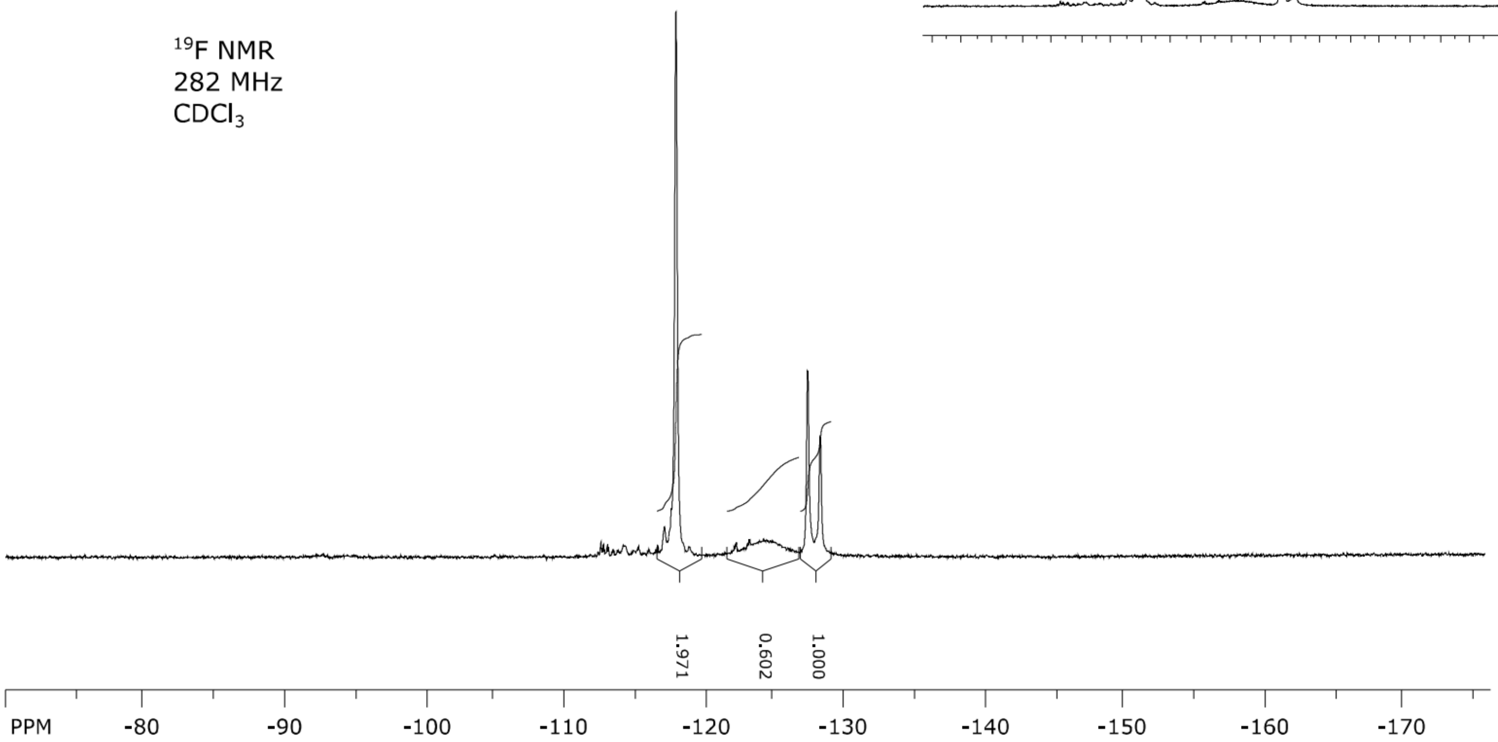

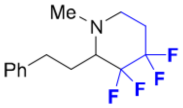

3q

^{1}H NMR
300 MHz
$CDCl_3$

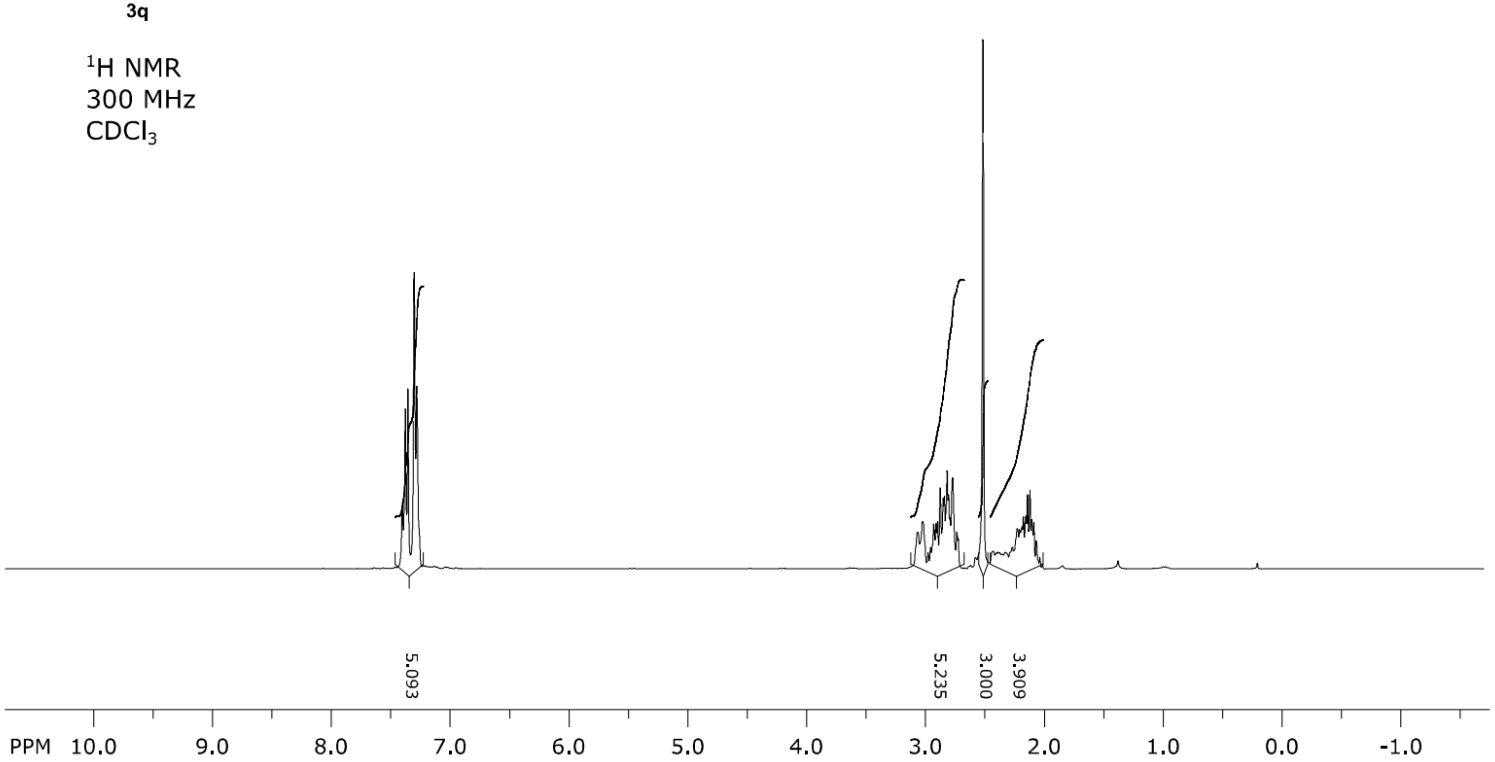

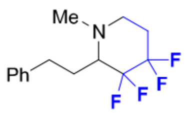

3q

$^{13}C\{^{1}H\}$ NMR
75 MHz
$CDCl_3$

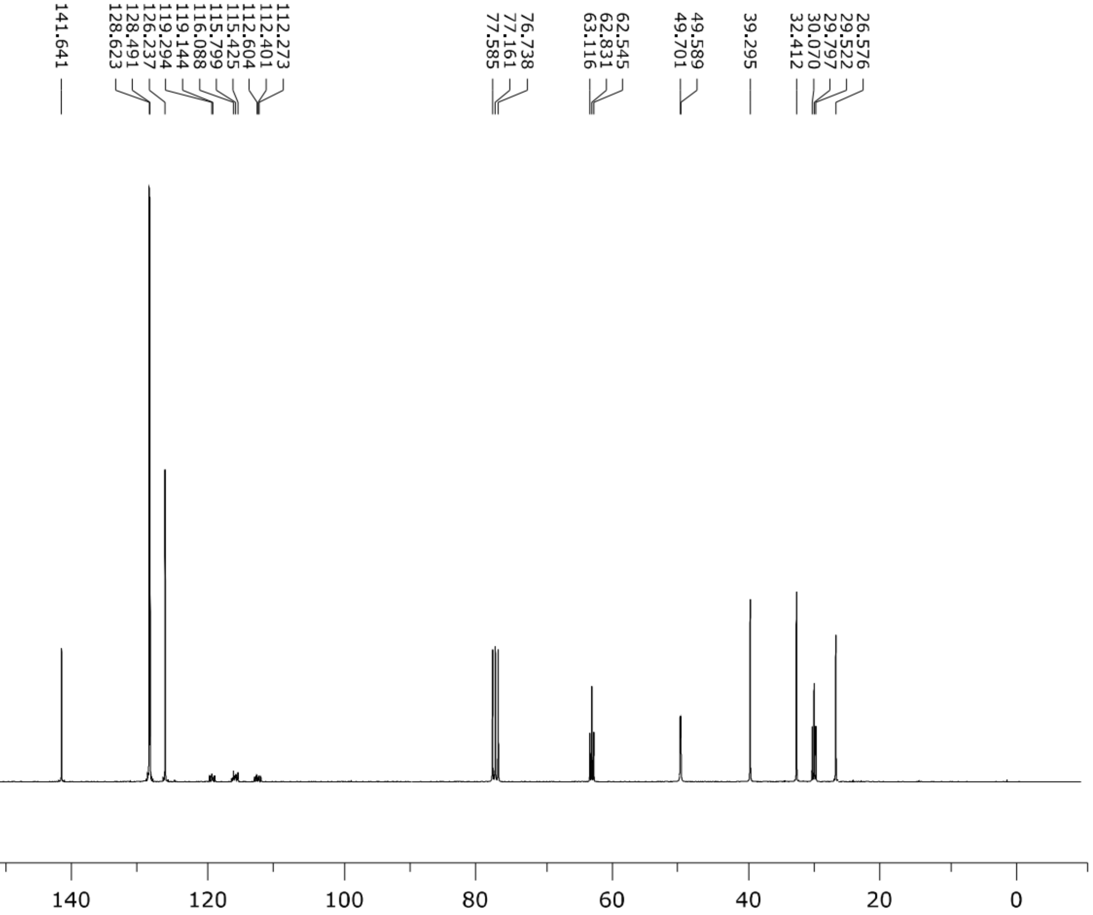

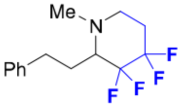

3q

^{19}F NMR
282 MHz
$CDCl_3$

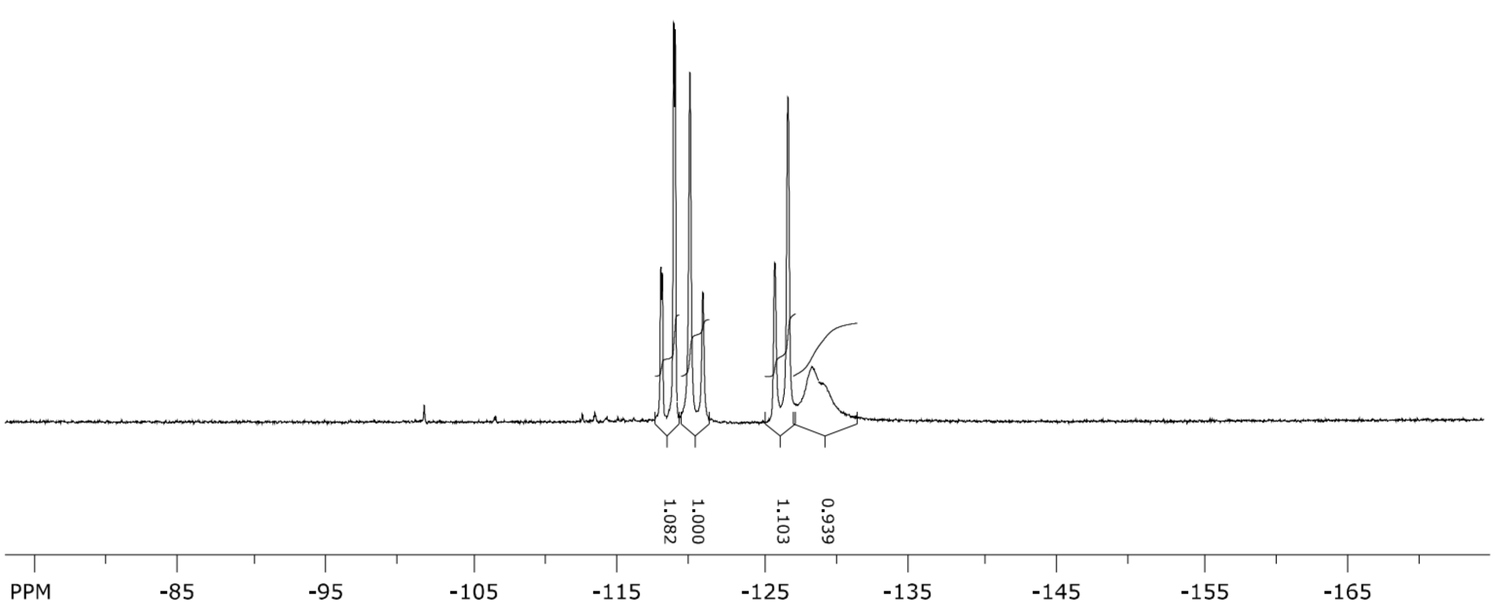

Supplement: File 1 — Full experimental details, compound characterization, X-ray data, and copies of NMR spectra. [file Beilstein_J_Org_Chem-16-3104-s001.pdf]
